# Supplementary material for: Regulatory Compliance in Online Dog Advertisements in Australia
Source: Animals (Basel). 2020 Mar 3;10(3):425. doi: 10.3390/ani10030425 (PMC7142573; doi:10.3390/ani10030425)
Supplement: Supplementary file 1 [file animals-10-00425-s001.zip › Supp Material Costa final/Supp Mat A - Dog Breeds.docx]

Supplementary Material A

**Table A.1.** Number of times dog breeds were advertised from two individual webscrapes from Gumtree ads (March 25th and April 8th, 2019) in Australia. Total of 145 unique breeds represented.

| **Breed** | **Number of ads** | **Percentage** |
| --- | --- | --- |
| American Staffordshire Terrier | 130 | 7.5% |
| French Bulldog | 128 | 7.4% |
| Staffordshire Bull Terrier | 117 | 6.7% |
| Kelpie | 92 | 5.3% |
| German Shepherd | 88 | 5.1% |
| Border Collie | 70 | 4.0% |
| Australian Cattle Dog | 63 | 3.6% |
| Chihuahua | 48 | 2.8% |
| Pug | 47 | 2.7% |
| Bull Arab | 46 | 2.7% |
| Jack Russell Terrier | 46 | 2.7% |
| Cavoodle | 45 | 2.6% |
| Rottweiler | 45 | 2.6% |
| Maltese | 43 | 2.5% |
| Labrador Retriever | 41 | 2.4% |
| Unknown | 30 | 1.7% |
| Toy Poodle | 28 | 1.6% |
| Cavalier King Charles Spaniel | 26 | 1.5% |
| Pomeranian | 26 | 1.5% |
| British Bulldog | 25 | 1.4% |
| Miniature Dachshund | 22 | 1.3% |
| Mastiff | 21 | 1.2% |
| Miniature Fox Terrier | 21 | 1.2% |
| American Bulldog | 19 | 1.1% |
| Pit Bull Terrier | 19 | 1.1% |
| Great Dane | 17 | 1.0% |
| Maremma Sheepdog | 17 | 1.0% |
| Bullmastiff | 15 | 0.9% |
| Rhodesian Ridgeback | 14 | 0.8% |
| Australian Bulldog | 13 | 0.7% |
| Irish Wolfhound | 13 | 0.7% |
| Maltese Shih Tzu | 13 | 0.7% |
| Husky | 12 | 0.7% |
| Beagle | 11 | 0.6% |
| Shar Pei | 11 | 0.6% |
| Siberian Husky | 11 | 0.6% |
| Dogue de Bordeaux | 10 | 0.6% |
| Poodle | 10 | 0.6% |
| Bandog | 9 | 0.5% |
| Boxer | 9 | 0.5% |
| Fox Terrier | 9 | 0.5% |
| Greyhound | 9 | 0.5% |
| Labradoodle | 9 | 0.5% |
| Moodle | 9 | 0.5% |
| Australian Koolie | 8 | 0.5% |
| Dachshund | 8 | 0.5% |
| German Shorthaired Pointer | 8 | 0.5% |
| American Bully | 7 | 0.4% |
| Groodle | 7 | 0.4% |
| Neapolitan Mastiff | 7 | 0.4% |
| English Springer Spaniel | 6 | 0.3% |
| Golden Retriever | 5 | 0.3% |
| Miniature Poodle | 5 | 0.3% |
| Shih Tzu | 5 | 0.3% |
| Spoodle | 5 | 0.3% |
| Staghound | 5 | 0.3% |
| Akita | 4 | 0.2% |
| Boston Terrier | 4 | 0.2% |
| Cane Corso | 4 | 0.2% |
| Chinese Crested Dog | 4 | 0.2% |
| Cocker Spaniel | 4 | 0.2% |
| Dobermann | 4 | 0.2% |
| English Pointer | 4 | 0.2% |
| Frug | 4 | 0.2% |
| Malinois | 4 | 0.2% |
| Whippet | 4 | 0.2% |
| Dalmatian | 3 | 0.2% |
| Johnson Bulldog | 3 | 0.2% |
| Miniature Schnauzer | 3 | 0.2% |
| Pomchi | 3 | 0.2% |
| Pugalier | 3 | 0.2% |
| Rough Collie | 3 | 0.2% |
| Swiss Shepherd | 3 | 0.2% |
| Alaskan Malamute | 2 | 0.1% |
| Australian Shepherd | 2 | 0.1% |
| Australian Silky Terrier | 2 | 0.1% |
| Australian Stumpy Tail Cattle Dog | 2 | 0.1% |
| Beaglier | 2 | 0.1% |
| Belgian Shepherd | 2 | 0.1% |
| Bull Terrier | 2 | 0.1% |
| German Spitz | 2 | 0.1% |
| Harrier | 2 | 0.1% |
| Hound | 2 | 0.1% |
| Miniature Pinscher | 2 | 0.1% |
| Miniature Spoodle | 2 | 0.1% |
| Pembroke Welsh Corgi | 2 | 0.1% |
| Puggle | 2 | 0.1% |
| Samoyed | 2 | 0.1% |
| Smithfield | 2 | 0.1% |
| Tenterfield Terrier | 2 | 0.1% |
| Toy Moodle | 2 | 0.1% |
| West Highland White Terrier | 2 | 0.1% |
| Airedale Terrier | 1 | 0.1% |
| Alaskan Husky | 1 | 0.1% |
| Australian Terrier | 1 | 0.1% |
| Basset Hound | 1 | 0.1% |
| Bichon | 1 | 0.1% |
| Bichoodle | 1 | 0.1% |
| Bulldog | 1 | 0.1% |
| Catahoula | 1 | 0.1% |
| Cavachon | 1 | 0.1% |
| Cavashoo | 1 | 0.1% |
| Cavocker | 1 | 0.1% |
| Chion | 1 | 0.1% |
| Chiweenie | 1 | 0.1% |
| Corgi | 1 | 0.1% |
| Dingo | 1 | 0.1% |
| English Cocker Spaniel | 1 | 0.1% |
| English Mastiff | 1 | 0.1% |
| Frenchton | 1 | 0.1% |
| Goldador | 1 | 0.1% |
| Griffon Bruxellois | 1 | 0.1% |
| Havanese | 1 | 0.1% |
| Japanese Chin | 1 | 0.1% |
| Jug | 1 | 0.1% |
| Kangal Shepherd Dog | 1 | 0.1% |
| Labradogue | 1 | 0.1% |
| Lhasa Apso | 1 | 0.1% |
| Malchi | 1 | 0.1% |
| Maremma | 1 | 0.1% |
| Miniature Australian Bulldog | 1 | 0.1% |
| Miniature Bull Terrier | 1 | 0.1% |
| Miniature Cavoodle | 1 | 0.1% |
| Miniature Jack Russell | 1 | 0.1% |
| Miniature Labradoodle | 1 | 0.1% |
| Miniature Maltese | 1 | 0.1% |
| Murray River Retriever | 1 | 0.1% |
| Newfoundland | 1 | 0.1% |
| Papillon | 1 | 0.1% |
| Pekingese | 1 | 0.1% |
| Pinoodle | 1 | 0.1% |
| Pomatzu | 1 | 0.1% |
| Poochin | 1 | 0.1% |
| Puli | 1 | 0.1% |
| Schnauzer | 1 | 0.1% |
| Schnoodle | 1 | 0.1% |
| Shepsky | 1 | 0.1% |
| Shollie | 1 | 0.1% |
| Southern Bulldog | 1 | 0.1% |
| St. Bernard | 1 | 0.1% |
| Terrier | 1 | 0.1% |
| Toy Cavoodle | 1 | 0.1% |
| Toy Pomeranian | 1 | 0.1% |
| Weimarhund | 1 | 0.1% |
| Yorkshire Terrier | 1 | 0.1% |
| Total | 1735 | 100.0% |
