# Supplementary material for: Regulatory Compliance in Online Dog Advertisements in Australia
Source: Animals (Basel). 2020 Mar 3;10(3):425. doi: 10.3390/ani10030425 (PMC7142573; doi:10.3390/ani10030425)
Supplement: Supplementary file 1 [file animals-10-00425-s001.zip › Supp Material Costa final/Supp Mat B - Pure cross percentages.docx]

Supplementary Material B

**Table A.2.** Percentage pure, cross and designer type for top 20 breeds (not including ‘unknown’) as advertised from two individual webscrapes from Gumtree advertisements (March 25th and April 8th, 2019) in Australia.

|  | Pure (%) | Cross (%) | Designer (%) |
| --- | --- | --- | --- |
| American Staffordshire Terrier | 81 | 19 | 0 |
| French Bulldog | 95 | 4 | 1 |
| Staffordshire Bull Terrier | 62 | 38 | 0 |
| Kelpie | 62 | 38 | 0 |
| German Shepherd | 78 | 20 | 1 |
| Border Collie | 67 | 33 | 0 |
| Australian Cattle Dog | 57 | 43 | 0 |
| Chihuahua | 77 | 23 | 0 |
| Pug | 72 | 28 | 0 |
| Bull Arab | 22 | 78 | 0 |
| Jack Russell Terrier | 85 | 15 | 0 |
| Cavoodle | 0 | 0 | 100 |
| Rottweiler | 71 | 29 | 0 |
| Maltese | 12 | 88 | 0 |
| Labrador Retriever | 78 | 22 | 0 |
| Unknown | 3 | 13 | 0 |
| Toy Poodle | 86 | 14 | 0 |
| Cavalier King Charles Spaniel | 69 | 31 | 0 |
| Pomeranian | 77 | 23 | 0 |
| British Bulldog | 96 | 4 | 0 |
| Miniature Dachshund | 91 | 5 | 5 |
