# Supplementary material for: Regulatory Compliance in Online Dog Advertisements in Australia
Source: Animals (Basel). 2020 Mar 3;10(3):425. doi: 10.3390/ani10030425 (PMC7142573; doi:10.3390/ani10030425)
Supplement: Supplementary file 1 [file animals-10-00425-s001.zip › Supp Material Costa final/Supp Mat C - R code.docx]

Supplementary Material C

R, version 3.6.0 – “Planting of a Tree” (The R Foundation, Vienna, Austria)

#R Code written by Ana Goncalves Costa June 2019 - January 2020

#packages used

x<-c("tidyverse", "tidyr", "plyr", "dplyr", "sjstats", "summarytools","ggplot2","grid", "eeptools", "lubridate", "lubridate")

lapply(x, require, character.only = TRUE)

#library(grid) ##http://felixfan.github.io/stacking-plots-same-x/

x<-c("readxl", "pander", "plotly", "knitr", "rmarkdown", "psych")

lapply(x, require, character.only = TRUE)

install.packages("pacman")

rm(x)

install.packages("epiR")

library(epiR)

##unsure if need this

install.packages("editData")

library(editData)

#files##########

############################################# LOAD FILES FOR ANALYSIS ###########################################################

#load webscrape 1 - original with duplicates

dogs1 <- read.csv("C:/Users/Sofia Costa/OneDrive/Adelaide University Hons/Honours Program/DATA - webscraping/1 Websraping MASTER FILE/Scrape one and two/Dog scraping/1 Dog scrape 25th March/v14march25dog.csv")

dogs1 <- as_tibble(dogs1)

dogs1

#load webscrape 1 with no duplicates ---THIS ONE!

dogs1 <- read_rds("C:/Users/Sofia Costa/OneDrive/Adelaide University Hons/Honours Program/R/dogs1.rds")

dogs1 <- as_tibble(dogs1)

dogs1

#load webscrape 2

dogs2 <- read.csv("C:/Users/Sofia Costa/OneDrive/Adelaide University Hons/Honours Program/DATA - webscraping/1 Websraping MASTER FILE/Scrape one and two/Dog scraping/2 Dog scrape 8th April/v14April8dog.csv")

dogs2 <- as_tibble(dogs2)

dogs2

#load webscrape 2 with no duplicates - THIS ONE!

dogs2 <- read_rds("C:/Users/Sofia Costa/OneDrive/Adelaide University Hons/Honours Program/R/dogs2.rds")

dogs2 <- as_tibble(dogs2)

dogs2

#load dataset with both webscrapes together

#this was before the duplicates were removed - DO NOT USE!!!

#DO NOT USE!!!! finaldogads <-read_csv("C:/Users/Sofia Costa/OneDrive/Adelaide University Hons/Honours Program/DATA - webscraping/1 Websraping MASTER FILE/Scrape one and two/Dog scraping/completedogwebscrapes.csv")

finaldogads <- as_tibble(finaldogads)

finaldogads

#With duplicates removed - DOES NOT HAVE AGE AND GROUPS - DO NOT USE!!

finaldogads <- read_rds("C:/Users/Sofia Costa/OneDrive/Adelaide University Hons/Honours Program/R/finaldogads.rds")

finaldogads

###USE THIS FILE - it has dog ads and duplicates removed - also has age and groupings and breed names fixed &

#also qld state rego = bin OR ankc number

dogads3 <- read_rds("C:/Users/Sofia Costa/OneDrive/Adelaide University Hons/Honours Program/R/dogads3_6_11.rds")

dogads3

dogads3 <- read_csv("C:/Users/Sofia Costa/OneDrive/Adelaide University Hons/Honours Program/R/dogads3_6_11.csv")

dogads3

dogads3whisker <- read_csv("C:/Users/Sofia Costa/OneDrive/Adelaide University Hons/Honours Program/R/dogads3withno22k.csv")

#load chi analysis

chidataframe25_08 <- read_csv("C:/Users/Sofia Costa/OneDrive/Adelaide University Hons/Honours Program/R/chidataframe25_08.csv")

chidataframe25_08

#to save the file your working on as csv

write_csv(dogads3,"C:/Users/Sofia Costa/OneDrive/Adelaide University Hons/Honours Program/R/dogads3_13_08.csv")

#to save the file your working on as rds

write_rds(dogads3,"C:/Users/Sofia Costa/OneDrive/Adelaide University Hons/Honours Program/R/dogads3_23_08.rds")

#multivariable regression model

dogadsra_d <- read_csv("C:/Users/Sofia Costa/OneDrive/Adelaide University Hons/Honours Program/R/dogadsra_d.csv")

dogadsra_d

#####################deleting duplicates####################

#ads that have the same state, breed and dob will be considered duplicate

dogads3 %>%

dplyr::count(dob, breed, state) %>%

ggplot(aes(n)) + geom_bar()

#to see how many lines have repeats

dogads3 %>%

dplyr::count(dob, breed, state) %>%

filter(n > 1) %>%

arrange(dob, breed)

#functin to clean up

dogs2 <-

dogs2 %>%

group_by(dob, breed, state) %>%

arrange(date_downloaded) %>%

mutate(n = row_number()) %>%

filter(n == max(n))

#check there are no other duplicates

dogads3 %>%

dplyr::count(dob, breed, state) %>%

filter(n > 1)

dogs2

#now save the file with no duplicates

write_rds(dogs2, "C:/Users/Sofia Costa/OneDrive/Adelaide University Hons/Honours Program/R/dogs2.rds")

#asave as csv

write_csv(dogads3,"C:/Users/Sofia Costa/OneDrive/Adelaide University Hons/Honours Program/R/dogads3_13_08.csv")

#######################################################WEBSCRAPE 1: DESCRIPTORS #####################################

#number of ads in this dataset

adstotal1 <- table(dogs1$state)

adstotal1

#number of unique breeds in this dataset

numberofbreedsdogs1 <- length(unique(dogs1$breed))

numberofbreedsdogs1

#list of all the breeds and how many ads per breed

totalperbreed1 <- table(dogs1$breed)

totalperbreed1

write.csv(totalperbreed1, file = "totalperbreed1.csv", row.names = TRUE)

#number of dogs available for sale in this dataset

#find the total of a column if you add all numbers up

#sum(dataframe$column)

#sum of dogs availabl if we ignore all na's

#dogs available

sum(dogs1$dogs_available, na.rm = TRUE)

#number of dogs available if we presume na'a are average litter size (see below for average calculations)

###total dogs available with na's assumed average litter size #############################

totaldogs_withavena1 <- dogs1[, "dogs_available"]

totaldogs_withavena1

#replace na's with the average litter size (4.419118)

totaldogs_withavena1[is.na(totaldogs_withavena1)] <- 4.431818

totaldogs_withavena1

sum(totaldogs_withavena1)

#find average size of litters

#= create a new dataset with only litters and find ave of that data set

litternumber1 <- dogs1[, "dogs_available"]

#this gives us 907 ads

#remove na's

litternumber1 <- na.omit(litternumber1)

litternumber1

#this leaves us 820 ads

#remove non-litter sales

litternumber1 <- filter(litternumber1, dogs_available > 1)

litternumber1

#this leaves us with 408 ads (511 are single sales)

#calculate average

averagelittersize1 <-colMeans(litternumber1, na.rm = TRUE)

averagelittersize1

################################################################WEBSCRAPE 2: DESCRIPTORS ####################################

#number of ads in this dataset per state

adstotal2 <- table(dogs2$state)

adstotal2

#number of unique breeds in this dataset

numberofbreedsdogs2 <- length(unique(dogs2$breed))

numberofbreedsdogs2

#how many dogs of each breed in this dataset

totalperbreeddogs2 <- table(dogs2$breed)

totalperbreeddogs2

#number of dogs available for sale in this dataset

#find the total of a column if you add all numbers up

#sum(dataframe$column)

#sum of dogs availabl if we ignore all na's

#dogs available

sum(dogs2$dogs_available, na.rm = TRUE)

#number of dogs available if we presume na'a are average litter size (see below for average calculations)######

###total dogs available with na's assumed average litter size #############################

totaldogs_withavena2 <- dogs2[, "dogs_available"]

totaldogs_withavena2

#replace na's with the average litter size (4.419118)

totaldogs_withavena2[is.na(totaldogs_withavena2)] <- 4.090439

totaldogs_withavena2

sum(totaldogs_withavena2)

#####find average size of litters#########

#= create a new dataset with only litters and find ave of that data set

litternumber2 <- dogs2[, "dogs_available"]

litternumber2

sum(is.na(litternumber2))

#remove na's

litternumber2 <- na.omit(litternumber2)

litternumber2

#remove non-litter sales

litternumber2 <- filter(litternumber2, dogs_available > 1)

litternumber2

#calculate average

averagelittersize2 <-colMeans(litternumber2, na.rm = TRUE)

averagelittersize2

############comparison of scrape 1 and 2#################

#########################################################TOTAL DODG ADS (BOTH WEBSCRAPES)############################

########changing dob columns to date - finding out dog ages and putting them into groups#####

#DATAFILE/SET WE WILL USE.

view(finaldogads)

#turn dob into date object

dogads3 <-

finaldogads %>%

mutate(dob = dmy(dob))

#turn date_listed into date object

dogads3 <-

dogads3 %>%

mutate(date_listed = dmy(date_listed))

#check it worked

dogads3 %>%

select(dob, date_listed)

#dogads3 is now the new file we will use going forward.

#now save the file (no duplicates AND has ages)

write_rds(dogads3, "C:/Users/Sofia Costa/OneDrive/Adelaide University Hons/Honours Program/R/finaldogads3.rds")

#Get the time interval between DOB and date listed

dogads3 <-

dogads3 %>%

mutate(

age = date_listed - dob

)

#check worked

dogads3 %>%

select(date_listed, dob, age)

#yes

#This is an interval - I am going to convert to a number to make easier to deal with

dogads3 <-

dogads3 %>%

mutate(

age = age / ddays(1)

)

#check worked

dogads3 %>%

select(date_listed, dob, age)

#Now we can convert these to classes (this creates a column with the diff groups

#assigned to each row appropriately-- incl. all na's to 'unknown')

dogads3 <-

dogads3 %>%

mutate(

age_bin = case_when(

age < 8*7 ~ "< 8 weeks old",

age < 6 * 4 * 7 ~ "8 weeks - 6 months",

age < 365 ~ "6 months - 1 year",

age < 3 * 365 ~ "1 - 3 years",

age < 7 * 365 ~ "3 - 7 years",

is.na(age) ~ "Unknown",

TRUE ~ "> 7 years"

)

)

#Let R know correct order

dogads3 <-

dogads3 %>%

mutate(

age_bin = factor(age_bin,

levels = c("< 8 weeks old","8 weeks - 6 months",

"6 months - 1 year", "1 - 3 years",

"3 - 7 years", "> 7 years", "Unknown")

)

)

#find if any dogs were not given a bin.

dogads3 %>% filter(is.na(age_bin)) %>%

select(date_listed, dob, age, age_bin)

#plot to ensure that doing the right thing

dogads3 %>% ggplot(aes(age, age_bin)) + geom_point()

## Warning: Removed 61 rows containing missing values (geom_point).

#########age descriptors#########

#frequncy of ads in each bin

agegroups <- count(dogads3$age_bin)

agegroups

#how many times age bin appears per state

agesperstate <- count(dogads3, c("state", "age_bin"))

agesperstate

table(dogads3$offered_by, dogads3$age_bin)

#create a histogram of ages

#########################grouping states#################

dogads3 <-

dogads3 %>%

mutate(

state_bin = case_when(

state == "SA" ~ "SA",

state == "NSW" ~ "NSW",

state == "QLD" ~ "QLD",

state == "ACT" ~ "ACT",

state == "VIC" ~ "VIC",

state == "TAS" ~ "TAS",

state == "WA" ~ "WA",

state == "NT" ~ "NT",

is.na(state) ~ "unclear",

)

)

#unclear have come up as NA but that's fine.

which( colnames(dogads3_test)=="state" )

#to double check

test <- dogads3 %>%

select(sttest <- dogads3 %>%

select(state, state_bin)ate, state_bin)

View(test)

#Let R know correct order

dogads3 <-

dogads3 %>%

mutate(

state_bin = factor(state_bin,

levels = c("QLD","NSW",

"ACT", "VIC",

"TAS", "SA","WA","NT","Unclear")

)

)

#find if any dogs were not given a bin.

dogads3 %>% filter(is.na(age_bin)) %>%

select(state, state_bin)

#plot to ensure that doing the right thing

dogads3 %>% ggplot(aes(state, state_bin)) + geom_point()

#now save the file over the top of the other one (no duplicates AND has age groupings and state groupings)

write_rds(dogads3, "C:/Users/Sofia Costa/OneDrive/Adelaide University Hons/Honours Program/R/finaldogads3.rds")

dogads3[dogads3, sum(dogads3$state_bin), by = dogads3$breed]

rowsum(dogads3, state_bin, reorder = TRUE, na.rm = FALSE)

#################DESCRIPTORS - FREQUENCY FINDING DOGADS3 ################

###total ads per state and aus wide######

adsperstate <- table(dogads3$state)

adsperstate

sum(adsperstate)

table(dogads3$offered_by, dogads3$state)

############breed descriptors############

#list of all the breeds and how many ads per breed

totalperbreed <- table(dogads3$breed)

totalperbreed

write.csv(totalperbreed, file = "totalperbreed.csv", row.names = TRUE)

count(dogads3$state)

#list of unique breeds to ensure no duplicates/spelling errors

numberofbreeds <- count(unique(dogads3$breed))

numberofbreeds

write.csv(numberofbreeds, file = "uniquebreedlist.csv", row.names = TRUE)

#looking at most common breed over 6 months of age

breed_by_age <- table(dogads3$breed, dogads3$age_bin)

write.csv(breed_by_age, file = "breedbyage.csv", row.names = TRUE)

#################breeds per state#######################

#how many times a breed appears per state

breedsperstate1 <- count(dogads3, c("state", "breed"))

#most common breed in individual state

breedsperstate1 <- filter(breedsperstate1, state == "TAS")

#Now arrange by frequency

breedsperstate1 %>%

select (state, breed, freq) %>%

arrange(freq)

breed.crosses <- table(dogads3$breed, dogads3$pure_cross)

breed.crosses

write.csv(breed.crosses, file = "breedcrosses.csv", row.names = TRUE)

#checking why there are designer german sheps etc.

check<- dogads3 %>%

filter(pure_cross=="designer")

write.csv(check, file = "check.csv", row.names = TRUE)

#turns out the weird designers are from purebred parents of diff breeds so is corrct.

###########################################general frequencies####################

######total number of dogs excl. na's#########

sum(dogads3$dogs_available, na.rm = TRUE)

sum(dogads3$number_specified == "2")

#create new dataframe with state and dogs avail (for some reason r wants dob and breed too - Adding missing grouping variables: `dob`, `breedd )

numberofdogsavailable3 <- dogads3 %>%

select(state, dogs_available,state_bin)

#check what this looks like

#replace all na's in the dogs avail column to the average (4.17)

numberofdogsavailable3 <- numberofdogsavailable3 %>% mutate(dogs_available = replace_na(dogs_available, 4.175487))

#check worked

numberofdogsavailable3

#worked!

#sum of dogs available per state

numberofdogsavailable3 %>%

group_by(state) %>%

summarise(sum_enroll = sum(dogs_available))

#sum of dogs available with na's as ave

numberofdogsavailable3 %>%

summarise(sum_enroll = sum(dogs_available))

#####find average size of litters#########

#= create a new dataset with only litters and find ave of that data set

litternumber3 <- dogads3 %>%

select(state, dogs_available)

litternumber3

#this gives us 1735 ads

#how many na's?

sum(is.na(litternumber3))

#177 are na's.

#remove na's

litternumber3 <- na.omit(litternumber3)

litternumber3

#this leaves us with 1558 ads

#remove non-litter sales

litternumber3 <- filter(litternumber3, dogs_available > 1)

litternumber3

#this leaves us with 718 ads with litters (and 840 singleS)

#calculate average

averagelittersize3 <-colMeans(litternumber3$dogs_available, na.rm = TRUE)

averagelittersize3

#this code is equaling 4.175487

averagelittersize3 <-mean(litternumber3$dogs_available, na.rm = TRUE)

averagelittersize3

#this code also equals 4.175487

#########################Microchip descriptors################################

totalmc <- table(dogads3$microchip, dogads3$state)

totalmc

count(dogads3$microchip)

microchip.state <- summarytools::ctable(dogads3$state, dogads3$microchip, prop="r")

microchip.state

#include the percengage by column. i.e. percentage nationally desexed/undesexed

summarytools::ctable(dogads3$state, dogads3$microchip, prop="c")

#########################vaccination descriptors################################

totalvx <- table(dogads3$vaccination)

totalvx

vacc.state <- summarytools::ctable(dogads3$state, dogads3$vaccination, prop="r")

vacc.state

#include the percengage by column. i.e. percentage nationally desexed/undesexed

summarytools::ctable(dogads3$state, dogads3$microchip, prop="c")

##########################vet checked descriptors###############################

totalvet <- table(dogads3$vetcheck) / length(dogads3$vetcheck)

totalvet

pie(table(dogads3$vetcheck))

table(dogads3$microchip)

##########################free dogs descriptors######################

totalfree <- table(dogads3$price ==0 )

totalfree

sum(is.na(dogads3$price))

#include the percentage by row - % in each state

percentfree <- summarytools::ctable(dogads3$state, dogads3$price=="0", prop="r")

percentfree

#include the percengage by column. i.e. percentage nationally

percent.free <- summarytools::ctable(dogads3$state, dogads3$price=="0", prop="c")

percent.free

########################Desexing descriptors##################

totaldsex <-table(dogads3$desexed)

totaldsex

#create dataframe with just the data with dogs over 6 months

six.month.data <- dogads3 %>%

select(state, age_bin, desexed, microchip)

six.month.data

#remove puppies uder 8 weeks

six.month.data <- six.month.data[!(six.month.data$age_bin == "< 8 weeks old"), ]

six.month.data

#emove puppies under 6 months

six.month.data <- six.month.data[!(six.month.data$age_bin == "8 weeks - 6 months"), ]

six.month.data

#look at the percentage by row - % in each state

percentdesexedover6m <- summarytools::ctable(six.month.data$state, six.month.data$desexed, prop="r")

percentdesexedover6m

#animals who are desexed and microchipped

percentdesexedover6mandmicro <-table(six.month.data$desexed, six.month.data$microchip)

percentdesexedover6mandmicro

write.csv(percentdesexedover6mandmicro, file = "desexandchipped.csv", row.names = TRUE)

#######################Pure cross descriptors ################

#total national numbers

totalpurecross <-table(dogads3$pure_cross)

totalpurecross

totalpurecross_age <-table(dogads3$pure_cross, dogads3$age_bin)

totalpurecross_age

percentage <- summarytools::ctable(dogads3$state, dogads3$pure_cross, prop="r")

percentage

#include the percentage by row - % in each state

percentpure <- summarytools::ctable(dogads3$state, dogads3$pure_cross, prop="r")

percentpure

#include the percengage by column. i.e. percentage nationally

pure.cross <- summarytools::ctable(dogads3$state, dogads3$pure_cross, prop="c")

##TABLE WITH PURE_CROSS AND AGES

pure_age <- summarytools::ctable(dogads3$age_bin, dogads3$pure_cross, prop="c")

pure_age

chisq.test(pure_age)

write.csv(pure.cross, file = "pure.cross.csv", row.names = TRUE)

write.csv(pure_age, file = "pure_age.csv", row.names = FALSE)

############################breeder/owner descriptors###################

totalownbreed <-table(dogads3$offered_by)

totalownbreed

table(dogads3$number_specified)

#include the percentage by row - % in each state

percentownbreed <- summarytools::ctable(dogads3$state, dogads3$offered_by, prop="r")

percentownbreed

#include the percengage by column. i.e. percentage nationally

summarytools::ctable(dogads3$state, dogads3$offered_by, prop="c")

#percentage of breeders/owner sales by age of dog

percentownage <- summarytools::ctable(dogads3$age_bin, dogads3$offered_by, prop="c")

percentownage

percentownage2 <- summarytools::ctable(dogads3$age_bin, dogads3$offered_by, prop="r")

percentownage2

#################################price listed########

totalpricelisted <-table(dogads3$price_specified)

totalpricelisted

dogads3 <-

dogads3 %>%

mutate(

price_bin = case_when(

price == 0 ~ "Free",

price < 501 ~ "$1 - $500",

price < 2001 ~ "$501 - $2000",

price > 2000 ~ "$2000+",

is.na(price) ~ "Unknown",

)

)

table(dogads3$price_bin, dogads3$age_bin)

# < 8 weeks old > 7 years 1 - 3 years 3 - 7 years 6 months - 1 year 8 weeks - 6 months Unknown

# $1 - $500 193 9 101 36 61 198 15

# $2000+ 160 0 20 7 19 146 7

# $501 - $2000 216 1 49 19 55 227 21

# Free 7 9 26 26 17 7 7

# Unknown 23 0 12 7 7 21 6

# >

################price frequency##############

summarytools::ctable(dogads3$state, dogads3$price_bin, prop="r")

# ------- ----------- ------------- ------------- -------------- ------------ ----------- ---------------

# price_bin $1 - $500 $2000+ $501 - $2000 Free Unknown Total

# state

# ACT 3 (42.9%) 1 (14.3%) 2 (28.6%) 1 (14.3%) 0 (0.0%) 7 (100.0%)

# na 65 (39.6%) 20 (12.2%) 52 (31.7%) 14 ( 8.5%) 13 (7.9%) 164 (100.0%)

# NSW 211 (36.7%) 113 (19.7%) 190 (33.0%) 32 ( 5.6%) 29 (5.0%) 575 (100.0%)

# NT 12 (60.0%) 0 ( 0.0%) 6 (30.0%) 1 ( 5.0%) 1 (5.0%) 20 (100.0%)

# QLD 173 (34.8%) 106 (21.3%) 181 (36.4%) 25 ( 5.0%) 12 (2.4%) 497 (100.0%)

# SA 29 (29.3%) 25 (25.3%) 39 (39.4%) 3 ( 3.0%) 3 (3.0%) 99 (100.0%)

# TAS 35 (60.3%) 10 (17.2%) 7 (12.1%) 3 ( 5.2%) 3 (5.2%) 58 (100.0%)

# VIC 30 (16.9%) 55 (31.1%) 67 (37.9%) 15 ( 8.5%) 10 (5.6%) 177 (100.0%)

# WA 55 (39.9%) 29 (21.0%) 44 (31.9%) 5 ( 3.6%) 5 (3.6%) 138 (100.0%)

# Total 613 (35.3%) 359 (20.7%) 588 (33.9%) 99 ( 5.7%) 76 (4.4%) 1735 (100.0%)

# ------- ----------- ------------- ------------- -------------- ------------ ----------- ---------------

table(dogads3$price_bin, dogads3$state)

##################age frequncy###########

summarytools::ctable(dogads3$state, dogads3$age_bin, prop="r")

##########################################

totaldognumspecified <-table(dogads3$number_specified)

totaldognumspecified

#####################microchip number##############

totalmcnumber <-table(dogads3$microchip_number)

totalmcnumber

microchip.number.state <- summarytools::ctable(dogads3$state, dogads3$microchip_number, prop="r")

microchip.number.state

#include the percengage by column. i.e. percentage nationally desexed/undesexed

summarytools::ctable(dogads3$state, dogads3$microchip, prop="r")

#########################################ANKC registered desriptors################

totalankc <- table(dogads3$ankc_registered)

totalankc

#include the percengage by row - (percentage by state)

summarytools::ctable(dogads3$state,dogads3$ankc_registered, prop="r")

count(dogads3$breeder_org)

#this column wasnt cleaned. just checking the names of orgs.

table(dogads3$breeder_org, dogads3$org_id)

summarytools::ctable(dogads3$breeder_org,dogads3$org_id, prop="c")

#Going to add new column with ankc and other joined together.

#create new dataset just to be sure we wont cause any issues with previous

#dataset.

dogads3_org <- dogads3

#check how many we will be changing

table(dogads3_org$ankc_registered)

#195 in ankc column

#now we change the value ankc to "yes"

dogads3_org$ankc_registered [dogads3_org$ankc_registered == "ankc"] <- "yes"

#check if the change worked.

table(dogads3_org$ankc_registered)

#195 in the yes column '

#change value other to "yes"

dogads3_org$ankc_registered [dogads3_org$ankc_registered == "other"] <- "yes"

#check if the change worked.

table(dogads3_org$ankc_registered)

#correct.

#change column name to "breeder organisation affiliation)

dogads3_org <- dogads3_org %>%

rename(

"breeder_org_affiliation" = ankc_registered

)

dogads3_org

table(dogads3_org$breeder_org_affiliation, dogads3$org_id)

write.csv(dogads3_org, file = "dogads3_org.csv", row.names = TRUE)

#since ther was some na's in teh wrong spot, have looked at issue in excel

#cleaned this column and got descriptives from excel

# breeder org affiliation yes = 490 ads

#of those 386 inlcuded org ID, and 102 did not.

###########################################State registered descriptors##########

totalstateregister <-table(dogads3$state_registered)

totalstateregister

#include the percengage by row (percentage by state)

summarytools::ctable(dogads3$state, dogads3$state_registered, prop="r")

######PROPORTION TABLES /finding things out per state - rate of compliance table##################

#general how many

summarytools::freq(dogads3$dogs_available)

summarytools

#how many per state

pure.state <- summarytools::ctable(dogads3$state, dogads3$pure_cross, prop="n")

pure.state

summarytools::ctable(dogadsra$breeder_org_affiliation, dogadsra$offered_by, prop="c")

dog.breed.state

#include the percentage by row - i.e. the percentage desexed vs undesexed in

#each state

dog.breed.state <- summarytools::ctable(dogads3$state, dogads3$breed, prop="r")

write.csv(dog.breed.state, file = "uniquebreedlist.csv", row.names = TRUE)

#include the percengage by column. i.e. percentage nationally desexed/undesexed

summarytools::ctable(dogads3$state, dogads3$urgent, prop="c")

dog.price.age <- summarytools::ctable(dogads3$price==500, dogads3$age_bin, prop="r")

dog.price.age

#################making standard % table#####################

#proportion table is divided by total number of ads

#proportion of ads in each state

round(table(dogads3$state)/length(dogads3$state),2)

round(table(dogads3$state, dogads3$microchip)/length(dogads3$microchip),2)

######frequency as proportion to whole amount#######

prop.table(totalmcnumber)

#if i want these numbers rounded out to the third decimal

round(prop.table(totalmcnumber),3)

round(prop.table(dogadsra$breeder_org_affiliation, dogadsra$offered_by),3)

#how many frequencies for this table

margin.table(totalmcnumber)

######how many unique answers in one column###############

numberofbreeds <- count(unique(dogads3$breed))

numberofbreeds

write.csv(numberofbreeds, file = "uniquebreedlist.csv", row.names = TRUE)

sum(numberofbreeds$freq)

####how many cases in the dataframe meet a particular condition#####

sum(finaldogads$microchip == "yes" )

##how many don't equal to means you change the == to !=

sum(finaldogads$microchip != "yes" )

#######to find something that meets two variables###########

##LORA IS THIS CORRECT?????

sum(finaldogads$microchip == "yes" &

finaldogads$vaccination == "yes" )

##check the max of a column, remove na's

max(dogads3$price, na.rm = TRUE)

min(dogads3$price, na.rm = TRUE)

##to see both values at the same time

range(dogads3$price, na.rm = TRUE)

#################price descriptors (value of ads)#######################################

#first multiply all the prices with the animals available.

total_value <- dogads3$dogs_available * dogads3$price

total_value

#then add them all up

sum(total_value, na.rm = TRUE)

#find total sale (ignore all na's) for each state

#start by selecting just the columns we need

pricetotalperstate <- dogads3 %>%

select(state, price, dogs_available)

pricetotalperstate

#now create new column with total of price * dogs avail

pricetotalperstate$totalforthisad <- pricetotalperstate$price * pricetotalperstate$dogs_available

pricetotalperstate

write.csv(pricetotalperstate, file = "pricetotalperstate.csv", row.names = TRUE)

#now add them by state

aggregate(pricetotalperstate$totalforthisad, by=list(pricetotalperstate$state), FUN = sum, na.rm = T)

#find mean (ignore na's) for each state

aggregate (pricetotalperstate$price, by=list(pricetotalperstate$state), FUN = mean, na.rm = T)

table(dogads3$state, dogads3$price)

##########################find total sale if dogs avail na = ave

#replace all na's in the dogs avail column to the average (4.17)

pricetotalperstate <- pricetotalperstate %>% mutate(dogs_available = replace_na(dogs_available, 4.175243))

pricetotalperstate

#make new column so dont disturb old one - this column has the prices per row with the ave if na number of dogs

pricetotalperstate$totalforthisadwithavenumber <- pricetotalperstate$price * pricetotalperstate$dogs_available

pricetotalperstate

aggregate(pricetotalperstate$totalforthisadwithavenumber, by=list(pricetotalperstate$state), FUN = sum, na.rm = T)

aggregate (pricetotalperstate$price, by=list(pricetotalperstate$state), FUN = mean, na.rm = T)

##########find total sale if dogs avail na = ave AND price na = ave price

#= create a new dataset with prices and find ave of that data set

priceave <- dogads3 %>%

select(state, price)

priceave

#how many na's?

sum(is.na(price))

#48 na's

#remove na's

priceave <- na.omit(priceave)

priceave

#calculate average

priceave <- mean(priceave$price, na.rm = TRUE)

priceave

#replace all na's in the price column to the average (1299.582)

pricetotalperstate <- pricetotalperstate %>% mutate(price = replace_na(price, 1299.582))

pricetotalperstate

#make new column so dont disturb old one - this column has the prices per row with the ave if na number of dogs

pricetotalperstate$totalforthisadwithallave <- pricetotalperstate$price * pricetotalperstate$dogs_available

pricetotalperstate

aggregate(pricetotalperstate$totalforthisadwithallave, by=list(pricetotalperstate$state), FUN = sum, na.rm = T)

aggregate (pricetotalperstate$totalforthisadwithallave, by=list(pricetotalperstate$state), FUN = mean, na.rm = T)

##to see min and max at the same time

range(dogads3$price, na.rm = TRUE)

#look at the max price ad

max.price.row <- subset(dogads3,price=="22000")

max.price.row

write.csv(max.price.row, file = "maxpriceads.csv", row.names = TRUE)

#max a seller would make from one ad

range(pricetotalperstate$totalforthisad, na.rm = TRUE)

################stats for prices in age groups#######################

test <- dogads3

tapply(test$price, INDEX = test$age_bin, FUN = mean, na.rm = T )

# frequncy of ads in each bin

agegroups <- count(dogads3$age_bin)

agegroups

#mean of price for each age group

aggregate(dogads3$price, by=list(dogads3$age_bin), FUN = mean, na.rm = T)

#find the median

#mean of price for each age group

aggregate(dogads3$price, by=list(dogads3$age_bin), FUN = median, na.rm = T)

aggregate(dogads3$price, by=list(dogads3$pure_cross), FUN = median, na.rm = T)

#find the mode

#mean of price for each age group

aggregate(dogads3$price, by=list(dogads3$age_bin), FUN = mode, na.rm = T)

###################MORE MICROCHIP ANALYSIS#############

miorochip_age <- table(dogads3$microchip, dogads3$age_bin)

write.csv(miorochip_age, file = "miorochip_age.csv", row.names = TRUE)

tapply(dogadswithfactors$age_bin, dogadswithfactors$microchip, sd, na.rm = T)

dogads3

dogadswithfactors$microchip <- factor(dogadswithfactors$microchip)

dogadswithfactors$age_bin <- factor(dogadswithfactors$age_bin)

dogadswithfactors

table(dogads3$state, dogads3$microchip)

####################QLD COMPLIANCE STATS#########################

#####microchip######

#only compulsary for dogs born after 10th APril 2009

#this was 10 years ago. Are there ads for dogs over 10 years old?

microchip.qld <- dogads3%>%

select(dob, state, age_bin, age, microchip, breed)

microchip.qld

table(microchip.qld$breed == "Pit Bull Terrier", microchip.qld$microchip)

table(dogads3$breed == "Pit Bull Terrier", dogads3$microchip)

#only show qld ads

microchip.qld <- microchip.qld[(microchip.qld$state == "QLD"), ]

microchip.qld

#497 ads- i know there are three ads from dogs older than 2009

#remove ads older than 3650 of age (the amount of days between april 10 2009 and the time

#the final webscrapin was done.)

microchip.qld <- microchip.qld[!(microchip.qld$age > 3650), ]

microchip.qld

#3 ads have been removed. new total is 494

#look at the percentage by row - % in each state

microchipcomp <- summarytools::ctable(microchip.qld$state, microchip.qld$microchip, prop="r")

microchipcomp

microchip.qld_restricted <- dogads3%>%

select(dob, state, age_bin, age, microchip, breed)

microchip.qld_restricted

#there are NA's in this code, but since they're NA for state as well it doesn't

#actually affect the results.

######state regulation######

#state regulated OR ANKC, or Log

#fist we need to remove all working dogs as they are not required to be registered

#start by finding all ads that have the words, rounder, wkc and worker. We will consider

#these working dogs and therefore excepted from state registration

noworkingdogs <- dogads3

noworkingdogs <- grep("rounder", dogads3$description)

noworkingdogs

#found 5

noworkingdogs <-grep("WKC", dogads3$description)

noworkingdogs

#found 5

noworkingdogs <-grep("worker", dogads3$description)

noworkingdogs

#found 5 (2 unique not from other searches)

#now subset just these working dogs and check they are correct

theseareworkingdogs <- dogads3

theseareworkingdogs <- theseareworkingdogs[c(353,370, 1481, 1569, 1573, 318, 324,

332, 1142, 1155, 699, 703), ]

theseareworkingdogs

write.csv(theseareworkingdogs, file = "theseareworkingdogs.csv", row.names = TRUE)

#have checked and these are all working dog ads

#now we need to remove those lines from the noworkingdogs dataframe

noworkingdogs <- dogads3

noworkingdogs <- noworkingdogs[!grepl("rounder", noworkingdogs$description), ]

noworkingdogs

#found 5

noworkingdogs <- noworkingdogs[!grepl("WKC", noworkingdogs$description), ]

noworkingdogs

#found 5

noworkingdogs <-noworkingdogs[!grepl("worker", noworkingdogs$description), ]

noworkingdogs

#found 2 more. There are now 1723 ads, 12 less than original data whcih is correct

write.csv(noworkingdogs, file = "noworkingdogs.csv", row.names = TRUE)

#now get a new dataset. with just the info we need from the no workign dog dataset

registration.qld <- noworkingdogs %>%

select(state, ankc_registered, state_registered, org_id)

registration.qld

#only show qld ads

registration.qld<- registration.qld[(registration.qld$state == "QLD"), ]

registration.qld

#create new column for this dataframe for all ads that are

#either ankc or state registered

registration.qld <- subset(registration.qld , ankc_registered == "ankc" | state_registered == "yes")

registration.qld

#just going to have quick look at data on excel

write.csv(registration.qld, file = "registrationqld.csv", row.names = TRUE)

#correct

#but i need to know how many are registered vs not registered, so subset actually wont help here.

#need ifelse

#lets start by making new column confirming which have ankc numbers in the ad

registration.qld$ankcnumber <- ifelse(registration.qld$ankc_registered == "ankc" &

registration.qld$org_id == "yes", "yes", "no")

registration.qld$qldsupplynumber <- ifelse(registration.qld$ankcnumber == "yes" |

registration.qld$state_registered == "yes", "yes", "no")

registration.qld

#now we look at proportions

summarytools::ctable(registration.qld$state, registration.qld$qldsupplynumber, prop="r")

################desexing###################

#general % desexed - no regulations.

desex.qld <- dogads3[(dogads3$state == "QLD"), ]

desex.qld

#remove puppies uder 8 weeks

desex.qld <- desex.qld[!(desex.qld$age_bin == "< 8 weeks old"), ]

desex.qld

#emove puppies under 6 months

desex.qld <- desex.qld[!(desex.qld$age_bin == "8 weeks - 6 months"), ]

desex.qld

summarytools::ctable(desex.qld$state, desex.qld$desexed, prop="r")

################################WA COMMPLIANCE################

############microchip#############

#all dogs over 3 months of age, no exceptions.

#select just wa

microchip.wa <- dogads3[(dogads3$state == "WA"), ]

microchip.wa

#remove puppies uder 3 months of age (91.25 days)

microchip.wa <- microchip.wa[!(microchip.wa$age < 92), ]

microchip.wa

#look at the percentage by row - % in each state

summarytools::ctable(microchip.wa$state, microchip.wa$microchip, prop="r")

####################SA COMPLIANCE STATS#########################

#####microchip#####

#compulsary for all dogs prior to sale. no exceptions.

#choose just the sections we need

#select just sa

microchip.sa <- dogads3[(dogads3$state == "SA"), ]

microchip.sa

#look at the percentage by row - % in each state

summarytools::ctable(microchip.sa$state, microchip.sa$microchip, prop="r")

# ------- ----------- ------------ ------------ -------------

# microchip no yes Total

# state

# SA 31 (31.3%) 68 (68.7%) 99 (100.0%)

# Total 31 (31.3%) 68 (68.7%) 99 (100.0%)

# ------- ----------- ------------ ------------ -------------

#quick look at what the % is of animals chipped born after July 18.

#remove all dogs older than 268 days.

microchip.sa <- microchip.sa[!(microchip.sa$age > 268 ), ]

microchip.sa

#

# ------- ----------- ------------ ------------ ------------ -------------

# microchip no yes <NA> Total

# state

# SA 23 (29.9%) 54 (70.1%) 0 ( 0.0%) 77 (100.0%)

# <NA> 0 ( 0.0%) 0 ( 0.0%) 7 (100.0%) 7 (100.0%)

# Total 23 (27.4%) 54 (64.3%) 7 ( 8.3%) 84 (100.0%)

# ------- ----------- ------------ ------------ ------------ -------------

>

#now we look at % in ones born before July 2018.

#remove all dogs younger than 268 days.

microchip.sa <- microchip.sa[!(microchip.sa$age < 268 ), ]

microchip.sa

# ------- ----------- ----------- ------------ ------------ -------------

# microchip no yes <NA> Total

# state

# SA 5 (33.3%) 10 (66.7%) 0 ( 0.0%) 15 (100.0%)

# <NA> 0 ( 0.0%) 0 ( 0.0%) 7 (100.0%) 7 (100.0%)

# Total 5 (22.7%) 10 (45.5%) 7 ( 31.8%) 22 (100.0%)

# ------- ----------- ----------- ------------ ------------ -------------

######desexing ########

#all dogs born after july 1 2018 need to be desexed

#within 28 days of aquisition or by 6 months of age.

####note working dogs also exempted!

dogads3(is.na)

#select just sa

desex.sa <- dogads3[(dogads3$state == "SA"), ]

desex.sa

#remove dogs who were born prior to July 1 2018 (will use April 25th a the reference

#date. There are 268 days between July 1 18 and April 25 19. Dogs older than 268 days

#do not require desexing)

#remove all dogs older than 268 days.

desex.sa <- desex.sa[!(desex.sa$age > 268 ), ]

desex.sa

#remove puppies uder 8 weeks

desex.sa <- desex.sa[!(desex.sa$age_bin == "< 8 weeks old"), ]

desex.sa

#emove puppies under 6 months

desex.sa <- desex.sa[!(desex.sa$age_bin == "8 weeks - 6 months"), ]

desex.sa

#look at the percentage by row - % in each state

summarytools::ctable(desex.sa$state, desex.sa$desexed, prop="r")

###########state registered####

#select just sa

register.sa<- dogads3[(dogads3$state == "SA"), ]

register.sa

#include the percengage by row (percentage by state)

summarytools::ctable(register.sa$state, register.sa$state_registered, prop="r")

#compliance

#remove dogs born after July 1 2018

register.sa <- register.sa[!(register.sa$age > 268 ), ]

register.sa

#now remove na's

count(is.na(register.sa$age))

#there are 7 na's and 77 ads with ages.

register.sa<- register.sa %>% filter(!(state == "SA" & age > 268))

table(register.sa$state)

#worked.

summarytools::ctable(register.sa$state, register.sa$state_registered, prop="r")

####################TAS COMPLIANCE STATS#####################

########microchip####

#dogs over 6 months of age must be microchipped, excluidng racing greyhounds (na)

#specified hunting dogs (na) and working dogs (to be removed)

#only show nsw ads

microchip.tas<- noworkingdogs[(noworkingdogs$state == "TAS"), ]

microchip.tas

#remove puppies uder 8 weeks

microchip.tas <- microchip.tas[!(microchip.tas$age_bin == "< 8 weeks old"), ]

microchip.tas

#emove puppies under 6 months

microchip.tas <- microchip.tas[!(microchip.tas$age_bin == "8 weeks - 6 months"), ]

microchip.tas

#look at the percentage by row - % in each state

summarytools::ctable(microchip.tas$state, microchip.tas$microchip, prop="r")

######################NSW COMPLIANCE#################

######################desex#####################

#general % desexed all dogs

desex.nsW <- dogads3[(dogads3$state == "NSW"), ]

desex.nsW

#remove puppies uder 8 weeks

desex.nsW <- desex.nsW[!(desex.nsW$age_bin == "< 8 weeks old"), ]

desex.nsW

#emove puppies under 6 months

desex.nsW <- desex.nsW[!(desex.nsW$age_bin == "8 weeks - 6 months"), ]

desex.nsW

summarytools::ctable(desex.nsW$state, desex.nsW$desexed, prop="r")

###################microchip###########

#all animals must be microchipped prior to sale, exluding working dog from certain areas - but can't be that specific. So will

#use all dogs.

#so need to use the noworkingdogsfile.

#only show nsw ads

microchip.nsw<- dogads3[(dogads3$state == "NSW"), ]

microchip.nsw

#all dogs require microchipping prior to sale

summarytools::ctable(microchip.nsw$state, microchip.nsw$microchip, prop="r")

#############ACT COMPLIANCE###################

################microchipping#######

#all dogs require chip prior to sale

microchip.act <- dogads3[(dogads3$state == "ACT"), ]

microchip.act

summarytools::ctable(microchip.act$state, microchip.act$microchip, prop="r")

##################desex####################

#all dogs must be desexed by 6 months of age. registered racing greyhounds excepted

#(there are none of these in this dataset)

###note there are desex exemptions for dogs born prior to April 2011

desex.act <- dogads3[(dogads3$state == "ACT"), ]

desex.act

#remove puppies uder 8 weeks

desex.qld <- desex.act[!(desex.act$age_bin == "< 8 weeks old"), ]

desex.act

#emove puppies under 6 months

desex.act <- desex.act[!(desex.act$age_bin == "8 weeks - 6 months"), ]

desex.act

summarytools::ctable(desex.act$state, desex.act$desexed, prop="r")

##############VICTORIA COMPLIANCE####################

#####################microchip###############

#dogs require chip prior to sale. no exceptions.

#only show vic ads

microchip.vic<- dogads3[(dogads3$state == "VIC"), ]

microchip.vic

#all dogs require microchipping prior to sale

summarytools::ctable(microchip.vic$state, microchip.vic$microchip, prop="r")

##############desex##################

desex.act <- dogads3[(dogads3$state == "ACT"), ]

desex.act

#remove puppies uder 8 weeks

desex.qld <- desex.act[!(desex.act$age_bin == "< 8 weeks old"), ]

desex.act

#emove puppies under 6 months

desex.act <- desex.act[!(desex.act$age_bin == "8 weeks - 6 months"), ]

desex.act

summarytools::ctable(desex.act$state, desex.act$desexed, prop="r")

#######GENERAL STATS#################

summary <- summary(dogads3)

write.csv(summary, file = "summary.csv", row.names = TRUE)

description <- describe(dogads3)

write.csv(description, file = "description.csv", row.names = TRUE)

sd(dogads3$price, na.rm = T)

var(dogads3$price, na.rm = T)

mean(dogads3$price, na.rm = T)

median(dogads3$price, na.rm = T)

################################FIGURES###############

#############univariate analysis###############

###numerical variable compared to a categorical variable#####

#you might need to turn some variables into categorical variables.

#will use new dataframe so dont mess with old!

dogadswithfactors <- dogads3

#change pure_cross to categorical

dogads3whisker$pure_cross <- factor(dogads3whisker$pure_cross)

#check if worked - yes

dogads3whisker

#boxplot of two variables so we can compare them side by side

plot(price ~ age, data = dogads3)

#boxplot of two variables so we can compare them side by side

plot(pure_cross ~ age, data = dogads3)

#boxplot of two variables so we can compare them side by side

plot(age ~ pure_cross, data = dogads3)

#to check the mean of of price for each pure/cross/designer

tapply(dogadswithfactors$price, dogadswithfactors$pure_cross, mean, na.rm = T)

#now the same but with standard deviation

tapply(dogadswithfactors$price, dogadswithfactors$pure_cross, sd, na.rm = T)

####numerical variable compared to another numerical variable#####

plot(price ~ age, data=dogadswithfactors)

#to add a third variable, i.e. add pure/cross/designer

#notworking, if needed, go back and see utube video - r - explring data part 4 - bivariate summaries

#probably needs to be another numerical value

plot(price ~ age, data=dogadswithfactors, pch = as.integer(pure_cross)

##############CHI ANALYSIS##############

#######prepping data for chi analysis#############

###SEE BELOW TO LOAD CHI ANALYSIS 25/08

#we don't want to include ACT oR NT as these dont have enough data. we also need to combine

#the age bins 3-7 and up for the same reason.

#also want to move all 'unclears' to 'no'

chidataframe <- dogads3

chidataframe

#remove NT

#select just nt

chidataframe<- chidataframe[!(chidataframe$state == "NT"), ]

chidataframe

#this has removed 20 nt rows

#remove act

chidataframe<- chidataframe[!(chidataframe$state == "ACT"), ]

chidataframe

#removed 7 act ads

#just check how many ads in total are in each group/bin

agegroupsforchi <- count(chidataframe$age_bin)

agegroupsforchi

#this tells us that there are 18 ads in 7+ and 3-7 has 95.

#so combined they should have 113 as in the 3+ group

#combine age bins 3-7 and 7 up

chidataframe <-

chidataframe %>%

mutate(

age_bin = case_when(

age < 8*7 ~ "< 8 weeks old",

age < 6 * 4 * 7 ~ "8 weeks - 6 months",

age < 365 ~ "6 months - 1 year",

age < 3 * 365 ~ "1 - 3 years",

age < 7 * 365 ~ "3 + years",

is.na(age) ~ "Unknown",

TRUE ~ "3 + years"

)

)

count(chidataframe$age_bin)

#correct. there is now a new bin of 3+ with 113 ads

chidataframe25_08

##now we need to save this particular dataframe.

#we wil then use excel to remove the unclears to all No's

#and then import that dataframe back into r.

#the variables that need to be moved are:

#desexing, vaccination, ankc registered, and state registerd et.

#no's are recoded to 0, yes is recoded to 1 - FOR THE DEPENDENT VARIABLES ONLY (MICROCHIP AND DESEX)

write.csv(chidataframe, file = "chidataframe.csv", row.names = TRUE)

#load chi analysis

chidataframe25_08 <- read_csv("C:/Users/Sofia Costa/OneDrive/Adelaide University Hons/Honours Program/R/chidataframe25_08.csv")

chidataframe25_08

#now manually add in the price bins from the above load.

chidataframe25_08 <-

chidataframe25_08 %>%

mutate(

price_bin = case_when(

price == 0 ~ "Free",

price < 251 ~ "$1 - $250",

price < 501 ~ "$251 - $500",

price < 1001 ~ "$501 - $1000",

price < 3001 ~ "$1001 - $3000",

price > 3000 ~ "$3001+",

is.na(price) ~ "Unknown",

)

)

###############chi analysis#################

#make a contigency table with the two factors you want

##microchip and pure/cross

#(note for ana, change the == to whichever variable you are testing for the count/row N% tables)

#i.e. chi.microchip.pure <- table(chidataframe25_08$microchip, chidataframe25_08$pure_cross=="cross")

chi.microchip.pure <- table(chidataframe25_08$microchip, chidataframe25_08$pure_cross)

chi.microchip.pure

chisq.test(chi.microchip.pure)

chisq.test(chi.microchip.pure)$expected

##microchip and breed

chi.microchip.breed <- table(chidataframe25_08$microchip, chidataframe25_08$breed)

chi.microchip.breed

chisq.test(chi.microchip.breed)

chisq.test(chi.microchip.breed)$expected

#remove top 10 breeds - oe at a time, repeating the same line of code, with just name of breed changed.

chidataframe25_08breed<- chidataframe25_08breed[!(chidataframe25_08breed$breed == "Jack Russell Terrier"), ]

chidataframe25_08breed

#then re do the table

chi.microchip.breedother <- table(chidataframe25_08breed$microchip, chidataframe25_08breed$breed)

write.csv(chi.microchip.breedother, file = "otherbreeds.csv", row.names = TRUE)

chi.microchip.breedother

chisq.test(chi.microchip.breedother)

chisq.test(chi.microchip.breed)$expected

##microchip and state

chi.microchip.state <- table(chidataframe25_08$microchip, chidataframe25_08$state)

chi.microchip.state

chisq.test(chi.microchip.state)

chisq.test(chi.microchip.state)$expected

##microchip and offered by

chi.microchip.offeredby <- table(chidataframe25_08$microchip, chidataframe25_08$offered_by)

chi.microchip.offeredby

chisq.test(chi.microchip.offeredby)

chisq.test(chi.microchip.offeredby)$expected

##microchip and vaccination

chi.microchip.vx<- table(chidataframe25_08$microchip, chidataframe25_08$vaccination)

chi.microchip.vx

chisq.test(chi.microchip.vx)

chisq.test(chi.microchip.vx)$expected

##microchip and ankc

chi.microchip.ankc<- table(chidataframe25_08$microchip, chidataframe25_08$ankc_registered)

chi.microchip.ankc

chisq.test(chi.microchip.ankc)

chisq.test(chi.microchip.ankc)$expected

##microchip and state registered

chi.microchip.stateregister<- table(chidataframe25_08$microchip, chidataframe25_08$state_registered)

chi.microchip.stateregister

chisq.test(chi.microchip.stateregister)

chisq.test(chi.microchip.stateregister)$expected

##microchip and desexed

chi.microchip.desex<- table(chidataframe25_08$microchip, chidataframe25_08$desexed)

chi.microchip.desex

chisq.test(chi.microchip.desex)

chisq.test(chi.microchip.desex)$expected

##microchip and age

chi.microchip.age <- table(chidataframe25_08$microchip, chidataframe25_08$age_bin)

chi.microchip.age

chisq.test(chi.microchip.age)

chisq.test(chi.microchip.age)$expected

#########BREEDER ID - STATE REGISTERED

#originally did the chi square for ads that had a breeder ID and didnt have breeder ID (yes, no).

#decided need to change to states that require breeder ID and states that do not require breeder ID.

#to do this i need to create new 'bins' for 'state requiring breeder ID' and 'state does not require breeder ID'.

chidataframe25_08

#create bins

chidataframe25_08 <-

chidataframe25_08 %>%

mutate(

ID_bin = case_when(

state == "SA" ~ "required",

state == "NSW" ~ "not required",

state == "QLD" ~ "required",

state == "VIC" ~ "not required",

state == "WA" ~ "not required",

state == "TAS" ~ "not required",

is.na(state) ~ "unclear",

)

)

#unclear have come up as NA but that's fine.

chi.microchip.idrequired<- table(chidataframe25_08$microchip, chidataframe25_08$ID_bin)

chi.microchip.idrequired

chisq.test(chi.microchip.idrequired)

chisq.test(chi.microchip.idrequired)$expected

chidataframe25_08 %>% filter(is.na(ID_bin)) %>%

select(state, state_bin)

##########putting price into bins so we can chi sq test etc.

chidataframe25_08 <-

chidataframe25_08 %>%

mutate(

price_bin = case_when(

price == 0 ~ "Free",

price < 251 ~ "$1 - $250",

price < 501 ~ "$251 - $500",

price < 1001 ~ "$501 - $1000",

price < 3001 ~ "$1001 - $3000",

price > 3000 ~ "$3001+",

is.na(price) ~ "Unknown",

)

)

#to check if worked

chidataframe25_08

write.csv(chidataframe25_08, file = "chidataframe25_08_withpricebin.csv", row.names = TRUE)

#successful

#chi sq test for price

chi.microchip.price<- table(chidataframe25_08$microchip, chidataframe25_08$price_bin)

chi.microchip.price

chisq.test(chi.microchip.price)

chisq.test(chi.microchip.price)$expected

######desexing chi analysis#######

#prepping for chi analysis

#all desexing analysis will need to be for dogs over 6 months of age

#so im going to create a new datafra,e for dgs who are over 6 months but is also

#the same as the othcr chi set.

chidesexdatafame25_08desex6 <- chidataframe25_08

chidesexdatafame25_08desex6

#remove puppies uder 8 weeks

chidesexdatafame25_08desex6 <- chidesexdatafame25_08desex6[!(chidesexdatafame25_08desex6$age_bin == "< 8 weeks old"), ]

chidesexdatafame25_08desex6

#emove puppies under 6 months

chidesexdatafame25_08desex6 <- chidesexdatafame25_08desex6[!(chidesexdatafame25_08desex6$age_bin == "8 weeks - 6 months"), ]

chidesexdatafame25_08desex6

agegroups <- count(chidesexdatafame25_08desex6$age_bin)

agegroups

#actual chi analysis

#desexing and microchip

chi.desex.microchip<- table(chidesexdatafame25_08desex6$desexed, chidesexdatafame25_08desex6$microchip)

chi.desex.microchip

chisq.test(chi.desex.microchip)

chisq.test(chi.desex.microchip)$expected

#desexing and pure/cross

chi.desex.pure<- table(chidesexdatafame25_08desex6$desexed, chidesexdatafame25_08desex6$pure_cross)

chi.desex.pure

chisq.test(chi.desex.pure)

chisq.test(chi.desex.pure)$expected

#desexing and breed

chi.desex.breed<- table(chidesexdatafame25_08desex6$desexed, chidesexdatafame25_08desex6$breed)

chi.desex.breed

chisq.test(chi.desex.breed)

chisq.test(chi.desex.breed)$expected

#remove top 10 breeds - oe at a time, repeating the same line of code, with just name of breed changed.

chidesexdatafame25_08desex6breedother<- chidesexdatafame25_08desex6

chidataframe25_08desexbreed<- chidataframe25_08desexbreed[!(chidataframe25_08desexbreed$breed == "Jack Russell Terrier"), ]

chidataframe25_08desexbreed

#then re do the table

chi.microchip.breeddesexother <- table(chidataframe25_08desexbreed$microchip, chidataframe25_08desexbreed$breed)

write.csv(chi.microchip.breeddesexother, file = "otherdesexbreeds.csv", row.names = TRUE)

chi.microchip.breeddesexother

chisq.test(chi.microchip.breeddesexother)

chisq.test(chi.microchip.breeddesexother)$expected

#desexing and state

chi.desex.state<- table(chidesexdatafame25_08desex6$desexed, chidesexdatafame25_08desex6$state)

chi.desex.state

chisq.test(chi.desex.state)

chisq.test(chi.desex.state)$expected

#desexing and BREEDER ID (STATE REGISTERED)

chi.desex.stateid<- table(chidesexdatafame25_08desex6$desexed, chidesexdatafame25_08desex6$state_registered)

chi.desex.stateid

chisq.test(chi.desex.stateid)

chisq.test(chi.desex.stateid)$expected

#desexing and offered by

chi.desex.offeredby<- table(chidesexdatafame25_08desex6$desexed, chidesexdatafame25_08desex6$offered_by)

chi.desex.offeredby

chisq.test(chi.desex.offeredby)

chisq.test(chi.desex.offeredby)$expected

#desexing and vx

chi.desex.vx<- table(chidesexdatafame25_08desex6$desexed, chidesexdatafame25_08desex6$vaccination)

chi.desex.vx

chisq.test(chi.desex.vx)

chisq.test(chi.desex.vx)$expected

#desexing and ankc registered

chi.desex.ankc<- table(chidesexdatafame25_08desex6$desexed, chidesexdatafame25_08desex6$ankc_registered)

chi.desex.ankc

chisq.test(chi.desex.ankc)

chisq.test(chi.desex.ankc)$expected

#desexing and state registered

chi.desex.stateregister<- table(chidesexdatafame25_08desex6$desexed, chidesexdatafame25_08desex6$state_registered)

chi.desex.stateregister

chisq.test(chi.desex.stateregister)

chisq.test(chi.desex.stateregister)$expected

#desexing and ID_BIN

chi.desex.idbin<- table(chidesexdatafame25_08desex6$desexed, chidesexdatafame25_08desex6$ID_bin)

chi.desex.idbin

chisq.test(chi.desex.idbin)

chisq.test(chi.desex.idbin)$expected

####desexing and price bin

chi.desex.price<- table(chidesexdatafame25_08desex6$desexed, chidesexdatafame25_08desex6$price_bin)

chi.desex.price

chisq.test(chi.desex.price)

chisq.test(chi.desex.price)$expected

####desexing and age bin

chi.desex.age<- table(chidesexdatafame25_08desex6$desexed, chidesexdatafame25_08desex6$age_bin)

chi.desex.age

chisq.test(chi.desex.age)

chisq.test(chi.desex.age)$expected

###########################RO FOR MICROCHIP#####################################################

#need to turn all independant variables into categorical variables.

#pure/cross

#breed

#state

#offered by

#vaccinatin

#ankc registered

#state registered

#will use new dataframe so dont mess with old

dogadsor <- chidataframe25_08

dogadsor

#change pure_cross to categorical

dogadsor$pure_cross <- factor(dogadsor$pure_cross)

#check if worked - yes

dogadsor

#change breed to categorical

dogadsor$breed <- factor(dogadsor$breed)

#check if worked - yes

dogadsor

#change state to categorical

dogadsor$state <- factor(dogadsor$state)

#check if worked - yes

dogadsor

#change offered by to categorical

dogadsor$offered_by <- factor(dogadsor$offered_by)

#check if worked - yes

dogadsor

#change vaccination to categorical

dogadsor$vaccination <- factor(dogadsor$vaccination)

#check if worked - yes

dogadsor

#change ank registered to categorical

dogadsor$ankc_registered <- factor(dogadsor$ankc_registered)

#check if worked - yes

dogadsor

#change state registered to categorical

dogadsor$state_registered <- factor(dogadsor$state_registered)

#check if worked - yes

dogadsor

#change state ID to categorical

dogadsor$price_bin <- factor(dogadsor$ID_bin)

#check if worked - yes

dogadsor

#change age bin to categorical

dogadsor$price_bin <- factor(dogadsor$price_bin)

#check if worked - yes

dogadsor

#Now we save this dataframe so we have easy access to it in the future

write.csv(dogadsor, file = "dogadsor.csv", row.names = TRUE)

#loaddogsor - chidataframe (no act.nt etc) but with independent variables as factors

dogadsor <- read_csv("C:/Users/Sofia Costa/OneDrive/Adelaide University Hons/Honours Program/R/dogadsor.csv")

#######create the model for pure_cross

modelpure <- glm(microchip ~ pure_cross, family = binomial, data = dogadsor)

summary(modelpure)

#Coefficients:

#Estimate Std. Error z value Pr(>|z|)

#(Intercept) 0.35918 0.09238 3.888 0.000101 ***

# pure_crossdesigner 1.96142 0.32913 5.959 2.53e-09 ***

# pure_crosspure 0.72710 0.11598 6.269 3.63e-10 ***

# pure_crossunknown -0.62155 0.43065 -1.443 0.148939

#---

#Signif. codes: 0 ‘***’ 0.001 ‘**’ 0.01 ‘*’ 0.05 ‘.’ 0.1 ‘ ’ 1

#(Dispersion parameter for binomial family taken to be 1)

#Null deviance: 2056.4 on 1707 degrees of freedom

#Residual deviance: 1979.1 on 1704 degrees of freedom

#AIC: 1987.1

# get the confidence interval

pure.crossci <- confint(modelpure)

round(exp(cbind(model1$coef, pure.crossci)), digits = 2)

#2.5 % 97.5 %

# (Intercept) 1.43 1.20 1.72

#pure_crossdesigner 7.11(or) 3.89 14.32

#pure_crosspure 2.07 1.65 2.60

#pure_crossunknown 0.54 0.23 1.25

modelpureRR<-odds_to_rr(model1)

format(round(modelpureRR,2),nsmall=2)

# RR lower.ci upper.ci

#(Intercept) 1.10 1.05 1.14

#pure_crossdesigner 1.33 1.27 1.37

#pure_crosspure 1.18 1.13 1.22

#pure_crossunknown 0.80 0.50 1.06

######################STATE

#reference category is 'unknown' if r chooses, so need to change

table(dogadsor$state)

#note na comes first, r puts the first category (alphabetically) as the auto reference

dogadsor$state <- relevel(dogadsor$state, ref= "VIC")

table(dogadsor$state)

#vic now comes first and therefore is the reference.

#########create the model for state

modelstate <- glm(microchip ~ state, family = binomial, data = dogadsor)

summary(modelstate)

#Coefficients:

#Estimate Std. Error z value Pr(>|z|)

#(Intercept) 2.5349 0.2881 8.798 < 2e-16 ***

# statena -2.7553 0.3282 -8.396 < 2e-16 ***

# stateNSW -1.5905 0.3027 -5.254 1.49e-07 ***

# stateQLD -1.3681 0.3068 -4.459 8.24e-06 ***

# stateSA -1.7494 0.3605 -4.852 1.22e-06 ***

# stateTAS -2.5349 0.3899 -6.502 7.91e-11 ***

# stateWA -2.0318 0.3375 -6.021 1.73e-09 ***

# ---

# Signif. codes: 0 ‘***’ 0.001 ‘**’ 0.01 ‘*’ 0.05 ‘.’ 0.1 ‘ ’ 1

#(Dispersion parameter for binomial family taken to be 1)

#Null deviance: 2056.4 on 1707 degrees of freedom

#Residual deviance: 1931.3 on 1701 degrees of freedom

#AIC: 1945.3

# get the confidence interval

stateci <- confint(modelstate)

round(exp(cbind(modelstate$coef, stateci)), digits = 2)

# (OR and 95%OR) 2.5 % 97.5 %

#(Intercept) 12.62 7.47 23.34

#statena 0.06 0.03 0.12

#stateNSW 0.20 0.11 0.36

#stateQLD 0.25 0.13 0.45

#stateSA 0.17 0.08 0.35

#stateTAS 0.08 0.04 0.17

#stateWA 0.13 0.07 0.25

modelstateRR<-odds_to_rr(modelstate)

format(round(modelstateRR,2),nsmall=2)

# RR lower.ci upper.ci

#(Intercept) 1.36 1.34 1.38

#statena 0.19 0.10 0.31

#stateNSW 0.47 0.29 0.66

#stateQLD 0.54 0.35 0.74

#stateSA 0.42 0.24 0.65

#stateTAS 0.23 0.11 0.41

#stateWA 0.34 0.19 0.53

#########create the model for breeder ID number included in ad (state registered)

#this includes ALL STATES even those without id.

modelstateregister <- glm(microchip ~ state_registered, family = binomial, data = dogadsor)

summary(modelstateregister)

# call:

# glm(formula = microchip ~ state_registered, family = binomial,

# data = dogadsor)

#

# Deviance Residuals:

# Min 1Q Median 3Q Max

# -2.0407 -1.4783 0.9038 0.9038 0.9038

#

# Coefficients:

# Estimate Std. Error z value Pr(>|z|)

# (Intercept) 0.68420 0.05789 11.820 < 2e-16 ***

# state_registeredyes 1.26481 0.16789 7.534 4.93e-14 ***

# ---

# Signif. codes: 0 ‘***’ 0.001 ‘**’ 0.01 ‘*’ 0.05 ‘.’ 0.1 ‘ ’ 1

#

# (Dispersion parameter for binomial family taken to be 1)

#

# Null deviance: 2056.4 on 1707 degrees of freedom

# Residual deviance: 1985.8 on 1706 degrees of freedom

# AIC: 1989.8

#

# Number of Fisher Scoring iterations: 4

# get the confidence interval

stateregisterci <- confint(modelstateregister)

round(exp(cbind(modelstateregister$coef, stateregisterci)), digits = 2)

# 2.5 % 97.5 %

# (Intercept) 1.98 1.77 2.22

# state_registeredyes 3.54 2.57 4.98

modelstateregisterRR<-odds_to_rr(modelstateregister)

format(round(modelstateregisterRR,2),nsmall=2)

# RR lower.ci upper.ci

# (Intercept) 1.17 1.14 1.19

# state_registeredyes 1.26 1.22 1.30

####VACCINATION

#########create the model for vaccination

modelvx <- glm(microchip ~ vaccination, family = binomial, data = dogadsor)

summary(modelvx)

#Coefficients:

#Estimate Std. Error z value Pr(>|z|)

#(Intercept) -1.5848 0.1276 -12.42 <2e-16 ***

#vaccinationyes 3.7249 0.1569 23.74 <2e-16 ***

# ---

# Signif. codes: 0 ‘***’ 0.001 ‘**’ 0.01 ‘*’ 0.05 ‘.’ 0.1 ‘ ’ 1

#(Dispersion parameter for binomial family taken to be 1)

#Null deviance: 2056.4 on 1707 degrees of freedom

#Residual deviance: 1253.5 on 1706 degrees of freedom

#AIC: 1257.5

# get the confidence interval

vxci <- confint(modelvx)

round(exp(cbind(modelvx$coef, vxci)), digits = 2)

# 2.5 % 97.5 %

#(Intercept) 0.20 0.16 0.26

#vaccinationyes 41.47 30.66 56.75

modelvxRR<-odds_to_rr(modelvx)

format(round(modelvxRR,2),nsmall=2)

# RR lower.ci upper.ci

#(Intercept) 0.47 0.39 0.55

#vaccinationyes 1.39 1.39 1.40

#####BREED##

#make American Staffordshire Terrier the reference breed

dogadsor$breed <- relevel(dogadsor$breed, ref= "American Staffordshire Terrier")

table(dogadsor$breed)

#confirmed

modelbreed <- glm(microchip ~ breed, family = binomial, data = dogadsor)

summary(modelbreed)

# Coefficients:

# Estimate Std. Error z value Pr(>|z|)

# (Intercept) 7.167e-01 1.882e-01 3.807 0.000141 ***

# breedAiredale Terrier 1.685e+01 3.956e+03 0.004 0.996602

# breedAkita 3.819e-01 1.170e+00 0.326 0.744080

# breedAlaskan Husky 1.685e+01 3.956e+03 0.004 0.996602

# breedAlaskan Malamute 1.685e+01 2.797e+03 0.006 0.995194

# breedAmerican Bulldog -6.113e-01 4.965e-01 -1.231 0.218262

# breedAmerican Bully 1.996e-01 8.576e-01 0.233 0.815945

# breedAustralian Bulldog 1.768e+00 1.058e+00 1.672 0.094576 .

# breedAustralian Cattle Dog -6.521e-01 3.163e-01 -2.062 0.039205 *

# breedAustralian Koolie -2.059e-01 7.542e-01 -0.273 0.784890

# breedAustralian Shepherd 1.685e+01 2.797e+03 0.006 0.995194

# breedAustralian Silky Terrier -1.828e+01 2.797e+03 -0.007 0.994785

# breedAustralian Stumpy Tail Cattle Dog 1.685e+01 2.797e+03 0.006 0.995194

# breedAustralian Terrier 1.685e+01 3.956e+03 0.004 0.996602

# breedBandog -9.398e-01 6.967e-01 -1.349 0.177370

# breedBasset Hound -1.828e+01 3.956e+03 -0.005 0.996313

# breedBeagle -5.344e-01 6.341e-01 -0.843 0.399409

# breedBeaglier 1.685e+01 2.797e+03 0.006 0.995194

# breedBelgian Shepherd -7.167e-01 1.427e+00 -0.502 0.615431

# breedBichon 1.685e+01 3.956e+03 0.004 0.996602

# breedBichoodle 1.685e+01 3.956e+03 0.004 0.996602

# breedBorder Collie -1.906e-01 3.108e-01 -0.613 0.539802

# breedBoston Terrier 1.685e+01 1.978e+03 0.009 0.993204

# breedBoxer 5.361e-01 8.236e-01 0.651 0.515101

# breedBritish Bulldog 9.415e-01 5.771e-01 1.631 0.102787

# breedBull Arab -4.935e-01 3.542e-01 -1.393 0.163472

# breedBull Terrier -7.167e-01 1.427e+00 -0.502 0.615431

# breedBulldog 1.685e+01 3.956e+03 0.004 0.996602

# breedBullmastiff -5.831e-01 5.507e-01 -1.059 0.289656

# breedCane Corso -7.167e-01 1.018e+00 -0.704 0.481242

# breedCavachon 1.685e+01 3.956e+03 0.004 0.996602

# breedCavalier King Charles Spaniel 6.183e-01 5.367e-01 1.152 0.249305

# breedCavashoo 1.685e+01 3.956e+03 0.004 0.996602

# breedCavocker 1.685e+01 3.956e+03 0.004 0.996602

# breedCavoodle 1.586e+00 5.572e-01 2.846 0.004422 **

# breedChihuahua 4.689e-01 3.926e-01 1.195 0.232281

# breedChinese Crested Dog 1.685e+01 1.978e+03 0.009 0.993204

# breedChion 1.685e+01 3.956e+03 0.004 0.996602

# breedChiweenie 1.685e+01 3.956e+03 0.004 0.996602

# breedCocker Spaniel 3.819e-01 1.170e+00 0.326 0.744080

# breedCorgi -1.828e+01 3.956e+03 -0.005 0.996313

# breedDachshund 3.819e-01 8.379e-01 0.456 0.648523

# breedDalmatian 1.685e+01 2.284e+03 0.007 0.994114

# breedDingo 1.685e+01 3.956e+03 0.004 0.996602

# breedDobermann -7.167e-01 1.018e+00 -0.704 0.481242

# breedDogue de Bordeaux 1.306e-01 7.153e-01 0.183 0.855101

# breedEnglish Cocker Spaniel -1.828e+01 3.956e+03 -0.005 0.996313

# breedEnglish Mastiff -1.828e+01 3.956e+03 -0.005 0.996313

# breedEnglish Pointer 1.685e+01 1.978e+03 0.009 0.993204

# breedEnglish Springer Spaniel -7.167e-01 8.379e-01 -0.855 0.392380

# breedFox Terrier -4.935e-01 6.967e-01 -0.708 0.478725

# breedFrench Bulldog 1.751e+00 3.794e-01 4.617 3.9e-06 ***

# breedFrenchton 1.685e+01 3.956e+03 0.004 0.996602

# breedFrug 1.685e+01 1.978e+03 0.009 0.993204

# breedGerman Shepherd 2.076e-01 3.022e-01 0.687 0.492112

# breedGerman Shorthaired Pointer -2.059e-01 7.542e-01 -0.273 0.784890

# breedGerman Spitz<U+00A0> 1.685e+01 2.797e+03 0.006 0.995194

# breedGoldador 1.685e+01 3.956e+03 0.004 0.996602

# breedGolden Retriever 6.696e-01 1.134e+00 0.591 0.554782

# breedGreat Dane -9.680e-01 5.380e-01 -1.799 0.071962 .

# breedGreyhound -4.935e-01 6.967e-01 -0.708 0.478725

# breedGriffon Bruxellois 1.685e+01 3.956e+03 0.004 0.996602

# breedGroodle 1.075e+00 1.096e+00 0.981 0.326814

# breedHarrier 1.685e+01 2.797e+03 0.006 0.995194

# breedHavanese 1.685e+01 3.956e+03 0.004 0.996602

# breedHound -1.828e+01 2.797e+03 -0.007 0.994785

# breedHusky -2.353e-02 6.407e-01 -0.037 0.970701

# breedIrish Wolfhound 4.873e-01 6.847e-01 0.712 0.476636

# breedJack Russell Terrier 4.408e-01 3.936e-01 1.120 0.262771

# breedJapanese Chin 1.685e+01 3.956e+03 0.004 0.996602

# breedJohnson Bulldog -1.410e+00 1.239e+00 -1.138 0.255222

# breedJug -1.828e+01 3.956e+03 -0.005 0.996313

# breedKangal Shepherd Dog -1.828e+01 3.956e+03 -0.005 0.996313

# breedKelpie -5.590e-01 2.840e-01 -1.968 0.049020 *

# breedLabradogue 1.685e+01 3.956e+03 0.004 0.996602

# breedLabradoodle 5.361e-01 8.236e-01 0.651 0.515101

# breedLabrador Retriever 6.696e-01 4.378e-01 1.529 0.126158

# breedLhasa Apso 1.685e+01 3.956e+03 0.004 0.996602

# breedMalchi 1.685e+01 3.956e+03 0.004 0.996602

# breedMalinois 3.819e-01 1.170e+00 0.326 0.744080

# breedMaltese 7.004e-01 4.367e-01 1.604 0.108785

# breedMaltese Shih Tzu 9.425e-02 6.297e-01 0.150 0.881022

# breedMaremma 1.685e+01 3.956e+03 0.004 0.996602

# breedMaremma Sheepdog -5.989e-01 5.211e-01 -1.149 0.250440

# breedMastiff -6.214e-01 4.758e-01 -1.306 0.191534

# breedMiniature Australian Bulldog 1.685e+01 3.956e+03 0.004 0.996602

# breedMiniature Bull Terrier 1.685e+01 3.956e+03 0.004 0.996602

# breedMiniature Cavoodle 1.685e+01 3.956e+03 0.004 0.996602

# breedMiniature Dachshund 1.685e+01 8.435e+02 0.020 0.984062

# breedMiniature Fox Terrier<U+00A0> 4.465e-01 5.458e-01 0.818 0.413379

# breedMiniature Jack Russell 1.685e+01 3.956e+03 0.004 0.996602

# breedMiniature Labradoodle 1.685e+01 3.956e+03 0.004 0.996602

# breedMiniature Maltese -1.828e+01 3.956e+03 -0.005 0.996313

# breedMiniature Pinscher 1.685e+01 2.797e+03 0.006 0.995194

# breedMiniature Poodle 6.696e-01 1.134e+00 0.591 0.554782

# breedMiniature Schnauzer 1.685e+01 2.284e+03 0.007 0.994114

# breedMiniature Spoodle 1.685e+01 2.797e+03 0.006 0.995194

# breedMoodle 1.685e+01 1.319e+03 0.013 0.989806

# breedMurray River Retriever 1.685e+01 3.956e+03 0.004 0.996602

# breedNeapolitan Mastiff 1.685e+01 1.495e+03 0.011 0.991009

# breedNewfoundland 1.685e+01 3.956e+03 0.004 0.996602

# breedPapillon -1.828e+01 3.956e+03 -0.005 0.996313

# breedPekingese 1.685e+01 3.956e+03 0.004 0.996602

# breedPembroke Welsh Corgi<U+00A0> 1.685e+01 2.797e+03 0.006 0.995194

# breedPinoodle 1.685e+01 3.956e+03 0.004 0.996602

# breedPit Bull Terrier -1.073e+00 5.275e-01 -2.035 0.041886 *

# breedPomchi 1.685e+01 2.284e+03 0.007 0.994114

# breedPomeranian 9.425e-02 4.647e-01 0.203 0.839289

# breedPoochin 1.685e+01 3.956e+03 0.004 0.996602

# breedPoodle 1.685e+01 1.251e+03 0.013 0.989254

# breedPug 4.689e-01 3.926e-01 1.195 0.232281

# breedPugalier 1.685e+01 2.284e+03 0.007 0.994114

# breedPuggle 1.685e+01 2.797e+03 0.006 0.995194

# breedPuli 1.685e+01 3.956e+03 0.004 0.996602

# breedRhodesian Ridgeback 1.996e-01 6.208e-01 0.322 0.747814

# breedRottweiler -4.030e-01 3.557e-01 -1.133 0.257215

# breedRough Collie -2.353e-02 1.239e+00 -0.019 0.984849

# breedSamoyed 1.685e+01 2.797e+03 0.006 0.995194

# breedSchnauzer 1.685e+01 3.956e+03 0.004 0.996602

# breedSchnoodle 1.685e+01 3.956e+03 0.004 0.996602

# breedShar Pei -7.167e-01 6.599e-01 -1.086 0.277445

# breedShepsky 1.685e+01 3.956e+03 0.004 0.996602

# breedShih Tzu 1.685e+01 1.769e+03 0.010 0.992402

# breedSiberian Husky -5.344e-01 6.341e-01 -0.843 0.399409

# breedSmithfield 1.685e+01 2.797e+03 0.006 0.995194

# breedSouthern Bulldog 1.685e+01 3.956e+03 0.004 0.996602

# breedSpoodle 6.696e-01 1.134e+00 0.591 0.554782

# breedSt. Bernard 1.685e+01 3.956e+03 0.004 0.996602

# breedStaffordshire Bull Terrier 3.138e-03 2.750e-01 0.011 0.990896

# breedStaghound -1.122e+00 9.321e-01 -1.204 0.228623

# breedSwiss Shepherd -2.353e-02 1.239e+00 -0.019 0.984849

# breedTenterfield Terrier 1.685e+01 2.797e+03 0.006 0.995194

# breedTerrier 1.685e+01 3.956e+03 0.004 0.996602

# breedToy Cavoodle 1.685e+01 3.956e+03 0.004 0.996602

# breedToy Moodle 1.685e+01 2.797e+03 0.006 0.995194

# breedToy Pomeranian 1.685e+01 3.956e+03 0.004 0.996602

# breedToy Poodle 1.075e+00 5.719e-01 1.880 0.060143 .

# breedUnknown -7.167e-01 4.222e-01 -1.697 0.089643 .

# breedWeimarhund 1.685e+01 3.956e+03 0.004 0.996602

# breedWest Highland White Terrier 1.685e+01 2.797e+03 0.006 0.995194

# breedWhippet -7.167e-01 1.018e+00 -0.704 0.481242

# breedYorkshire Terrier -1.828e+01 3.956e+03 -0.005 0.996313

# ---

# Signif. codes: 0 ‘***’ 0.001 ‘**’ 0.01 ‘*’ 0.05 ‘.’ 0.1 ‘ ’ 1

#

# (Dispersion parameter for binomial family taken to be 1)

#

# Null deviance: 2056.4 on 1707 degrees of freedom

# Residual deviance: 1775.8 on 1566 degrees of freedom

# AIC: 2059.8

# get the confidence interval

breedci <- confint(modelbreed)

round(exp(cbind(modelbreed$coef, breedci)), digits = 2)

# Waiting for profiling to be done...

# There were 50 or more warnings (use warnings() to see the first 50)

# > round(exp(cbind(modelbreed$coef, breedci)), digits = 2)

# 2.5 % 97.5 %

# (Intercept) 2.05 1.43 2.990000e+00

# breedAiredale Terrier 20777699.05 0.00 NA

# breedAkita 1.47 0.18 3.010000e+01

# breedAlaskan Husky 20777699.05 0.00 NA

# breedAlaskan Malamute 20777699.06 0.00 NA

# breedAmerican Bulldog 0.54 0.20 1.460000e+00

# breedAmerican Bully 1.22 0.25 8.770000e+00

# breedAustralian Bulldog 5.86 1.10 1.085400e+02

# breedAustralian Cattle Dog 0.52 0.28 9.700000e-01

# breedAustralian Koolie 0.81 0.19 4.120000e+00

# breedAustralian Shepherd 20777699.06 0.00 NA

# breedAustralian Silky Terrier 0.00 NA 5.416237e+182

# breedAustralian Stumpy Tail Cattle Dog 20777699.05 0.00 NA

# breedAustralian Terrier 20777699.05 0.00 NA

# breedBandog 0.39 0.09 1.550000e+00

# breedBasset Hound 0.00 NA Inf

# breedBeagle 0.59 0.17 2.140000e+00

# breedBeaglier 20777699.04 0.00 NA

# breedBelgian Shepherd 0.49 0.02 1.255000e+01

# breedBichon 20777699.04 0.00 NA

# breedBichoodle 20777699.04 0.00 NA

# breedBorder Collie 0.83 0.45 1.530000e+00

# breedBoston Terrier 20777699.04 0.00 NA

# breedBoxer 1.71 0.39 1.181000e+01

# breedBritish Bulldog 2.56 0.91 9.210000e+00

# breedBull Arab 0.61 0.30 1.230000e+00

# breedBull Terrier 0.49 0.02 1.255000e+01

# breedBulldog 20777699.05 0.00 NA

# breedBullmastiff 0.56 0.19 1.690000e+00

# breedCane Corso 0.49 0.06 4.180000e+00

# breedCavachon 20777699.06 0.00 NA

# breedCavalier King Charles Spaniel 1.86 0.69 5.900000e+00

# breedCavashoo 20777699.04 0.00 NA

# breedCavocker 20777699.05 0.00 NA

# breedCavoodle 4.88 1.82 1.705000e+01

# breedChihuahua 1.60 0.76 3.580000e+00

# breedChinese Crested Dog 20777699.06 0.00 NA

# breedChion 20777699.05 0.00 NA

# breedChiweenie 20777699.05 0.00 NA

# breedCocker Spaniel 1.47 0.18 3.010000e+01

# breedCorgi 0.00 NA Inf

# breedDachshund 1.47 0.32 1.029000e+01

# breedDalmatian 20777699.06 0.00 NA

# breedDingo 20777699.05 0.00 NA

# breedDobermann 0.49 0.06 4.180000e+00

# breedDogue de Bordeaux 1.14 0.30 5.490000e+00

# breedEnglish Cocker Spaniel 0.00 NA Inf

# breedEnglish Mastiff 0.00 NA Inf

# breedEnglish Pointer 20777699.04 0.00 NA

# breedEnglish Springer Spaniel 0.49 0.09 2.740000e+00

# breedFox Terrier 0.61 0.15 2.580000e+00

# breedFrench Bulldog 5.76 2.84 1.274000e+01

# breedFrenchton 20777699.05 0.00 NA

# breedFrug 20777699.04 0.00 NA

# breedGerman Shepherd 1.23 0.68 2.240000e+00

# breedGerman Shorthaired Pointer 0.81 0.19 4.120000e+00

# breedGerman Spitz<U+00A0> 20777699.04 0.00 NA

# breedGoldador 20777699.05 0.00 NA

# breedGolden Retriever 1.95 0.28 3.885000e+01

# breedGreat Dane 0.38 0.13 1.090000e+00

# breedGreyhound 0.61 0.15 2.580000e+00

# breedGriffon Bruxellois 20777699.04 0.00 NA

# breedGroodle 2.93 0.48 5.628000e+01

# breedHarrier 20777699.05 0.00 NA

# breedHavanese 20777699.06 0.00 NA

# breedHound 0.00 NA 5.416237e+182

# breedHusky 0.98 0.29 3.830000e+00

# breedIrish Wolfhound 1.63 0.47 7.540000e+00

# breedJack Russell Terrier 1.55 0.74 3.480000e+00

# breedJapanese Chin 20777699.05 0.00 NA

# breedJohnson Bulldog 0.24 0.01 2.620000e+00

# breedJug 0.00 NA Inf

# breedKangal Shepherd Dog 0.00 NA Inf

# breedKelpie 0.57 0.33 1.000000e+00

# breedLabradogue 20777699.04 0.00 NA

# breedLabradoodle 1.71 0.39 1.181000e+01

# breedLabrador Retriever 1.95 0.86 4.880000e+00

# breedLhasa Apso 20777699.04 0.00 NA

# breedMalchi 20777699.05 0.00 NA

# breedMalinois 1.47 0.18 3.010000e+01

# breedMaltese 2.01 0.89 5.030000e+00

# breedMaltese Shih Tzu 1.10 0.34 4.240000e+00

# breedMaremma 20777699.05 0.00 NA

# breedMaremma Sheepdog 0.55 0.20 1.560000e+00

# breedMastiff 0.54 0.21 1.380000e+00

# breedMiniature Australian Bulldog 20777699.06 0.00 NA

# breedMiniature Bull Terrier 20777699.05 0.00 NA

# breedMiniature Cavoodle 20777699.04 0.00 NA

# breedMiniature Dachshund 20777699.05 0.00 NA

# breedMiniature Fox Terrier<U+00A0> 1.56 0.57 5.040000e+00

# breedMiniature Jack Russell 20777699.05 0.00 NA

# breedMiniature Labradoodle 20777699.05 0.00 NA

# breedMiniature Maltese 0.00 NA Inf

# breedMiniature Pinscher 20777699.04 0.00 NA

# breedMiniature Poodle 1.95 0.28 3.885000e+01

# breedMiniature Schnauzer 20777699.06 0.00 NA

# breedMiniature Spoodle 20777699.05 0.00 NA

# breedMoodle 20777699.04 0.00 NA

# breedMurray River Retriever 20777699.04 0.00 NA

# breedNeapolitan Mastiff 20777699.05 0.00 NA

# breedNewfoundland 20777699.04 0.00 NA

# breedPapillon 0.00 NA Inf

# breedPekingese 20777699.05 0.00 NA

# breedPembroke Welsh Corgi<U+00A0> 20777699.06 0.00 NA

# breedPinoodle 20777699.05 0.00 NA

# breedPit Bull Terrier 0.34 0.12 9.500000e-01

# breedPomchi 20777699.04 0.00 NA

# breedPomeranian 1.10 0.45 2.870000e+00

# breedPoochin 20777699.04 0.00 NA

# breedPoodle 20777699.04 0.00 NA

# breedPug 1.60 0.76 3.580000e+00

# breedPugalier 20777699.06 0.00 NA

# breedPuggle 20777699.04 0.00 NA

# breedPuli 20777699.04 0.00 NA

# breedRhodesian Ridgeback 1.22 0.38 4.660000e+00

# breedRottweiler 0.67 0.33 1.350000e+00

# breedRough Collie 0.98 0.09 2.137000e+01

# breedSamoyed 20777699.04 0.00 NA

# breedSchnauzer 20777699.06 0.00 NA

# breedSchnoodle 20777699.06 0.00 NA

# breedShar Pei 0.49 0.13 1.840000e+00

# breedShepsky 20777699.05 0.00 NA

# breedShih Tzu 20777699.05 0.00 NA

# breedSiberian Husky 0.59 0.17 2.140000e+00

# breedSmithfield 20777699.04 0.00 NA

# breedSouthern Bulldog 20777699.04 0.00 NA

# breedSpoodle 1.95 0.28 3.885000e+01

# breedSt. Bernard 20777699.05 0.00 NA

# breedStaffordshire Bull Terrier 1.00 0.59 1.720000e+00

# breedStaghound 0.33 0.04 2.040000e+00

# breedSwiss Shepherd 0.98 0.09 2.137000e+01

# breedTenterfield Terrier 20777699.05 0.00 NA

# breedTerrier 20777699.04 0.00 NA

# breedToy Cavoodle 20777699.05 0.00 NA

# breedToy Moodle 20777699.06 0.00 NA

# breedToy Pomeranian 20777699.05 0.00 NA

# breedToy Poodle 2.93 1.05 1.045000e+01

# breedUnknown 0.49 0.21 1.120000e+00

# breedWeimarhund 20777699.04 0.00 NA

# breedWest Highland White Terrier 20777699.06 0.00 NA

# breedWhippet 0.49 0.06 4.180000e+00

# breedYorkshire Terrier 0.00 NA Inf

modelbreedRR<-odds_to_rr(modelbreed)

format(round(modelbreedRR,2),nsmall=2)

# Waiting for profiling to be done...

# There were 50 or more warnings (use warnings() to see the first 50)

# > format(round(modelbreedRR,2),nsmall=2)

# RR lower.ci upper.ci

# (Intercept) 1.17 1.09 1.24

# breedAiredale Terrier 1.41 0.00 NA

# breedAkita 1.10 0.43 1.39

# breedAlaskan Husky 1.41 0.00 NA

# breedAlaskan Malamute 1.41 0.00 NA

# breedAmerican Bulldog 0.80 0.47 1.10

# breedAmerican Bully 1.06 0.54 1.35

# breedAustralian Bulldog 1.32 1.03 1.40

# breedAustralian Cattle Dog 0.79 0.57 0.99

# breedAustralian Koolie 0.94 0.45 1.28

# breedAustralian Shepherd 1.41 0.00 NA

# breedAustralian Silky Terrier 0.00 NA 1.41

# breedAustralian Stumpy Tail Cattle Dog 1.41 0.00 NA

# breedAustralian Terrier 1.41 0.00 NA

# breedBandog 0.69 0.26 1.11

# breedBasset Hound 0.00 NA NaN

# breedBeagle 0.83 0.41 1.18

# breedBeaglier 1.41 0.00 NA

# breedBelgian Shepherd 0.77 0.06 1.36

# breedBichon 1.41 0.00 NA

# breedBichoodle 1.41 0.00 NA

# breedBorder Collie 0.94 0.74 1.11

# breedBoston Terrier 1.41 0.00 NA

# breedBoxer 1.14 0.69 1.36

# breedBritish Bulldog 1.21 0.97 1.35

# breedBull Arab 0.84 0.60 1.06

# breedBull Terrier 0.77 0.06 1.36

# breedBulldog 1.41 0.00 NA

# breedBullmastiff 0.81 0.44 1.13

# breedCane Corso 0.77 0.17 1.28

# breedCavachon 1.41 0.00 NA

# breedCavalier King Charles Spaniel 1.15 0.89 1.32

# breedCavashoo 1.41 0.00 NA

# breedCavocker 1.41 0.00 NA

# breedCavoodle 1.30 1.15 1.38

# breedChihuahua 1.12 0.92 1.26

# breedChinese Crested Dog 1.41 0.00 NA

# breedChion 1.41 0.00 NA

# breedChiweenie 1.41 0.00 NA

# breedCocker Spaniel 1.10 0.43 1.39

# breedCorgi 0.00 NA NaN

# breedDachshund 1.10 0.62 1.35

# breedDalmatian 1.41 0.00 NA

# breedDingo 1.41 0.00 NA

# breedDobermann 0.77 0.17 1.28

# breedDogue de Bordeaux 1.04 0.60 1.31

# breedEnglish Cocker Spaniel 0.00 NA NaN

# breedEnglish Mastiff 0.00 NA NaN

# breedEnglish Pointer 1.41 0.00 NA

# breedEnglish Springer Spaniel 0.77 0.25 1.23

# breedFox Terrier 0.84 0.39 1.22

# breedFrench Bulldog 1.31 1.23 1.36

# breedFrenchton 1.41 0.00 NA

# breedFrug 1.41 0.00 NA

# breedGerman Shepherd 1.06 0.88 1.19

# breedGerman Shorthaired Pointer 0.94 0.45 1.28

# breedGerman Spitz<U+00A0> 1.41 0.00 NA

# breedGoldador 1.41 0.00 NA

# breedGolden Retriever 1.16 0.57 1.39

# breedGreat Dane 0.68 0.34 1.02

# breedGreyhound 0.84 0.39 1.22

# breedGriffon Bruxellois 1.41 0.00 NA

# breedGroodle 1.24 0.76 1.40

# breedHarrier 1.41 0.00 NA

# breedHavanese 1.41 0.00 NA

# breedHound 0.00 NA 1.41

# breedHusky 0.99 0.59 1.27

# breedIrish Wolfhound 1.13 0.75 1.34

# breedJack Russell Terrier 1.12 0.91 1.26

# breedJapanese Chin 1.41 0.00 NA

# breedJohnson Bulldog 0.53 0.04 1.22

# breedJug 0.00 NA NaN

# breedKangal Shepherd Dog 0.00 NA NaN

# breedKelpie 0.82 0.63 1.00

# breedLabradogue 1.41 0.00 NA

# breedLabradoodle 1.14 0.69 1.36

# breedLabrador Retriever 1.16 0.96 1.30

# breedLhasa Apso 1.41 0.00 NA

# breedMalchi 1.41 0.00 NA

# breedMalinois 1.10 0.43 1.39

# breedMaltese 1.17 0.97 1.30

# breedMaltese Shih Tzu 1.03 0.64 1.28

# breedMaremma 1.41 0.00 NA

# breedMaremma Sheepdog 0.81 0.46 1.12

# breedMastiff 0.80 0.48 1.09

# breedMiniature Australian Bulldog 1.41 0.00 NA

# breedMiniature Bull Terrier 1.41 0.00 NA

# breedMiniature Cavoodle 1.41 0.00 NA

# breedMiniature Dachshund 1.41 0.00 NA

# breedMiniature Fox Terrier<U+00A0> 1.12 0.82 1.30

# breedMiniature Jack Russell 1.41 0.00 NA

# breedMiniature Labradoodle 1.41 0.00 NA

# breedMiniature Maltese 0.00 NA NaN

# breedMiniature Pinscher 1.41 0.00 NA

# breedMiniature Poodle 1.16 0.57 1.39

# breedMiniature Schnauzer 1.41 0.00 NA

# breedMiniature Spoodle 1.41 0.00 NA

# breedMoodle 1.41 0.00 NA

# breedMurray River Retriever 1.41 0.00 NA

# breedNeapolitan Mastiff 1.41 0.00 NA

# breedNewfoundland 1.41 0.00 NA

# breedPapillon 0.00 NA NaN

# breedPekingese 1.41 0.00 NA

# breedPembroke Welsh Corgi<U+00A0> 1.41 0.00 NA

# breedPinoodle 1.41 0.00 NA

# breedPit Bull Terrier 0.64 0.31 0.99

# breedPomchi 1.41 0.00 NA

# breedPomeranian 1.03 0.74 1.23

# breedPoochin 1.41 0.00 NA

# breedPoodle 1.41 0.00 NA

# breedPug 1.12 0.92 1.26

# breedPugalier 1.41 0.00 NA

# breedPuggle 1.41 0.00 NA

# breedPuli 1.41 0.00 NA

# breedRhodesian Ridgeback 1.06 0.68 1.29

# breedRottweiler 0.87 0.63 1.08

# breedRough Collie 0.99 0.26 1.38

# breedSamoyed 1.41 0.00 NA

# breedSchnauzer 1.41 0.00 NA

# breedSchnoodle 1.41 0.00 NA

# breedShar Pei 0.77 0.34 1.15

# breedShepsky 1.41 0.00 NA

# breedShih Tzu 1.41 0.00 NA

# breedSiberian Husky 0.83 0.41 1.18

# breedSmithfield 1.41 0.00 NA

# breedSouthern Bulldog 1.41 0.00 NA

# breedSpoodle 1.16 0.57 1.39

# breedSt. Bernard 1.41 0.00 NA

# breedStaffordshire Bull Terrier 1.00 0.83 1.14

# breedStaghound 0.62 0.13 1.17

# breedSwiss Shepherd 0.99 0.26 1.38

# breedTenterfield Terrier 1.41 0.00 NA

# breedTerrier 1.41 0.00 NA

# breedToy Cavoodle 1.41 0.00 NA

# breedToy Moodle 1.41 0.00 NA

# breedToy Pomeranian 1.41 0.00 NA

# breedToy Poodle 1.24 1.01 1.36

# breedUnknown 0.77 0.48 1.03

# breedWeimarhund 1.41 0.00 NA

# breedWest Highland White Terrier 1.41 0.00 NA

# breedWhippet 0.77 0.17 1.28

# breedYorkshire Terrier 0.00 NA NaN

##############SELLER(OFFERED BY)

modelofferedby <- glm(microchip ~ offered_by, family = binomial, data = dogadsor)

summary(modelofferedby)

# Deviance Residuals:

# Min 1Q Median 3Q Max

# -2.1183 -1.2948 0.4736 1.0644 1.0644

#

# Coefficients:

# Estimate Std. Error z value Pr(>|z|)

# (Intercept) 2.1314 0.1221 17.453 <2e-16 ***

# offered_byOwner -1.8596 0.1385 -13.427 <2e-16 ***

# offered_byShelter/Rescue -0.4137 0.4283 -0.966 0.334

# ---

# Signif. codes: 0 ‘***’ 0.001 ‘**’ 0.01 ‘*’ 0.05 ‘.’ 0.1 ‘ ’ 1

#

# (Dispersion parameter for binomial family taken to be 1)

#

# Null deviance: 2056.4 on 1707 degrees of freedom

# Residual deviance: 1823.9 on 1705 degrees of freedom

# AIC: 1829.9

#

# Number of Fisher Scoring iterations: 4

# get the confidence interval

offeredbyci <- confint(modelofferedby)

round(exp(cbind(modelofferedby$coef, offeredbyci)), digits = 2)

# 2.5 % 97.5 %

# (Intercept) 8.43 6.68 10.79

# offered_byOwner 0.16 0.12 0.20

# offered_byShelter/Rescue 0.66 0.30 1.66

# >

modelofferedbyRR<-odds_to_rr(modelofferedby)

format(round(modelofferedbyRR,2),nsmall=2)

#

# RR lower.ci upper.ci

# (Intercept) 1.34 1.33 1.36

# offered_byOwner 0.39 0.32 0.47

# offered_byShelter/Rescue 0.87 0.60 1.13

##########ANKC

modelankc <- glm(microchip ~ ankc_registered, family = binomial, data = dogadsor)

summary(modelankc)

# Deviance Residuals:

# Min 1Q Median 3Q Max

# -2.3614 -1.3990 0.4280 0.9709 0.9709

#

# Coefficients:

# Estimate Std. Error z value Pr(>|z|)

# (Intercept) 2.7246 0.2980 9.143 < 2e-16 ***

# ankc_registerednone -2.2173 0.3038 -7.298 2.92e-13 ***

# ankc_registeredother -0.3806 0.3619 -1.052 0.293

# ---

# Signif. codes: 0 ‘***’ 0.001 ‘**’ 0.01 ‘*’ 0.05 ‘.’ 0.1 ‘ ’ 1

#

# (Dispersion parameter for binomial family taken to be 1)

#

# Null deviance: 2056.4 on 1707 degrees of freedom

# Residual deviance: 1876.4 on 1705 degrees of freedom

# AIC: 1882.4

# get the confidence interval

ankcci <- confint(modelankc)

round(exp(cbind(modelankc$coef, ankcci)), digits = 2)

# 2.5 % 97.5 %

# (Intercept) 15.25 8.90 28.91

# ankc_registerednone 0.11 0.06 0.19

# ankc_registeredother 0.68 0.33 1.36

modelankcRR<-odds_to_rr(modelankc)

format(round(modelankcRR,2),nsmall=2)

# RR lower.ci upper.ci

# (Intercept) 1.37 1.35 1.39

# ankc_registerednone 0.30 0.17 0.45

# ankc_registeredother 0.88 0.62 1.08

#########BREEDER ID - STATE REGISTERED

#originally did the chi square for ads that had a breeder ID and didnt have breeder ID (yes, no).

#decided need to change to states that require breeder ID and states that do not require breeder ID.

#######ID_BIN - STATES WITH ID VS WITHOUT

modelidbin <- glm(microchip ~ ID_bin, family = binomial, data = dogadsor)

summary(modelidbin)

# Call:

# glm(formula = microchip ~ ID_bin, family = binomial, data = dogadsor)

#

# Deviance Residuals:

# Min 1Q Median 3Q Max

# -1.6651 -1.6205 0.7585 0.7916 0.7916

#

# Coefficients:

# Estimate Std. Error z value Pr(>|z|)

# (Intercept) 0.99977 0.07324 13.650 <2e-16 ***

# ID_binrequired 0.09885 0.11964 0.826 0.409

# ---

# Signif. codes: 0 ‘***’ 0.001 ‘**’ 0.01 ‘*’ 0.05 ‘.’ 0.1 ‘ ’ 1

#

# (Dispersion parameter for binomial family taken to be 1)

#

# Null deviance: 1774.9 on 1543 degrees of freedom

# Residual deviance: 1774.2 on 1542 degrees of freedom

# (164 observations deleted due to missingness)

# AIC: 1778.2

#

# Number of Fisher Scoring iterations: 4

#

# get the confidence interval

idbinci <- confint(modelidbin)

round(exp(cbind(modelidbin$coef, idbinci)), digits = 2)

# 2.5 % 97.5 %

# (Intercept) 2.72 2.36 3.14

# ID_binrequired 1.10 0.87 1.40

modelidbinRR<-odds_to_rr(modelidbin)

format(round(modelidbinRR,2),nsmall=2)

# RR lower.ci upper.ci

# (Intercept) 1.20 1.18 1.22

# ID_binrequired 1.03 0.96 1.08

#################microchip/age

modelage <- glm(microchip ~ age_bin, family = binomial, data = dogadsor)

summary(modelage)

# Call:

# glm(formula = microchip ~ age_bin, family = binomial, data = dogadsor)

#

# Deviance Residuals:

# Min 1Q Median 3Q Max

# -1.7368 -1.2735 0.7073 0.7510 1.0842

#

# Coefficients:

# Estimate Std. Error z value Pr(>|z|)

# (Intercept) 1.25820 0.09901 12.708 < 2e-16 ***

# age_bin1 - 3 years -1.03506 0.17137 -6.040 1.54e-09 ***

# age_bin3 + years -0.99114 0.21409 -4.629 3.67e-06 ***

# age_bin6 months - 1 year -0.87955 0.19117 -4.601 4.21e-06 ***

# age_bin8 weeks - 6 months -0.13670 0.13788 -0.992 0.321

# age_binUnknown -0.36723 0.31296 -1.173 0.241

# ---

# Signif. codes: 0 ‘***’ 0.001 ‘**’ 0.01 ‘*’ 0.05 ‘.’ 0.1 ‘ ’ 1

#

# (Dispersion parameter for binomial family taken to be 1)

#

# Null deviance: 2056.4 on 1707 degrees of freedom

# Residual deviance: 1994.1 on 1702 degrees of freedom

# AIC: 2006.1

#

# Number of Fisher Scoring iterations: 4

# get the confidence interval

ageci <- confint(modelage)

round(exp(cbind(modelage$coef, ageci)), digits = 2)

#

# 2.5 % 97.5 %

# (Intercept) 3.52 2.91 4.29

# age_bin1 - 3 years 0.36 0.25 0.50

# age_bin3 + years 0.37 0.24 0.57

# age_bin6 months - 1 year 0.41 0.29 0.60

# age_bin8 weeks - 6 months 0.87 0.67 1.14

# age_binUnknown 0.69 0.38 1.31

modelageRR<-odds_to_rr(modelage)

format(round(modelageRR,2),nsmall=2)

#

# RR lower.ci upper.ci

# (Intercept) 1.26 1.23 1.29

# age_bin1 - 3 years 0.66 0.54 0.77

# age_bin3 + years 0.67 0.53 0.82

# age_bin6 months - 1 year 0.71 0.58 0.84

# age_bin8 weeks - 6 months 0.96 0.87 1.04

# age_binUnknown 0.89 0.68 1.07

#####create the model for microchip/price

modelprice <- glm(microchip ~ price_bin, family = binomial, data = dogadsor)

summary(modelprice)

# Call:

# glm(formula = microchip ~ price_bin, family = binomial, data = dogadsor)

#

# Deviance Residuals:

# Min 1Q Median 3Q Max

# -2.2166 -1.1424 0.5241 0.9448 1.4080

#

# Coefficients:

# ` ` Estimate Std. Error z value Pr(>|z|)

# (Intercept) -0.08289 0.15398 -0.538 0.59036

# price_bin$1001 - $3000 1.99858 0.19911 10.038 < 2e-16 ***

# price_bin$251 - $500 0.55289 0.18319 3.018 0.00254 **

# price_bin$3001+ 2.45001 0.33881 7.231 4.79e-13 ***

# price_bin$501 - $1000 0.95239 0.20972 4.541 5.59e-06 ***

# price_binFree -0.44447 0.26054 -1.706 0.08802 .

# price_binUnknown 0.65825 0.28562 2.305 0.02119 *

# ---

# Signif. codes: 0 ‘***’ 0.001 ‘**’ 0.01 ‘*’ 0.05 ‘.’ 0.1 ‘ ’ 1

#

# (Dispersion parameter for binomial family taken to be 1)

#

# Null deviance: 2056.4 on 1707 degrees of freedom

# Residual deviance: 1831.2 on 1701 degrees of freedom

# AIC: 1845.2

#

# Number of Fisher Scoring iterations: 4

# get the confidence interval

priceci <- confint(modelprice)

round(exp(cbind(modelprice$coef, priceci)), digits = 2)

# 2.5 % 97.5 %

# (Intercept) 0.92 0.68 1.24

# price_bin$1001 - $3000 7.38 5.01 10.94

# price_bin$251 - $500 1.74 1.21 2.49

# price_bin$3001+ 11.59 6.17 23.53

# price_bin$501 - $1000 2.59 1.72 3.92

# price_binFree 0.64 0.38 1.06

# price_binUnknown 1.93 1.11 3.41

modelpriceRR<-odds_to_rr(modelprice)

format(round(modelpriceRR,2),nsmall=2)

#

# RR lower.ci upper.ci

# (Intercept) 0.98 0.88 1.06

# price_bin$1001 - $3000 1.33 1.30 1.36

# price_bin$251 - $500 1.14 1.05 1.21

# price_bin$3001+ 1.36 1.32 1.38

# price_bin$501 - $1000 1.22 1.14 1.28

# price_binFree 0.86 0.68 1.02

# price_binUnknown 1.16 1.03 1.26

#########create the model for microchip/desex

#change desexing to categorical

dogadsor$desexed<- factor(dogadsor$desexed)

#check if worked - yes

dogadsor

modelchipdesex <- glm(microchip ~ desexed, family = binomial, data = dogadsor)

summary(modelchipdesex)

# Call:

# glm(formula = microchip ~ desexed, family = binomial, data = dogadsor)

#

# Deviance Residuals:

# Min 1Q Median 3Q Max

# -1.9379 -1.5412 0.8529 0.8529 0.8529

#

# Coefficients:

# Estimate Std. Error z value Pr(>|z|)

# (Intercept) 0.82388 0.05539 14.875 < 2e-16 ***

# desexed1 0.88784 0.22017 4.033 5.52e-05 ***

# ---

# Signif. codes: 0 ‘***’ 0.001 ‘**’ 0.01 ‘*’ 0.05 ‘.’ 0.1 ‘ ’ 1

#

# (Dispersion parameter for binomial family taken to be 1)

#

# Null deviance: 2056.4 on 1707 degrees of freedom

# Residual deviance: 2037.2 on 1706 degrees of freedom

# AIC: 2041.2

#

# Number of Fisher Scoring iterations: 4

# get the confidence interval

chipdesexci <- confint(modelchipdesex)

round(exp(cbind(modelchipdesex$coef, chipdesexci)), digits = 2)

# 2.5 % 97.5 %

# (Intercept) 2.28 2.05 2.54

# desexed1 2.43 1.61 3.82

# >

modelchipdesexRR<-odds_to_rr(modelchipdesex)

format(round(modelchipdesexRR,2),nsmall=2)

# RR lower.ci upper.ci

# (Intercept) 1.19 1.17 1.21

# desexed1 1.21 1.12 1.27

#####################RO FOR DESEXING ################

#need to turn all independant variables into categorical variables.

#pure/cross

#breed

#state

#offered by

#vaccinatiOn

#ankc registered

#state registered

#will use new dataframe so dont mess with old

dogadsordesex <- chidesexdatafame25_08desex6

dogadsordesex

#change pure_cross to categorical

dogadsordesex$pure_cross <- factor(dogadsordesex$pure_cross)

#check if worked - yes

dogadsordesex

#change breed to categorical

dogadsordesex$breed <- factor(dogadsordesex$breed)

#check if worked - yes

dogadsordesex

#change state to categorical

dogadsordesex$state <- factor(dogadsordesex$state)

#check if worked - yes

dogadsordesex

#change offered by to categorical

dogadsordesex$offered_by <- factor(dogadsordesex$offered_by)

#check if worked - yes

dogadsordesex

#change vaccination to categorical

dogadsordesex$vaccination <- factor(dogadsordesex$vaccination)

#check if worked - yes

dogadsordesex

#change ank registered to categorical

dogadsordesex$ankc_registered <- factor(dogadsordesex$ankc_registered)

#check if worked - yes

dogadsordesex

#change state registered to categorical

dogadsordesex$state_registered <- factor(dogadsordesex$state_registered)

#check if worked - yes

dogadsordesex

#change price bin to categorical

dogadsordesex$price_bin <- factor(dogadsordesex$price_bin)

#check if worked - yes

dogadsordesex

#change age bin to categorical

dogadsordesex$age_bin <- factor(dogadsordesex$age_bin)

#check if worked - yes

dogadsordesex

#change microchip to categorical

dogadsordesex$microchip <- factor(dogadsordesex$microchip)

#check if worked - yes

dogadsordesex

#Now we save this dataframe so we have easy access to it in the future

write.csv(dogadsordesex, file = "dogadsordesex.csv", row.names = TRUE)

#loaddogsor - chidataframe (no act.nt etc) but with independent variables as factors

dogadsordesex <- read_csv("C:/Users/Sofia Costa/OneDrive/Adelaide University Hons/Honours Program/R/dogadsordesex.csv")

dogadsordesex

#########create the model for desex/microchip

modeldesexchip <- glm(desexed ~ microchip, family = binomial, data = dogadsordesex)

summary(modeldesexchip)

# Call:

# glm(formula = desexed ~ microchip, family = binomial, data = dogadsordesex)

#

# Deviance Residuals:

# Min 1Q Median 3Q Max

# -0.9576 -0.9576 -0.4912 1.4145 2.0856

#

# Coefficients:

# Estimate Std. Error z value Pr(>|z|)

# (Intercept) -2.0541 0.2124 -9.670 < 2e-16 ***

# microchip1 1.5122 0.2429 6.226 4.8e-10 ***

# ---

# Signif. codes: 0 ‘***’ 0.001 ‘**’ 0.01 ‘*’ 0.05 ‘.’ 0.1 ‘ ’ 1

#

# (Dispersion parameter for binomial family taken to be 1)

#

# Null deviance: 609.94 on 529 degrees of freedom

# Residual deviance: 563.58 on 528 degrees of freedom

# AIC: 567.58

# Number of Fisher Scoring iterations: 4

# get the confidence interval

dchipci <- confint(modeldesexchip)

round(exp(cbind(modeldesexchip$coef, dchipci)), digits = 2)

# 2.5 % 97.5 %

# (Intercept) 0.13 0.08 0.19

# microchip1 4.54 2.86 7.44

#

modeldchipRR<-odds_to_rr(modeldesexchip)

format(round(modeldchipRR,2),nsmall=2)

# RR lower.ci upper.ci

# (Intercept) 0.17 0.11 0.24

# microchip1 2.35 1.92 2.77

#########create the model for desex/states

dogadsordesex$state <- relevel(dogadsordesex$state, ref= "VIC")

table(dogadsordesex$state)

modeldstate <- glm(desexed ~ state, family = binomial, data = dogadsordesex)

summary(modeldstate)

# Call:

# glm(formula = desexed ~ state, family = binomial, data = dogadsordesex)

#

# Deviance Residuals:

# Min 1Q Median 3Q Max

# -0.8752 -0.7924 -0.7443 1.5134 1.9990

#

# Coefficients:

# Estimate Std. Error z value Pr(>|z|)

# (Intercept) -1.14210 0.29655 -3.851 0.000118 ***

# statena -0.71029 0.48224 -1.473 0.140777

# stateNSW 0.14474 0.34404 0.421 0.673970

# stateQLD 0.37996 0.34333 1.107 0.268430

# stateSA 0.10064 0.55985 0.180 0.857334

# stateTAS 0.16127 0.56312 0.286 0.774586

# stateWA -0.05183 0.46717 -0.111 0.911668

# ---

# Signif. codes: 0 ‘***’ 0.001 ‘**’ 0.01 ‘*’ 0.05 ‘.’ 0.1 ‘ ’ 1

#

# (Dispersion parameter for binomial family taken to be 1)

#

# Null deviance: 609.94 on 529 degrees of freedom

# Residual deviance: 601.55 on 523 degrees of freedom

# AIC: 615.55

#

# Number of Fisher Scoring iterations: 4

# get the confidence interval

dstateci <- confint(modeldstate)

round(exp(cbind(modeldstate$coef, dstateci)), digits = 2)

#

#

# 2.5 % 97.5 %

# (Intercept) 0.32 0.17 0.56

# statena 0.49 0.18 1.24

# stateNSW 1.16 0.60 2.32

# stateQLD 1.46 0.76 2.94

# stateSA 1.11 0.35 3.22

# stateTAS 1.17 0.37 3.45

# stateWA 0.95 0.37 2.35

modeldstateRR<-odds_to_rr(modeldstate)

format(round(modeldstateRR,2),nsmall=2)

# RR lower.ci upper.ci

# (Intercept) 0.39 0.22 0.63

# statena 0.57 0.23 1.17

# stateNSW 1.11 0.67 1.72

# stateQLD 1.30 0.81 1.95

# stateSA 1.08 0.42 2.04

# stateTAS 1.12 0.44 2.10

# stateWA 0.96 0.44 1.74

#############DESEX/VACCINATION

modeldvx <- glm(desexed ~ vaccination, family = binomial, data = dogadsordesex)

summary(modeldvx)

# Call:

# glm(formula = desexed ~ vaccination, family = binomial, data = dogadsordesex)

#

# Deviance Residuals:

# Min 1Q Median 3Q Max

# -0.9058 -0.9058 -0.5773 1.4759 1.9360

#

# Coefficients:

# Estimate Std. Error z value Pr(>|z|)

# (Intercept) -1.7075 0.1892 -9.025 < 2e-16 ***

# vaccinationyes 1.0286 0.2236 4.599 4.24e-06 ***

# ---

# Signif. codes: 0 ‘***’ 0.001 ‘**’ 0.01 ‘*’ 0.05 ‘.’ 0.1 ‘ ’ 1

#

# (Dispersion parameter for binomial family taken to be 1)

#

# Null deviance: 609.94 on 529 degrees of freedom

# Residual deviance: 586.72 on 528 degrees of freedom

# AIC: 590.72

#

# Number of Fisher Scoring iterations: 4

# get the confidence interval

dvxci <- confint(modeldvx)

round(exp(cbind(modeldvx$coef, dvxci)), digits = 2)

# # 2.5 % 97.5 %

# (Intercept) 0.18 0.12 0.26

# vaccinationyes 2.80 1.82 4.39

modeldvxRR<-odds_to_rr(modeldvx)

format(round(modeldvxRR,2),nsmall=2)

#

# RR lower.ci upper.ci

# (Intercept) 0.23 0.16 0.32

# vaccinationyes 1.90 1.50 2.32

########DESEX/PURE/CROSS

modeldpure <- glm(desexed ~ pure_cross, family = binomial, data = dogadsordesex)

summary(modeldpure)

# Call:

# glm(formula = desexed ~ pure_cross, family = binomial, data = dogadsordesex)

#

# Deviance Residuals:

# Min 1Q Median 3Q Max

# -1.0579 -0.6636 -0.6636 1.3179 1.8008

#

# Coefficients:

# Estimate Std. Error z value Pr(>|z|)

# (Intercept) -0.5550 0.1593 -3.483 0.000496 ***

# pure_crossdesigner 0.2185 0.6068 0.360 0.718767

# pure_crosspure -0.8463 0.2104 -4.022 5.76e-05 ***

# pure_crossunknown 0.2673 0.5631 0.475 0.634972

# ---

# Signif. codes: 0 ‘***’ 0.001 ‘**’ 0.01 ‘*’ 0.05 ‘.’ 0.1 ‘ ’ 1

#

# (Dispersion parameter for binomial family taken to be 1)

#

# Null deviance: 609.94 on 529 degrees of freedom

# Residual deviance: 590.53 on 526 degrees of freedom

# AIC: 598.53

#

# Number of Fisher Scoring iterations: 4

# get the confidence interval

dpureci <- confint(modeldpure)

round(exp(cbind(modeldpure$coef, dpureci)), digits = 2)

# 2.5 % 97.5 %

# (Intercept) 0.57 0.42 0.78

# pure_crossdesigner 1.24 0.36 4.06

# pure_crosspure 0.43 0.28 0.65

# pure_crossunknown 1.31 0.41 3.93

modeldpureRR<-odds_to_rr(modeldpure)

format(round(modeldpureRR,2),nsmall=2)

# RR lower.ci upper.ci

# (Intercept) 0.65 0.49 0.83

# pure_crossdesigner 1.17 0.43 2.25

# pure_crosspure 0.50 0.35 0.71

# pure_crossunknown 1.21 0.49 2.22

##############BREED

#make American Staffordshire Terrier the reference breed

dogadsordesex$breed <- relevel(dogadsordesex$breed, ref= "American Staffordshire Terrier")

table(dogadsordesex$breed)

#confirmed

modeldbreed <- glm(desexed ~ breed, family = binomial, data = dogadsordesex)

summary(modeldbreed)

# Call:

# glm(formula = desexed ~ breed, family = binomial, data = dogadsordesex)

#

# Deviance Residuals:

# Min 1Q Median 3Q Max

# -1.48230 -0.77265 -0.51678 0.00013 2.29741

#

# Coefficients:

# Estimate Std. Error z value Pr(>|z|)

# (Intercept) -0.82098 0.36181 -2.269 0.0233 *

# breedAkita -17.74509 4612.20200 -0.004 0.9969

# breedAlaskan Husky -17.74509 6522.63861 -0.003 0.9978

# breedAmerican Bulldog -1.25846 1.12067 -1.123 0.2615

# breedAmerican Bully 0.82098 1.45976 0.562 0.5738

# breedAustralian Bulldog -17.74509 3765.84717 -0.005 0.9962

# breedAustralian Cattle Dog 0.12783 0.56428 0.227 0.8208

# breedAustralian Koolie -17.74509 4612.20200 -0.004 0.9969

# breedAustralian Silky Terrier -17.74509 6522.63861 -0.003 0.9978

# breedBandog -17.74509 3765.84717 -0.005 0.9962

# breedBasset Hound -17.74509 6522.63861 -0.003 0.9978

# breedBeagle -0.56531 1.17512 -0.481 0.6305

# breedBorder Collie -1.74397 1.09901 -1.587 0.1125

# breedBoxer 0.82098 1.45976 0.562 0.5738

# breedBritish Bulldog 0.12783 0.93857 0.136 0.8917

# breedBull Arab 0.46431 0.61136 0.759 0.4476

# breedBull Terrier -17.74509 4612.20200 -0.004 0.9969

# breedBullmastiff 0.82098 1.06344 0.772 0.4401

# breedCane Corso -17.74509 4612.20200 -0.004 0.9969

# breedCavalier King Charles Spaniel 1.22645 0.98196 1.249 0.2117

# breedCavoodle 0.12783 1.27707 0.100 0.9203

# breedChihuahua -0.27763 0.68135 -0.407 0.6837

# breedChion -17.74509 6522.63861 -0.003 0.9978

# breedCocker Spaniel 0.82098 1.45976 0.562 0.5738

# breedCorgi 19.38705 6522.63861 0.003 0.9976

# breedDachshund 0.12783 1.27707 0.100 0.9203

# breedDalmatian -17.74509 4612.20200 -0.004 0.9969

# breedDobermann 19.38705 4612.20200 0.004 0.9966

# breedDogue de Bordeaux -17.74509 4612.20200 -0.004 0.9969

# breedEnglish Mastiff -17.74509 6522.63861 -0.003 0.9978

# breedEnglish Pointer 1.51413 1.27707 1.186 0.2358

# breedEnglish Springer Spaniel 19.38705 4612.20201 0.004 0.9966

# breedFox Terrier 0.53330 0.84513 0.631 0.5280

# breedFrench Bulldog -1.07614 0.71711 -1.501 0.1334

# breedGerman Shepherd -0.35767 0.54256 -0.659 0.5097

# breedGerman Shorthaired Pointer -0.56531 1.17512 -0.481 0.6305

# breedGerman Spitz<U+00A0> -17.74509 6522.63861 -0.003 0.9978

# breedGolden Retriever 0.82098 1.45976 0.562 0.5738

# breedGreat Dane -1.37624 1.11446 -1.235 0.2169

# breedGreyhound -1.12493 1.12861 -0.997 0.3189

# breedGroodle -17.74509 6522.63861 -0.003 0.9978

# breedHarrier -17.74509 6522.63861 -0.003 0.9978

# breedHusky -0.56531 1.17512 -0.481 0.6305

# breedIrish Wolfhound 19.38705 2662.85608 0.007 0.9942

# breedJack Russell Terrier -17.74509 2306.10102 -0.008 0.9939

# breedJapanese Chin -17.74509 6522.63861 -0.003 0.9978

# breedJohnson Bulldog -17.74509 4612.20200 -0.004 0.9969

# breedKangal Shepherd Dog -17.74509 6522.63861 -0.003 0.9978

# breedKelpie -0.23507 0.54716 -0.430 0.6675

# breedLabradoodle 19.38705 4612.20201 0.004 0.9966

# breedLabrador Retriever -0.78846 0.85493 -0.922 0.3564

# breedMalinois 19.38705 6522.63861 0.003 0.9976

# breedMaltese -1.12493 1.12861 -0.997 0.3189

# breedMaltese Shih Tzu -17.74509 4612.20200 -0.004 0.9969

# breedMaremma -17.74509 6522.63861 -0.003 0.9978

# breedMaremma Sheepdog 0.41552 0.98196 0.423 0.6722

# breedMastiff 0.53330 0.84513 0.631 0.5280

# breedMiniature Bull Terrier 19.38705 6522.63861 0.003 0.9976

# breedMiniature Dachshund -17.74509 4612.20200 -0.004 0.9969

# breedMiniature Fox Terrier<U+00A0> -0.27763 0.89307 -0.311 0.7559

# breedMiniature Jack Russell -17.74509 6522.63861 -0.003 0.9978

# breedMiniature Labradoodle -17.74509 6522.63861 -0.003 0.9978

# breedMiniature Pinscher -17.74509 6522.63861 -0.003 0.9978

# breedMiniature Poodle -17.74509 6522.63861 -0.003 0.9978

# breedMoodle 0.82098 1.45976 0.562 0.5738

# breedNeapolitan Mastiff 0.82098 1.45976 0.562 0.5738

# breedPapillon -17.74509 6522.63861 -0.003 0.9978

# breedPit Bull Terrier -17.74509 1966.64957 -0.009 0.9928

# breedPomeranian 0.12783 0.71127 0.180 0.8574

# breedPoodle 0.12783 1.27707 0.100 0.9203

# breedPug -0.64536 0.73564 -0.877 0.3803

# breedRhodesian Ridgeback 0.12783 0.93857 0.136 0.8917

# breedRottweiler -1.25846 0.83271 -1.511 0.1307

# breedRough Collie -17.74509 4612.20200 -0.004 0.9969

# breedSamoyed -17.74509 6522.63861 -0.003 0.9978

# breedSchnauzer -17.74509 6522.63861 -0.003 0.9978

# breedShar Pei -17.74509 3765.84717 -0.005 0.9962

# breedSiberian Husky -0.27763 1.21006 -0.229 0.8185

# breedSpoodle 19.38705 6522.63862 0.003 0.9976

# breedStaffordshire Bull Terrier 0.04779 0.50270 0.095 0.9243

# breedStaghound -17.74509 4612.20200 -0.004 0.9969

# breedSwiss Shepherd 0.82098 1.45976 0.562 0.5738

# breedToy Poodle -0.97078 1.13911 -0.852 0.3941

# breedUnknown 1.04412 0.59658 1.750 0.0801 .

# breedWhippet -17.74509 6522.63861 -0.003 0.9978

# breedYorkshire Terrier -17.74509 6522.63861 -0.003 0.9978

# ---

# Signif. codes: 0 ‘***’ 0.001 ‘**’ 0.01 ‘*’ 0.05 ‘.’ 0.1 ‘ ’ 1

#

# (Dispersion parameter for binomial family taken to be 1)

#

# Null deviance: 609.94 on 529 degrees of freedom

# Residual deviance: 486.81 on 444 degrees of freedom

# AIC: 658.81

#

# Number of Fisher Scoring iterations: 17

# get the confidence interval

dbreedci <- confint(modeldbreed)

round(exp(cbind(modeldbreed$coef, dbreedci)), digits = 2)

# Waiting for profiling to be done...

# There were 50 or more warnings (use warnings() to see the first 50)

# > round(exp(cbind(modeldbreed$coef, dbreedci)), digits = 2)

# 2.5 % 97.5 %

# (Intercept) 0.44 0.21 8.700000e-01

# breedAkita 0.00 NA 1.714119e+306

# breedAlaskan Husky 0.00 NA Inf

# breedAmerican Bulldog 0.28 0.01 1.830000e+00

# breedAmerican Bully 2.27 0.08 6.109000e+01

# breedAustralian Bulldog 0.00 NA 1.303401e+205

# breedAustralian Cattle Dog 1.14 0.37 3.430000e+00

# breedAustralian Koolie 0.00 NA 1.714119e+306

# breedAustralian Silky Terrier 0.00 NA Inf

# breedBandog 0.00 NA 1.303401e+205

# breedBasset Hound 0.00 NA Inf

# breedBeagle 0.57 0.03 4.420000e+00

# breedBorder Collie 0.17 0.01 1.050000e+00

# breedBoxer 2.27 0.08 6.109000e+01

# breedBritish Bulldog 1.14 0.14 6.780000e+00

# breedBull Arab 1.59 0.47 5.300000e+00

# breedBull Terrier 0.00 NA 1.714119e+306

# breedBullmastiff 2.27 0.25 2.103000e+01

# breedCane Corso 0.00 NA 1.714119e+306

# breedCavalier King Charles Spaniel 3.41 0.50 2.879000e+01

# breedCavoodle 1.14 0.05 1.312000e+01

# breedChihuahua 0.76 0.18 2.760000e+00

# breedChion 0.00 NA Inf

# breedCocker Spaniel 2.27 0.08 6.109000e+01

# breedCorgi 262838165.53 0.00 NA

# breedDachshund 1.14 0.05 1.312000e+01

# breedDalmatian 0.00 NA 1.714119e+306

# breedDobermann 262838165.34 0.00 NA

# breedDogue de Bordeaux 0.00 NA 1.714119e+306

# breedEnglish Mastiff 0.00 NA Inf

# breedEnglish Pointer 4.55 0.40 1.040600e+02

# breedEnglish Springer Spaniel 262838166.35 0.00 NA

# breedFox Terrier 1.70 0.29 9.060000e+00

# breedFrench Bulldog 0.34 0.07 1.270000e+00

# breedGerman Shepherd 0.70 0.23 2.010000e+00

# breedGerman Shorthaired Pointer 0.57 0.03 4.420000e+00

# breedGerman Spitz<U+00A0> 0.00 NA Inf

# breedGolden Retriever 2.27 0.08 6.109000e+01

# breedGreat Dane 0.25 0.01 1.600000e+00

# breedGreyhound 0.32 0.02 2.150000e+00

# breedGroodle 0.00 NA Inf

# breedHarrier 0.00 NA Inf

# breedHusky 0.57 0.03 4.420000e+00

# breedIrish Wolfhound 262838165.48 0.00 NA

# breedJack Russell Terrier 0.00 NA 1.833543e+76

# breedJapanese Chin 0.00 NA Inf

# breedJohnson Bulldog 0.00 NA 1.714119e+306

# breedKangal Shepherd Dog 0.00 NA Inf

# breedKelpie 0.79 0.26 2.300000e+00

# breedLabradoodle 262838166.37 0.00 NA

# breedLabrador Retriever 0.45 0.06 2.110000e+00

# breedMalinois 262838165.49 0.00 NA

# breedMaltese 0.32 0.02 2.150000e+00

# breedMaltese Shih Tzu 0.00 NA 1.714119e+306

# breedMaremma 0.00 NA Inf

# breedMaremma Sheepdog 1.52 0.18 1.044000e+01

# breedMastiff 1.70 0.29 9.060000e+00

# breedMiniature Bull Terrier 262838165.47 0.00 NA

# breedMiniature Dachshund 0.00 NA 1.714119e+306

# breedMiniature Fox Terrier<U+00A0> 0.76 0.10 3.920000e+00

# breedMiniature Jack Russell 0.00 NA Inf

# breedMiniature Labradoodle 0.00 NA Inf

# breedMiniature Pinscher 0.00 NA Inf

# breedMiniature Poodle 0.00 NA Inf

# breedMoodle 2.27 0.08 6.109000e+01

# breedNeapolitan Mastiff 2.27 0.08 6.109000e+01

# breedPapillon 0.00 NA Inf

# breedPit Bull Terrier 0.00 NA 1.631834e+47

# breedPomeranian 1.14 0.26 4.460000e+00

# breedPoodle 1.14 0.05 1.312000e+01

# breedPug 0.52 0.10 2.040000e+00

# breedRhodesian Ridgeback 1.14 0.14 6.780000e+00

# breedRottweiler 0.28 0.04 1.240000e+00

# breedRough Collie 0.00 NA 1.714119e+306

# breedSamoyed 0.00 NA Inf

# breedSchnauzer 0.00 NA Inf

# breedShar Pei 0.00 NA 1.303401e+205

# breedSiberian Husky 0.76 0.04 6.700000e+00

# breedSpoodle 262838166.30 0.00 NA

# breedStaffordshire Bull Terrier 1.05 0.39 2.840000e+00

# breedStaghound 0.00 NA 1.714120e+306

# breedSwiss Shepherd 2.27 0.08 6.109000e+01

# breedToy Poodle 0.38 0.02 2.600000e+00

# breedUnknown 2.84 0.89 9.450000e+00

# breedWhippet 0.00 NA Inf

# breedYorkshire Terrier 0.00 NA Inf

modeldbreedRR<-odds_to_rr(modeldbreed)

format(round(modeldbreedRR,2),nsmall=2)

#

# There were 50 or more warnings (use warnings() to see the first 50)

# > format(round(modeldbreedRR,2),nsmall=2)

# RR lower.ci upper.ci

# (Intercept) 0.52 0.26 0.90

# breedAkita 0.00 NA 3.81

# breedAlaskan Husky 0.00 NA NaN

# breedAmerican Bulldog 0.35 0.02 1.50

# breedAmerican Bully 1.70 0.11 3.65

# breedAustralian Bulldog 0.00 NA 3.81

# breedAustralian Cattle Dog 1.10 0.44 2.10

# breedAustralian Koolie 0.00 NA 3.81

# breedAustralian Silky Terrier 0.00 NA NaN

# breedBandog 0.00 NA 3.81

# breedBasset Hound 0.00 NA NaN

# breedBeagle 0.64 0.04 2.33

# breedBorder Collie 0.22 0.01 1.04

# breedBoxer 1.70 0.11 3.65

# breedBritish Bulldog 1.10 0.18 2.69

# breedBull Arab 1.38 0.55 2.49

# breedBull Terrier 0.00 NA 3.81

# breedBullmastiff 1.70 0.31 3.36

# breedCane Corso 0.00 NA 3.81

# breedCavalier King Charles Spaniel 2.09 0.57 3.47

# breedCavoodle 1.10 0.07 3.14

# breedChihuahua 0.81 0.23 1.89

# breedChion 0.00 NA NaN

# breedCocker Spaniel 1.70 0.11 3.65

# breedCorgi 3.81 0.00 NA

# breedDachshund 1.10 0.07 3.14

# breedDalmatian 0.00 NA 3.81

# breedDobermann 3.81 0.00 NA

# breedDogue de Bordeaux 0.00 NA 3.81

# breedEnglish Mastiff 0.00 NA NaN

# breedEnglish Pointer 2.36 0.47 3.71

# breedEnglish Springer Spaniel 3.81 0.00 NA

# breedFox Terrier 1.44 0.36 2.91

# breedFrench Bulldog 0.41 0.09 1.18

# breedGerman Shepherd 0.76 0.29 1.59

# breedGerman Shorthaired Pointer 0.64 0.04 2.33

# breedGerman Spitz<U+00A0> 0.00 NA NaN

# breedGolden Retriever 1.70 0.11 3.65

# breedGreat Dane 0.31 0.02 1.38

# breedGreyhound 0.39 0.02 1.65

# breedGroodle 0.00 NA NaN

# breedHarrier 0.00 NA NaN

# breedHusky 0.64 0.04 2.33

# breedIrish Wolfhound 3.81 0.00 NA

# breedJack Russell Terrier 0.00 NA 3.81

# breedJapanese Chin 0.00 NA NaN

# breedJohnson Bulldog 0.00 NA 3.81

# breedKangal Shepherd Dog 0.00 NA NaN

# breedKelpie 0.84 0.33 1.71

# breedLabradoodle 3.81 0.00 NA

# breedLabrador Retriever 0.53 0.08 1.63

# breedMalinois 3.81 0.00 NA

# breedMaltese 0.39 0.02 1.65

# breedMaltese Shih Tzu 0.00 NA 3.81

# breedMaremma 0.00 NA NaN

# breedMaremma Sheepdog 1.33 0.23 3.00

# breedMastiff 1.44 0.36 2.91

# breedMiniature Bull Terrier 3.81 0.00 NA

# breedMiniature Dachshund 0.00 NA 3.81

# breedMiniature Fox Terrier<U+00A0> 0.81 0.13 2.22

# breedMiniature Jack Russell 0.00 NA NaN

# breedMiniature Labradoodle 0.00 NA NaN

# breedMiniature Pinscher 0.00 NA NaN

# breedMiniature Poodle 0.00 NA NaN

# breedMoodle 1.70 0.11 3.65

# breedNeapolitan Mastiff 1.70 0.11 3.65

# breedPapillon 0.00 NA NaN

# breedPit Bull Terrier 0.00 NA 3.81

# breedPomeranian 1.10 0.32 2.34

# breedPoodle 1.10 0.07 3.14

# breedPug 0.60 0.14 1.60

# breedRhodesian Ridgeback 1.10 0.18 2.69

# breedRottweiler 0.35 0.05 1.17

# breedRough Collie 0.00 NA 3.81

# breedSamoyed 0.00 NA NaN

# breedSchnauzer 0.00 NA NaN

# breedShar Pei 0.00 NA 3.81

# breedSiberian Husky 0.81 0.05 2.69

# breedSpoodle 3.81 0.00 NA

# breedStaffordshire Bull Terrier 1.04 0.46 1.92

# breedStaghound 0.00 NA 3.81

# breedSwiss Shepherd 1.70 0.11 3.65

# breedToy Poodle 0.45 0.03 1.83

# breedUnknown 1.92 0.92 2.94

# breedWhippet 0.00 NA NaN

# breedYorkshire Terrier 0.00 NA NaN

################SELLER (OFFERED BY )

modeldofferedby <- glm(desexed ~ offered_by, family = binomial, data = dogadsordesex)

summary(modeldofferedby)

# Call:

# glm(formula = desexed ~ offered_by, family = binomial, data = dogadsordesex)

#

# Deviance Residuals:

# Min 1Q Median 3Q Max

# -2.1612 -0.7459 -0.7459 0.4512 2.0843

#

# Coefficients:

# Estimate Std. Error z value Pr(>|z|)

# (Intercept) -2.0513 0.3541 -5.793 6.92e-09 ***

# offered_byOwner 0.9142 0.3719 2.458 0.014 *

# offered_byShelter/Rescue 4.2849 0.7031 6.094 1.10e-09 ***

# ---

# Signif. codes: 0 ‘***’ 0.001 ‘**’ 0.01 ‘*’ 0.05 ‘.’ 0.1 ‘ ’ 1

#

# (Dispersion parameter for binomial family taken to be 1)

#

# Null deviance: 609.94 on 529 degrees of freedom

# Residual deviance: 541.40 on 527 degrees of freedom

# AIC: 547.4

#

# Number of Fisher Scoring iterations: 4

# get the confidence interval

dofferedbyci <- confint(modeldofferedby)

round(exp(cbind(modeldofferedby$coef, dofferedbyci)), digits = 2)

# 2.5 % 97.5 %

# (Intercept) 0.13 0.06 0.24

# offered_byOwner 2.49 1.26 5.52

# offered_byShelter/Rescue 72.59 20.85 351.46

modeldofferedbyRR<-odds_to_rr(modeldofferedby)

format(round(modeldofferedbyRR,2),nsmall=2)

# RR lower.ci upper.ci

# (Intercept) 0.17 0.08 0.30

# offered_byOwner 1.79 1.18 2.53

# offered_byShelter/Rescue 3.67 3.36 3.78

###########BREEDER ORGANIATION (ANKC)

modeldankc <- glm(desexed ~ ankc_registered, family = binomial, data = dogadsordesex)

summary(modeldankc)

# Call:

# glm(formula = desexed ~ ankc_registered, family = binomial, data = dogadsordesex)

#

# Deviance Residuals:

# Min 1Q Median 3Q Max

# -0.8075 -0.8075 -0.8075 1.5995 2.5211

#

# Coefficients:

# Estimate Std. Error z value Pr(>|z|)

# (Intercept) -3.135 1.021 -3.070 0.00214 **

# ankc_registerednone 2.182 1.026 2.126 0.03349 *

# ankc_registeredother 1.609 1.134 1.419 0.15592

# ---

# Signif. codes: 0 ‘***’ 0.001 ‘**’ 0.01 ‘*’ 0.05 ‘.’ 0.1 ‘ ’ 1

#

# (Dispersion parameter for binomial family taken to be 1)

#

# Null deviance: 609.94 on 529 degrees of freedom

# Residual deviance: 599.86 on 527 degrees of freedom

# AIC: 605.86

#

# Number of Fisher Scoring iterations: 5

# get the confidence interval

dankcci <- confint(modeldankc)

round(exp(cbind(modeldankc$coef, dankcci)), digits = 2)

# 2.5 % 97.5 %

# (Intercept) 0.04 0.00 0.21

# ankc_registerednone 8.87 1.84 159.44

# ankc_registeredother 5.00 0.73 99.80

modeldankcRR<-odds_to_rr(modeldankc)

format(round(modeldankcRR,2),nsmall=2)

# RR lower.ci upper.ci

# (Intercept) 0.06 0.00 0.26

# ankc_registerednone 2.89 1.51 3.75

# ankc_registeredother 2.44 0.79 3.71

########create the model for desex/id bin

modeldidbin <- glm(desexed ~ ID_bin, family = binomial, data = dogadsordesex)

summary(modeldidbin)

# Call:

# glm(formula = desexed ~ ID_bin, family = binomial, data = dogadsordesex)

#

# Deviance Residuals:

# Min 1Q Median 3Q Max

# -0.8627 -0.8627 -0.7734 1.5289 1.6449

#

# Coefficients:

# Estimate Std. Error z value Pr(>|z|)

# (Intercept) -1.0538 0.1332 -7.911 2.56e-15 ***

# ID_binrequired 0.2571 0.2101 1.224 0.221

# ---

# Signif. codes: 0 ‘***’ 0.001 ‘**’ 0.01 ‘*’ 0.05 ‘.’ 0.1 ‘ ’ 1

#

# (Dispersion parameter for binomial family taken to be 1)

#

# Null deviance: 556.89 on 470 degrees of freedom

# Residual deviance: 555.40 on 469 degrees of freedom

# (59 observations deleted due to missingness)

# AIC: 559.4

#

# Number of Fisher Scoring iterations: 4

#get the confidence interval

didbinci <- confint(modeldidbin)

round(exp(cbind(modeldidbin$coef, didbinci)), digits = 2)

# 2.5 % 97.5 %

# (Intercept) 0.35 0.27 0.45

# ID_binrequired 1.29 0.85 1.95

modeldidbinRR<-odds_to_rr(modeldidbin)

format(round(modeldidbinRR,2),nsmall=2)

# RR lower.ci upper.ci

# (Intercept) 0.43 0.34 0.53

# ID_binrequired 1.20 0.89 1.54

########create the model for desex/price

modeldprice <- glm(desexed ~ price_bin, family = binomial, data = dogadsordesex)

summary(modeldprice)

# Call:

# glm(formula = desexed ~ price_bin, family = binomial, data = dogadsordesex)

#

# Deviance Residuals:

# Min 1Q Median 3Q Max

# -1.0776 -0.8758 -0.6280 1.2806 2.4864

#

# Coefficients:

# Estimate Std. Error z value Pr(>|z|)

# (Intercept) -0.2392 0.2198 -1.088 0.276378

# price_bin$1001 - $3000 -1.2843 0.3463 -3.708 0.000209 ***

# price_bin$251 - $500 -0.5214 0.2871 -1.816 0.069383 .

# price_bin$3001+ -2.8053 1.0468 -2.680 0.007362 **

# price_bin$501 - $1000 -1.3855 0.3747 -3.698 0.000217 ***

# price_binFree -0.6603 0.3270 -2.019 0.043465 *

# price_binUnknown -1.7067 0.5779 -2.953 0.003147 **

# ---

# Signif. codes: 0 ‘***’ 0.001 ‘**’ 0.01 ‘*’ 0.05 ‘.’ 0.1 ‘ ’ 1

#

# (Dispersion parameter for binomial family taken to be 1)

#

# Null deviance: 609.94 on 529 degrees of freedom

# Residual deviance: 576.20 on 523 degrees of freedom

# AIC: 590.2

#

# Number of Fisher Scoring iterations: 5

#get the confidence interval

dpriceci <- confint(modeldprice)

round

#

# 2.5 % 97.5 %

# (Intercept) 0.79 0.51 1.21

# price_bin$1001 - $3000 0.28 0.14 0.54

# price_bin$251 - $500 0.59 0.34 1.04

# price_bin$3001+ 0.06 0.00 0.31

# price_bin$501 - $1000 0.25 0.12 0.51

# price_binFree 0.52 0.27 0.98

# price_binUnknown 0.18 0.05 0.51

modeldpriceRR<-odds_to_rr(modeldprice)

format(round(modeldpriceRR,2),nsmall=2)

#

# RR lower.ci upper.ci

# (Intercept) 0.83 0.58 1.15

# price_bin$1001 - $3000 0.34 0.18 0.61

# price_bin$251 - $500 0.66 0.41 1.03

# price_bin$3001+ 0.08 0.00 0.38

# price_bin$501 - $1000 0.31 0.15 0.59

# price_binFree 0.59 0.33 0.98

# price_binUnknown 0.23 0.07 0.59

# >

########create the model for desex/age

modeldage <- glm(desexed ~ age_bin, family = binomial, data = dogadsordesex)

summary(modeldage)

# Call:

# glm(formula = desexed ~ age_bin, family = binomial, data = dogadsordesex)

#

# Deviance Residuals:

# Min 1Q Median 3Q Max

# -1.0371 -0.8274 -0.5931 1.3246 2.0305

#

# Coefficients:

# Estimate Std. Error z value Pr(>|z|)

# (Intercept) -0.8961 0.1532 -5.849 4.94e-09 ***

# age_bin3 + years 0.5566 0.2447 2.274 0.02296 *

# age_bin6 months - 1 year -0.7526 0.2668 -2.821 0.00479 **

# age_binUnknown -1.0292 0.4326 -2.379 0.01736 *

# ---

# Signif. codes: 0 ‘***’ 0.001 ‘**’ 0.01 ‘*’ 0.05 ‘.’ 0.1 ‘ ’ 1

#

# (Dispersion parameter for binomial family taken to be 1)

#

# Null deviance: 609.94 on 529 degrees of freedom

# Residual deviance: 581.57 on 526 degrees of freedom

# AIC: 589.57

#

# Number of Fisher Scoring iterations: 4

#get the confidence interval

dageci <- confint(modeldage)

round(exp(cbind(modeldage$coef, dageci)), digits = 2)

# 2.5 % 97.5 %

# (Intercept) 0.41 0.30 0.55

# age_bin3 + years 1.74 1.08 2.82

# age_bin6 months - 1 year 0.47 0.28 0.79

# age_binUnknown 0.36 0.14 0.79

modeldageRR<-odds_to_rr(modeldage)

format(round(modeldageRR,2),nsmall=2)

# RR lower.ci upper.ci

# (Intercept) 0.48 0.37 0.62

# age_bin3 + years 1.46 1.06 1.91

# age_bin6 months - 1 year 0.55 0.34 0.83

# age_binUnknown 0.43 0.18 0.83

########create the model for desex/breeder id (state registered)

modeldstateid <- glm(desexed ~ state_registered, family = binomial, data = dogadsordesex)

summary(modeldstateid)

# Call:

# glm(formula = desexed ~ state_registered, family = binomial,

# data = dogadsordesex)

#

# Deviance Residuals:

# Min 1Q Median 3Q Max

# -1.1073 -0.7457 -0.7457 1.2491 1.6827

#

# Coefficients:

# Estimate Std. Error z value Pr(>|z|)

# (Intercept) -1.1377 0.1062 -10.709 < 2e-16 ***

# state_registeredyes 0.9707 0.3085 3.146 0.00166 **

# ---

# Signif. codes: 0 ‘***’ 0.001 ‘**’ 0.01 ‘*’ 0.05 ‘.’ 0.1 ‘ ’ 1

#

# (Dispersion parameter for binomial family taken to be 1)

#

# Null deviance: 609.94 on 529 degrees of freedom

# Residual deviance: 600.47 on 528 degrees of freedom

# AIC: 604.47

#

# Number of Fisher Scoring iterations: 4

dstateidci <- confint(modeldstateid)

round(exp(cbind(modeldstateid$coef, dstateidci)), digits = 2)

#

# 2.5 % 97.5 %

# (Intercept) 0.32 0.26 0.39

# state_registeredyes 2.64 1.43 4.83

modeldstateidRR<-odds_to_rr(modeldstateid)

format(round(modeldstateidRR,2),nsmall=2)

# RR lower.ci upper.ci

# (Intercept) 0.39 0.32 0.47

# state_registeredyes 1.85 1.29 2.41

# >

################desex - REDOING THE CHI SQ ANALYSIS and RO and RR WITH DOGADSRA###############

#all desexing analysis will need to be for dogs over 6 months of age

#so im going to create a new datafra,e for dgs who are over 6 months but is also

#the same as dogsadsra

dogadsra_d <- dogadsra

dogadsra_d

#remove puppies uder 8 weeks

dogadsra_d <- dogadsra_d[!(dogadsra_d$age_bin == "< 8 weeks old"), ]

dogadsra_d

#emove puppies under 6 months

dogadsra_d <- dogadsra_d[!(dogadsra_d$age_bin == "8 weeks - 6 months"), ]

dogadsra_d

agegroups <- count(dogadsra_d$age_bin)

agegroups

#save the new dataset

write.csv(dogadsra_d, file = "dogadsra_d.csv", row.names = TRUE)

####confirm all variables are factors

#change pure_cross to categorical

dogadsra_d$pure_cross <- factor(dogadsra_d$pure_cross)

#check if worked - yes

dogadsra_d

#change desexed to categorical

dogadsra_d$desexed <- factor(dogadsra_d$desexed)

#check if worked - yes

dogadsra_d

#change state to categorical

dogadsra_d$state <- factor(dogadsra_d$state)

#check if worked - yes

dogadsra_d

#change offered by to categorical

dogadsra_d$offered_by <- factor(dogadsra_d$offered_by)

#check if worked - yes

dogadsra_d

#change vaccination to categorical

dogadsra_d$vaccination <- factor(dogadsra_d$vaccination)

#check if worked - yes

dogadsra_d

#change ank registered to categorical

dogadsra_d$breeder_org_affiliation <- factor(dogadsra_d$breeder_org_affiliation)

#check if worked - yes

dogadsra_d

#change state registered to categorical

dogadsra_d$state_registered <- factor(dogadsra_d$state_registered)

#check if worked - yes

dogadsra_d

#change state ID to categorical

dogadsra_d$ID_bin <- factor(dogadsra_d$ID_bin)

#check if worked - yes

dogadsra_d

#change age bin to categorical

dogadsra_d$age_bin <- factor(dogadsra_d$age_bin)

#check if worked - yes

dogadsra_d

#change price bin to categorical

dogadsra_d$price_bin <- factor(dogadsra_d$price_bin)

#check if worked - yes

dogadsra_d

#change microchip to categorical

dogadsra_d$microchip <- factor(dogadsra_d$microchip)

#check if worked - yes

dogadsra_d

dogadsra_d <-

dogadsra_d %>%

mutate(

ID_bin = case_when(

state == "SA" ~ "required",

state == "NSW" ~ "not required",

state == "QLD" ~ "required",

state == "VIC" ~ "required",

state == "WA" ~ "not required",

state == "TAS" ~ "not required",

is.na(state) ~ "unclear",

)

)

dogadsra_d

#############desexing and microchip

chi.desex.microchip_ra<- table(dogadsra_d$desexed, dogadsra_d$microchip)

chi.desex.microchip_ra

# 0 1

# 0 192 196

# 1 25 86

chisq.test(chi.desex.microchip_ra)

# Pearson's Chi-squared test with Yates' continuity correction

#

# data: chi.desex.microchip_ra

# X-squared = 24.445, df = 1, p-value = 7.647e-07

modelchiprad <- glm(desexed ~ microchip, family = binomial, data = dogadsra_d)

summary(modelchiprad)

#

# Call:

# glm(formula = desexed ~ microchip, family = binomial, data = dogadsra_d)

#

# Deviance Residuals:

# Min 1Q Median 3Q Max

# -0.8530 -0.8530 -0.4948 -0.4948 2.0789

#

# Coefficients:

# Estimate Std. Error z value Pr(>|z|)

# (Intercept) -2.0386 0.2126 -9.588 < 2e-16 ***

# microchip1 1.2149 0.2489 4.881 1.05e-06 ***

# ---

# Signif. codes: 0 ‘***’ 0.001 ‘**’ 0.01 ‘*’ 0.05 ‘.’ 0.1 ‘ ’ 1

#

# (Dispersion parameter for binomial family taken to be 1)

#

# Null deviance: 528.93 on 498 degrees of freedom

# Residual deviance: 501.92 on 497 degrees of freedom

# AIC: 505.92

#

# Number of Fisher Scoring iterations: 4

chip_rad_ci <- confint(modelchiprad)

round(exp(cbind(modelchiprad$coef, chip_rad_ci)), digits = 2)

# 2.5 % 97.5 %

# (Intercept) 0.13 0.08 0.19

# microchip1 3.37 2.10 5.58

modelchip_rad_RR<-odds_to_rr(modelchiprad)

format(round(modelchip_rad_RR,2),nsmall=2)

# RR lower.ci upper.ci

# (Intercept) 0.16 0.11 0.24

# microchip1 2.21 1.69 2.76

###########desexing and pure/cross

chi.desex.pure_rad<- table(dogadsra_d$desexed, dogadsra_d$pure_cross)

chi.desex.pure_rad

# cross designer pure unknown

# 0 106 7 267 8

# 1 38 4 64 5

chisq.test(chi.desex.pure_rad)

# Pearson's Chi-squared test

#

# data: chi.desex.pure_rad

# X-squared = 6.2941, df = 3, p-value = 0.09815

#

# Warning message:

# In chisq.test(chi.desex.pure_rad) :

# Chi-squared approximation may be incorrect

modelpure_rad <- glm(desexed ~ pure_cross, family = binomial, data = dogadsra_d)

summary(modelpure_rad)

# Call:

# glm(formula = desexed ~ pure_cross, family = binomial, data = dogadsra_d)

#

# Deviance Residuals:

# Min 1Q Median 3Q Max

# -0.9854 -0.6555 -0.6555 -0.6555 1.8129

#

# Coefficients:

# Estimate Std. Error z value Pr(>|z|)

# (Intercept) -1.0259 0.1891 -5.426 5.78e-08 ***

# pure_crossdesigner 0.4662 0.6547 0.712 0.4764

# pure_crosspure -0.4025 0.2348 -1.714 0.0864 .

# pure_crossunknown 0.5558 0.6006 0.925 0.3547

# ---

# Signif. codes: 0 ‘***’ 0.001 ‘**’ 0.01 ‘*’ 0.05 ‘.’ 0.1 ‘ ’ 1

#

# (Dispersion parameter for binomial family taken to be 1)

#

# Null deviance: 528.93 on 498 degrees of freedom

# Residual deviance: 523.02 on 495 degrees of freedom

# AIC: 531.02

#

# Number of Fisher Scoring iterations: 4

pure_rad_ci <- confint(modelpure_rad)

round(exp(cbind(modelpure_rad$coef, pure_rad_ci)), digits = 2)

# 2.5 % 97.5 %

# (Intercept) 0.36 0.24 0.51

# pure_crossdesigner 1.59 0.40 5.59

# pure_crosspure 0.67 0.42 1.07

# pure_crossunknown 1.74 0.50 5.56

modelpure_rad_RR<-odds_to_rr(modelpure_rad)

format(round(modelpure_rad_RR,2),nsmall=2)

# RR lower.ci upper.ci

# (Intercept) 0.42 0.29 0.58

# pure_crossdesigner 1.41 0.46 2.77

# pure_crosspure 0.72 0.49 1.05

# pure_crossunknown 1.50 0.56 2.76

################desexing and state

chistate_rad<- table(dogadsra_d$desexed,dogadsra_d$state)

chistate_rad

# VIC na NSW QLD SA TAS WA

# 0 47 51 121 103 17 16 33

# 1 15 8 44 24 6 6 8

# >

chisq.test(chistate_rad)

# Pearson's Chi-squared test

#

# data: chistate_rad

# X-squared = 6.0922, df = 6, p-value = 0.4129

#

# Warning message:

# In chisq.test(chistate_rad) : Chi-squared approximation may be incorrect

#should i do the or and rr ,or should I put together the states that have deseing as regulation

#and those that dont to make higher counts?

#make new column called 'requiring desexing'

#create bins

dogadsra_d <-

dogadsra_d %>%

mutate(

ID_desex_required = case_when(

state == "SA" ~ "required",

state == "NSW" ~ "not required",

state == "QLD" ~ "required",

state == "VIC" ~ "required",

state == "WA" ~ "not required",

state == "TAS" ~ "not required",

is.na(state) ~ "unclear",

)

)

dogadsra_d

##fix states requirign desex to include na's as - unclear

dogadsra_d$ID_desex_required [is.na (dogadsra_d$ID_desex_required)] <- "unclear"

table(dogadsra_d$ID_desex_required)

chistate_rad2<- table(dogadsra_d$desexed,dogadsra_d$ID_desex_required)

chistate_rad2

# # not required required unclear

# 0 217 120 51

# 1 73 30 8

chisq.test(chistate_rad2)

# Pearson's Chi-squared test

#

# data: chistate_rad2

# X-squared = 4.4473, df = 2, p-value = 0.1082

modelchi_state <- glm(desexed ~ ID_desex_required, family = binomial, data = dogadsra_d)

summary(modelchi_state)

# Call:

# glm(formula = desexed ~ ID_desex_required, family = binomial,

# data = dogadsra_d)

#

# Deviance Residuals:

# Min 1Q Median 3Q Max

# -0.7662 -0.7662 -0.6908 -0.5398 1.9990

#

# Coefficients:

# Estimate Std. Error z value Pr(>|z|)

# (Intercept) -1.0754 0.1521 -7.072 1.53e-12 ***

# ID_desex_requiredrequired -0.2360 0.2266 -1.042 0.2976

# ID_desex_requiredunclear -0.7770 0.4096 -1.897 0.0578 .

# ---

# Signif. codes: 0 ‘***’ 0.001 ‘**’ 0.01 ‘*’ 0.05 ‘.’ 0.1 ‘ ’ 1

#

# (Dispersion parameter for binomial family taken to be 1)

#

# Null deviance: 528.93 on 498 degrees of freedom

# Residual deviance: 524.61 on 496 degrees of freedom

# AIC: 530.61

#

# Number of Fisher Scoring iterations: 4

state_ra_ci <- confint(modelchi_state)

round(exp(cbind(modelchi_state$coef, state_ra_ci)), digits = 2)

# 2.5 % 97.5 %

# (Intercept) 0.34 0.25 0.46

# ID_desex_requiredrequired 0.79 0.50 1.23

# ID_desex_requiredunclear 0.46 0.19 0.98

modelrequire_ra_RR<-odds_to_rr(modelchi_state)

format(round(modelrequire_ra_RR,2),nsmall=2)

#desexing and BREEDER ID (STATE REGISTERED)

chi.stateID.rad<- table(dogadsra_d$desexed, dogadsra_d$state_registered)

chi.stateID.rad

# no yes

# 0 363 25

# 1 108 3

chisq.test(chi.stateID.rad)

# Pearson's Chi-squared test with Yates' continuity correction

#

# data: chi.stateID.rad

# X-squared = 1.6286, df = 1, p-value = 0.2019

#desexing and offered by

chi.offeredby_rad<- table(dogadsra_d$desexed, dogadsra_d$offered_by)

chi.offeredby_rad

# Breeder Owner

# 0 70 318

# 1 9 102

chisq.test(chi.offeredby_rad)

# Pearson's Chi-squared test with Yates' continuity correction

#

# data: chi.offeredby_rad

# X-squared = 5.667, df = 1, p-value = 0.01729

modelseller_rad <- glm(desexed ~ offered_by, family = binomial, data = dogadsra_d)

summary(modelseller_rad)

# Call:

# glm(formula = desexed ~ offered_by, family = binomial, data = dogadsra_d)

#

# Deviance Residuals:

# Min 1Q Median 3Q Max

# -0.7459 -0.7459 -0.7459 -0.4918 2.0843

#

# Coefficients:

# Estimate Std. Error z value Pr(>|z|)

# (Intercept) -2.0513 0.3541 -5.793 6.92e-09 ***

# offered_byOwner 0.9142 0.3719 2.458 0.014 *

# ---

# Signif. codes: 0 ‘***’ 0.001 ‘**’ 0.01 ‘*’ 0.05 ‘.’ 0.1 ‘ ’ 1

#

# (Dispersion parameter for binomial family taken to be 1)

#

# Null deviance: 528.93 on 498 degrees of freedom

# Residual deviance: 521.69 on 497 degrees of freedom

# AIC: 525.69

#

# Number of Fisher Scoring iterations: 4

seller_rad_ci <- confint(modelseller_rad)

round(exp(cbind(modelseller_rad$coef, seller_rad_ci)), digits = 2)

# 2.5 % 97.5 %

# (Intercept) 0.13 0.06 0.24

# offered_byOwner 2.49 1.26 5.52

modelseller_rad_RR<-odds_to_rr(modelseller_rad)

format(round(modelseller_rad_RR,2),nsmall=2)

# RR lower.ci upper.ci

# (Intercept) 0.16 0.08 0.29

# offered_byOwner 1.87 1.19 2.75

##########desexing and vx

chi.vx_rad <- table(dogadsra_d$desexed, dogadsra_d$vaccination)

chi.vx_rad

# no yes

# 0 179 209

# 1 33 78

chisq.test(chi.vx_rad)

# Pearson's Chi-squared test with Yates' continuity correction

#

# data: chi.vx_rad

# X-squared = 8.8455, df = 1, p-value = 0.002938

modelvx_rad <- glm(desexed ~ vaccination, family = binomial, data = dogadsra_d)

summary(modelvx_rad)

# Call:

# glm(formula = desexed ~ vaccination, family = binomial, data = dogadsra_d)

#

# Deviance Residuals:

# Min 1Q Median 3Q Max

# -0.7964 -0.7964 -0.5817 -0.5817 1.9288

#

# Coefficients:

# Estimate Std. Error z value Pr(>|z|)

# (Intercept) -1.6909 0.1894 -8.925 < 2e-16 ***

# vaccinationyes 0.7053 0.2313 3.049 0.00229 **

# ---

# Signif. codes: 0 ‘***’ 0.001 ‘**’ 0.01 ‘*’ 0.05 ‘.’ 0.1 ‘ ’ 1

#

# (Dispersion parameter for binomial family taken to be 1)

#

# Null deviance: 528.93 on 498 degrees of freedom

# Residual deviance: 519.14 on 497 degrees of freedom

# AIC: 523.14

#

# Number of Fisher Scoring iterations: 4

vx_rad_ci <- confint(modelvx_rad)

round(exp(cbind(modelvx_rad$coef, vx_rad_ci)), digits = 2)

# 2.5 % 97.5 %

# (Intercept) 0.18 0.13 0.26

# vaccinationyes 2.02 1.30 3.22

modelvx_rad_RR<-odds_to_rr(modelvx_rad)

format(round(modelvx_rad_RR,2),nsmall=2)

# RR lower.ci upper.ci

# (Intercept) 0.23 0.16 0.31

# vaccinationyes 1.65 1.22 2.15

########desexing and org registered

chi.org_rad <- table(dogadsra_d$desexed, dogadsra_d$breeder_org_affiliation)

chi.org_rad

# none yes

# 0 342 46

# 1 105 6

chisq.test(chi.org_rad)

# Pearson's Chi-squared test with Yates' continuity correction

#

# data: chi.org_rad

# X-squared = 3.1868, df = 1, p-value = 0.07423

#ensure na's are unclear

dogadsra_d$ID_bin [is.na (dogadsra_d$ID_bin)] <- "unclear"

table(dogadsra_d$ID_bin)

chi.idbin_rad<- table(dogadsra_d$desexed, dogadsra_d$ID_bin)

chi.idbin_rad

# not required required unclear

# 0 170 167 51

# 1 58 45 8

chisq.test(chi.idbin_rad)

# Pearson's Chi-squared test

#

# data: chi.idbin_rad

# X-squared = 4.045, df = 2, p-value = 0.1323

#not sig

#######desexing and price bin

chi.price_rad<- table(dogadsra_d$desexed, dogadsra_d$price_bin)

chi.price_rad

# $1 - $500 $2000+ $501 - $2000 Free Unknown

# 0 137 47 118 58 28

# 1 52 6 25 24 4

chisq.test(chi.price_rad)

# Pearson's Chi-squared test

#

# data: chi.price_rad

# X-squared = 12.66, df = 4, p-value = 0.01306

modelprice_rad <- glm(desexed ~ price_bin, family = binomial, data = dogadsra_d)

summary(modelprice_rad)

# Call:

# glm(formula = desexed ~ price_bin, family = binomial, data = dogadsra_d)

#

# Deviance Residuals:

# Min 1Q Median 3Q Max

# -0.8322 -0.8022 -0.6199 -0.4902 2.0874

#

# Coefficients:

# Estimate Std. Error z value Pr(>|z|)

# (Intercept) -0.96874 0.16288 -5.948 2.72e-09 ***

# price_bin$2000+ -1.08965 0.46310 -2.353 0.0186 *

# price_bin$501 - $2000 -0.58307 0.27387 -2.129 0.0333 *

# price_binFree 0.08635 0.29230 0.295 0.7677

# price_binUnknown -0.97717 0.55879 -1.749 0.0803 .

# ---

# Signif. codes: 0 ‘***’ 0.001 ‘**’ 0.01 ‘*’ 0.05 ‘.’ 0.1 ‘ ’ 1

#

# (Dispersion parameter for binomial family taken to be 1)

#

# Null deviance: 528.93 on 498 degrees of freedom

# Residual deviance: 515.62 on 494 degrees of freedom

# AIC: 525.62

#

# Number of Fisher Scoring iterations: 4

price_rad_ci <- confint(modelprice_rad)

round(exp(cbind(modelprice_rad$coef, price_rad_ci)), digits = 2)

# 2.5 % 97.5 %

# (Intercept) 0.38 0.27 0.52

# price_bin$2000+ 0.34 0.12 0.78

# price_bin$501 - $2000 0.56 0.32 0.95

# price_binFree 1.09 0.61 1.92

# price_binUnknown 0.38 0.11 1.02

modelprice_rad_RR<-odds_to_rr(modelprice_rad)

format(round(modelprice_rad_RR,2),nsmall=2)

# RR lower.ci upper.ci

# (Intercept) 0.44 0.33 0.58

# price_bin$2000+ 0.39 0.15 0.82

# price_bin$501 - $2000 0.62 0.38 0.96

# price_binFree 1.07 0.67 1.59

# price_binUnknown 0.44 0.13 1.01

####desexing and age bin

chi.age_rad <- table(dogadsra_d$desexed, dogadsra_d$age_bin)

chi.age_rad

# 1 - 3 years 3 + years 6 months - 1 year Unknown

# 0 146 66 129 47

# 1 48 38 18 7

chisq.test(chi.age_rad)

# Pearson's Chi-squared test

#

# data: chi.age_rad

# X-squared = 24.173, df = 3, p-value = 2.299e-05

modelage_rad <- glm(desexed ~ age_bin, family = binomial, data = dogadsra_d)

summary(modelage_rad)

# Call:

# glm(formula = desexed ~ age_bin, family = binomial, data = dogadsra_d)

#

# Deviance Residuals:

# Min 1Q Median 3Q Max

# -0.9537 -0.7540 -0.5270 -0.5111 2.0494

#

# Coefficients:

# Estimate Std. Error z value Pr(>|z|)

# (Intercept) -1.1124 0.1664 -6.686 2.3e-11 ***

# age_bin3 + years 0.5603 0.2630 2.131 0.03310 *

# age_bin6 months - 1 year -0.8570 0.3016 -2.841 0.00449 **

# age_binUnknown -0.7918 0.4380 -1.808 0.07061 .

# ---

# Signif. codes: 0 ‘***’ 0.001 ‘**’ 0.01 ‘*’ 0.05 ‘.’ 0.1 ‘ ’ 1

#

# (Dispersion parameter for binomial family taken to be 1)

#

# Null deviance: 528.93 on 498 degrees of freedom

# Residual deviance: 504.58 on 495 degrees of freedom

# AIC: 512.58

#

# Number of Fisher Scoring iterations: 4

age_radci <- confint(modelage_rad)

round(exp(cbind(modelage_rad$coef, age_radci)), digits = 2)

# 2.5 % 97.5 %

# (Intercept) 0.33 0.23 0.45

# age_bin3 + years 1.75 1.04 2.93

# age_bin6 months - 1 year 0.42 0.23 0.75

# age_binUnknown 0.45 0.18 1.01

modelage_rad_RR<-odds_to_rr(modelage_rad)

format(round(modelage_rad_RR,2),nsmall=2)

# RR lower.ci upper.ci

# (Intercept) 0.39 0.28 0.51

# age_bin3 + years 1.50 1.03 2.05

# age_bin6 months - 1 year 0.49 0.28 0.80

# age_binUnknown 0.52 0.22 1.01

>

#######################BREEDER ID CHI SQ, RR AND REGRESSION###################

#using a new dataset with only sa qld and vic - states with breeder id required

#shelter and rescue will be in this dataset. na ads also removed.

dogadsID <- dogads3

dogadsID

#select NSW ads and delete rows

dogadsID<- dogadsID[!(dogadsID$state == "NSW"), ]

dogadsID

#575 DELETED from dataset

#select act ads and delete rows

dogadsID<- dogadsID[!(dogadsID$state == "ACT"), ]

dogadsID

#7 DELETED from dataset

#select TAS ads and delete rows

dogadsID<- dogadsID[!(dogadsID$state == "TAS"), ]

dogadsID

#58 DELETED from dataset

#select WA ads and delete rows

dogadsID<- dogadsID[!(dogadsID$state == "WA"), ]

dogadsID

#138 DELETED from dataset

#select NT ads and delete rows

dogadsID<- dogadsID[!(dogadsID$state == "NT"), ]

dogadsID

#20 DELETED from dataset

#select na ads and delete rows

dogadsID<- dogadsID[!(dogadsID$state == "na"), ]

dogadsID

#164 DELETED from dataset

#total 773 ads to work from

#remove ads with unclear state_registered (unclear if breeder ID is included)

#select na ads and delete rows

dogadsID<- dogadsID[!(dogadsID$state_registered == "unclear"), ]

dogadsID

#771 ads remaining to work from

#confirming dataset correct and ready to go

table(dogadsID$state_registered, dogadsID$state)

#now we change the microchip value unclear to "no" as the counts will be too small.

dogadsID$microchip [dogadsID$microchip == "unclear"] <- "no"

table(dogadsID$microchip)

#done.

#now we change the vacc value unclear to "no" as the counts will be too small.

dogadsID$vaccination [dogadsID$vaccination == "unclear"] <- "no"

table(dogadsID$vaccination)

#done. but need to get rid of the factor 'unclear'.

dogadsID <- dogadsID[ dogadsID$vaccination != "unclear", , drop=FALSE]; dogadsID$vaccination <- factor(dogadsID$vaccination); summary(dogadsID)

#done

#change state registered from yes and no to 1 and 0

dogadsID$state_registered [dogadsID$state_registered == "yes"] <- "1"

dogadsID$state_registered [dogadsID$state_registered == "no"] <- "0"

table(dogadsID$state_registered)

#change ANKC, to "breeder org, yes or no" - as numbers and % for ANKC and

#other are almost identical.

#so ankc and Other = "yes"

#none = "no"

#now we change the value ankc to "yes"

dogadsID$ankc_registered [dogadsID$ankc_registered == "ankc"] <- "yes"

#check if the change worked.

table(dogadsID$ankc_registered)

#change value other to "yes"

dogadsID$ankc_registered [dogadsID$ankc_registered == "other"] <- "yes"

#check if the change worked.

table(dogadsID$ankc_registered)

#correct.

#change unclear to no

dogadsID$ankc_registered [dogadsID$ankc_registered == "unclear"] <- "none"

#check if the change worked.

table(dogadsID$ankc_registered)

#correct.

#change column name to "breeder organisation affiliation" as now has ALL org groups not just ankc.

dogadsID <- dogadsID %>%

rename(

"breeder_org_affiliation" = ankc_registered

)

#put price into bins.

dogadsID <-

dogadsID %>%

mutate(

price_bin = case_when(

price == 0 ~ "Free",

price < 501 ~ "$1 - $500",

price < 2001 ~ "$501 - $2000",

price > 2000 ~ "$2000+",

is.na(price) ~ "Unknown",

)

)

table(dogadsID$price_bin)

#done

#age bins amalgamating so 3+

dogadsID <-

dogadsID %>%

mutate(

age_bin = case_when(

age < 8*7 ~ "< 8 weeks old",

age < 6 * 4 * 7 ~ "8 weeks - 6 months",

age < 365 ~ "6 months - 1 year",

age < 3 * 365 ~ "1 - 3 years",

age < 7 * 365 ~ "3 + years",

is.na(age) ~ "Unknown",

TRUE ~ "3 + years"

)

)

table(dogadsID$age_bin)

#done

#need to turn all independant variables into categorical variables.

#microchip

#desexed

#pure/cross

#offered by

#vaccinatin

#breeder org affiliation

#change microchip to categorical

dogadsID$microchip <- factor(dogadsID$microchip)

#check if worked - yes

dogadsID

#change desexed to categorical

dogadsID$desexed <- factor(dogadsID$desexed)

#check if worked - yes

dogadsID

#change pure_cross to categorical

dogadsID$pure_cross <- factor(dogadsID$pure_cross)

#check if worked - yes

dogadsID

#change offered by to categorical

dogadsID$offered_by <- factor(dogadsID$offered_by)

#check if worked - yes

dogadsID

#change vaccination to categorical

dogadsID$vaccination <- factor(dogadsID$vaccination)

#check if worked - yes

dogadsID

#change breeder org to categorical

dogadsID$breeder_org_affiliation <- factor(dogadsID$breeder_org_affiliation)

#check if worked - yes

dogadsID

#change state registered org to categorical

dogadsID$state_registered <- factor(dogadsID$state_registered)

#check if worked - yes

dogadsID

#new dataset for BID univariate and regression

#saved

write_rds(dogadsID,"C:/Users/Sofia Costa/OneDrive/Adelaide University Hons/Honours Program/R/dogadsID.rds")

#load

dogadsID <- read_rds("C:/Users/Sofia Costa/OneDrive/Adelaide University Hons/Honours Program/R/dogadsID.rds")

dogadsID

table(dogadsID$state)

table(dogads3$state)

#########################microchipping

#note we changed unclear to "no" in microchipping as counts are too small.

BID_chip <- table(dogadsID$microchip, dogadsID$state_registered)

BID_chip

# 0 1

# no 116 46

# yes 286 323

chisq.test(BID_chip)

# Pearson's Chi-squared test with Yates' continuity correction

#

# data: BID_chip

# X-squared = 30.16, df = 1, p-value = 3.979e-08

modelBID_chip_ra <- glm(state_registered ~ microchip, family = binomial, data = dogadsID)

summary(modelBID_chip_ra)

# Call:

# glm(formula = state_registered ~ microchip, family = binomial,

# data = dogadsID)

#

# Deviance Residuals:

# Min 1Q Median 3Q Max

# -1.2295 -1.2295 -0.8173 1.1262 1.5868

#

# Coefficients:

# Estimate Std. Error z value Pr(>|z|)

# (Intercept) -0.9249 0.1742 -5.308 1.11e-07 ***

# microchipyes 1.0466 0.1922 5.445 5.19e-08 ***

# ---

# Signif. codes: 0 ‘***’ 0.001 ‘**’ 0.01 ‘*’ 0.05 ‘.’ 0.1 ‘ ’ 1

#

# (Dispersion parameter for binomial family taken to be 1)

#

# Null deviance: 1067.4 on 770 degrees of freedom

# Residual deviance: 1035.3 on 769 degrees of freedom

# AIC: 1039.3

#

# Number of Fisher Scoring iterations: 4

BID_ra_ci <- confint(modelBID_chip_ra)

round(exp(cbind(modelBID_chip_ra$coef, BID_ra_ci)), digits = 2)

# 2.5 % 97.5 %

# (Intercept) 0.40 0.28 0.55

# microchipyes 2.85 1.97 4.18

modelpure_ra_RR<-odds_to_rr(modelBID_chip_ra)

format(round(modelpure_ra_RR,2),nsmall=2)

# RR lower.ci upper.ci

# (Intercept) 0.56 0.43 0.70

# microchipyes 1.51 1.34 1.66

##########vaccinated

BID_vac <- table(dogadsID$vaccination, dogadsID$state_registered)

BID_vac

# 0 1

# no 119 43

# unclear 0 0

# yes 283 326

chisq.test(BID_vac)

# Pearson's Chi-squared test with Yates' continuity correction

#

# data: BID_vac

# X-squared = 36.273, df = 1, p-value = 1.715e-09

modelBID_vac_ra <- glm(state_registered ~ vaccination, family = binomial, data = dogadsID)

summary(modelBID_vac_ra)

# Call:

# glm(formula = state_registered ~ vaccination, family = binomial,

# data = dogadsID)

#

# Deviance Residuals:

# Min 1Q Median 3Q Max

# -1.2380 -1.2380 -0.7855 1.1180 1.6287

#

# Coefficients:

# Estimate Std. Error z value Pr(>|z|)

# (Intercept) -1.0179 0.1779 -5.721 1.06e-08 ***

# vaccinationyes 1.1594 0.1956 5.927 3.08e-09 ***

# ---

# Signif. codes: 0 ‘***’ 0.001 ‘**’ 0.01 ‘*’ 0.05 ‘.’ 0.1 ‘ ’ 1

#

# (Dispersion parameter for binomial family taken to be 1)

#

# Null deviance: 1067.4 on 770 degrees of freedom

# Residual deviance: 1028.7 on 769 degrees of freedom

# AIC: 1032.7

#

# Number of Fisher Scoring iterations: 4

BID_vac_ci <- confint(modelBID_vac_ra)

round(exp(cbind(modelBID_vac_ra$coef, BID_vac_ci)), digits = 2)

# 2.5 % 97.5 %

# (Intercept) 0.36 0.25 0.51

# vaccinationyes 3.19 2.19 4.72

modelvac_ra_RR<-odds_to_rr(modelBID_vac_ra)

format(round(modelvac_ra_RR,2),nsmall=2)

# RR lower.ci upper.ci

# (Intercept) 0.52 0.39 0.66

# vaccinationyes 1.56 1.39 1.70

#########pure _cross

BID_pure <- table(dogadsID$pure_cross, dogadsID$state_registered)

BID_pure

# 0 1

# cross 121 99

# designer 21 45

# pure 249 223

# unknown 11 2

chisq.test(BID_pure)

# Pearson's Chi-squared test

#

# data: BID_pure

# X-squared = 17.209, df = 3, p-value = 0.00064

#

modelBID_pure_ra <- glm(state_registered ~ pure_cross, family = binomial, data = dogadsID)

summary(modelBID_pure_ra)

# Call:

# glm(formula = state_registered ~ pure_cross, family = binomial,

# data = dogadsID)

#

# Deviance Residuals:

# Min 1Q Median 3Q Max

# -1.513 -1.131 -1.093 1.225 1.935

#

# Coefficients:

# Estimate Std. Error z value Pr(>|z|)

# (Intercept) -0.20067 0.13552 -1.481 0.13867

# pure_crossdesigner 0.96281 0.29700 3.242 0.00119 **

# pure_crosspure 0.09039 0.16391 0.551 0.58131

# pure_crossunknown -1.50408 0.78056 -1.927 0.05399 .

# ---

# Signif. codes: 0 ‘***’ 0.001 ‘**’ 0.01 ‘*’ 0.05 ‘.’ 0.1 ‘ ’ 1

#

# (Dispersion parameter for binomial family taken to be 1)

#

# Null deviance: 1067.4 on 770 degrees of freedom

# Residual deviance: 1049.4 on 767 degrees of freedom

# AIC: 1057.4

#

# Number of Fisher Scoring iterations: 4

BID_pure_ci <- confint(modelBID_pure_ra)

round(exp(cbind(modelBID_pure_ra$coef, BID_pure_ci)), digits = 2)

# 2.5 % 97.5 %

# (Intercept) 0.82 0.63 1.07

# pure_crossd0.06 0.92esigner 2.62 1.48 4.76

# pure_crosspure 1.09 0.79 1.51

# pure_crossunknown 0.22 0.03 0.85

modelpure_ra_RR<-odds_to_rr(modelBID_pure_ra)

format(round(modelpure_ra_RR,2),nsmall=2)

# # RR lower.ci upper.ci

# (Intercept) 0.90 0.76 1.03

# pure_crossdesigner 1.48 1.20 1.70

# pure_crosspure 1.05 0.88 1.21

# pure_crossunknown 0.35 0.06 0.92

########seller (offered by)

BID_seller <- table(dogadsID$offered_by, dogadsID$state_registered)

BID_seller

# 0 1

# Breeder 138 261

# Owner 252 85

# Shelter/Rescue 12 23

chisq.test(BID_seller)

# Pearson's Chi-squared test

#

# data: BID_seller

# X-squared = 122.94, df = 2, p-value < 2.2e-16

modelBID_seller_ra <- glm(state_registered ~ offered_by, family = binomial, data = dogadsID)

summary(modelBID_seller_ra)

# Call:

# glm(formula = state_registered ~ offered_by, family = binomial,

# data = dogadsID)

#

# Deviance Residuals:

# Min 1Q Median 3Q Max

# -1.4632 -0.7624 -0.7624 0.9214 1.6598

#

# Coefficients:

# Estimate Std. Error z value Pr(>|z|)

# (Intercept) 0.63727 0.10525 6.055 1.41e-09 ***

# offered_byOwner -1.72404 0.16374 -10.529 < 2e-16 ***

# offered_byShelter/Rescue 0.01332 0.37133 0.036 0.971

# ---

# Signif. codes: 0 ‘***’ 0.001 ‘**’ 0.01 ‘*’ 0.05 ‘.’ 0.1 ‘ ’ 1

#

# (Dispersion parameter for binomial family taken to be 1)

#

# Null deviance: 1067.42 on 770 degrees of freedom

# Residual deviance: 940.25 on 768 degrees of freedom

# AIC: 946.25

#

# Number of Fisher Scoring iterations: 4

BID_seller_ci <- confint(modelBID_seller_ra)

round(exp(cbind(modelBID_seller_ra$coef, BID_seller_ci)), digits = 2)

# 2.5 % 97.5 %

# (Intercept) 1.89 1.54 2.33

# offered_byOwner 0.18 0.13 0.24

# offered_byShelter/Rescue 1.01 0.50 2.16

modelseller_ra_RR<-odds_to_rr(modelBID_seller_ra)

format(round(modelseller_ra_RR,2),nsmall=2)

# RR lower.ci upper.ci

# (Intercept) 1.33 1.22 1.42

# offered_byOwner 0.29 0.22 0.38

# offered_byShelter/Rescue 1.01 0.66 1.39

#############breeder org affiliation

BID_org <- table(dogadsID$breeder_org_affiliation, dogadsID$state_registered)

BID_org

# 0 1

# none 300 234

# yes 102 135

chisq.test(BID_org)

#

# Pearson's Chi-squared test with Yates' continuity correction

#

# data: BID_org

# X-squared = 10.84, df = 1, p-value = 0.0009933

modelBID_org_ra <- glm(state_registered ~ breeder_org_affiliation, family = binomial, data = dogadsID)

summary(modelBID_org_ra)

# Call:

# glm(formula = state_registered ~ breeder_org_affiliation, family = binomial,

# data = dogadsID)

#

# Deviance Residuals:

# Min 1Q Median 3Q Max

# -1.299 -1.074 -1.074 1.285 1.285

#

# Coefficients:

# Estimate Std. Error z value Pr(>|z|)

# (Intercept) -0.24846 0.08722 -2.849 0.004388 **

# breeder_org_affiliationyes 0.52876 0.15754 3.356 0.000789 ***

# ---

# Signif. codes: 0 ‘***’ 0.001 ‘**’ 0.01 ‘*’ 0.05 ‘.’ 0.1 ‘ ’ 1

#

# (Dispersion parameter for binomial family taken to be 1)

#

# Null deviance: 1067.4 on 770 degrees of freedom

# Residual deviance: 1056.0 on 769 degrees of freedom

# AIC: 1060

#

# Number of Fisher Scoring iterations: 3

BID_org_ci <- confint(modelBID_org_ra)

round(exp(cbind(modelBID_org_ra$coef, BID_org_ci)), digits = 2)

# 2.5 % 97.5 %

# (Intercept) 0.78 0.66 0.92

# breeder_org_affiliationyes 1.70 1.25 2.31

modelorg_ra_RR<-odds_to_rr(modelBID_org_ra)

format(round(modelorg_ra_RR,2),nsmall=2)

# RR lower.ci upper.ci

# (Intercept) 0.87 0.79 0.96

# breeder_org_affiliationyes 1.27 1.12 1.42

##############age

BID_age <- table(dogadsID$age_bin, dogadsID$state_registered)

BID_age

#

# 0 1

# < 8 weeks old 80 196

# 1 - 3 years 79 16

# 3 + years 45 8

# 6 months - 1 year 43 14

# 8 weeks - 6 months 133 125

# Unknown 22 10

chisq.test(BID_age)

# Pearson's Chi-squared test

#

# data: BID_age

# X-squared = 134.7, df = 5, p-value < 2.2e-16

modelBID_age_ra <- glm(state_registered ~ age_bin, family = binomial, data = dogadsID)

summary(modelBID_age_ra)

# Call:

# glm(formula = state_registered ~ age_bin, family = binomial,

# data = dogadsID)

#

# Deviance Residuals:

# Min 1Q Median 3Q Max

# -1.5738 -1.1512 -0.5721 0.8274 1.9447

#

# Coefficients:

# Estimate Std. Error z value Pr(>|z|)

# (Intercept) 0.8961 0.1327 6.754 1.44e-11 ***

# age_bin1 - 3 years -2.4929 0.3046 -8.185 2.72e-16 ***

# age_bin3 + years -2.6233 0.4060 -6.462 1.04e-10 ***

# age_bin6 months - 1 year -2.0182 0.3351 -6.023 1.71e-09 ***

# age_bin8 weeks - 6 months -0.9581 0.1820 -5.265 1.40e-07 ***

# age_binUnknown -1.6845 0.4038 -4.172 3.02e-05 ***

# ---

# Signif. codes: 0 ‘***’ 0.001 ‘**’ 0.01 ‘*’ 0.05 ‘.’ 0.1 ‘ ’ 1

#

# (Dispersion parameter for binomial family taken to be 1)

#

# Null deviance: 1067.42 on 770 degrees of freedom

# Residual deviance: 924.15 on 765 degrees of freedom

# AIC: 936.15

#

# Number of Fisher Scoring iterations: 4

BID_age_ci <- confint(modelBID_age_ra)

round(exp(cbind(modelBID_age_ra$coef, BID_age_ci)), digits = 2)

# (Intercept) 2.45 1.90 3.19

# age_bin1 - 3 years 0.08 0.04 0.15

# age_bin3 + years 0.07 0.03 0.15

# age_bin6 months - 1 year 0.13 0.07 0.25

# age_bin8 weeks - 6 months 0.38 0.27 0.55

# age_binUnknown 0.19 0.08 0.40

modelage_ra_RR<-odds_to_rr(modelBID_age_ra)

format(round(modelage_ra_RR,2),nsmall=2)

# RR lower.ci upper.ci

# (Intercept) 1.45 1.33 1.56

# age_bin1 - 3 years 0.15 0.08 0.25

# age_bin3 + years 0.13 0.06 0.26

# age_bin6 months - 1 year 0.23 0.12 0.39

# age_bin8 weeks - 6 months 0.54 0.41 0.70

# age_binUnknown 0.30 0.14 0.56

###############price

BID_price <- table(dogadsID$price_bin, dogadsID$state_registered)

BID_price

# 0 1

# $1 - $500 135 97

# $2000+ 86 100

# $501 - $2000 123 163

# Free 40 2

# Unknown 18 7

chisq.test(BID_price)

# Pearson's Chi-squared test

#

# data: BID_price

# X-squared = 50.774, df = 4, p-value = 2.489e-10

modelBID_price_ra <- glm(state_registered ~ price_bin, family = binomial, data = dogadsID)

summary(modelBID_price_ra)

# Call:

# glm(formula = state_registered ~ price_bin, family = binomial,

# data = dogadsID)

#

# Deviance Residuals:

# Min 1Q Median 3Q Max

# -1.2991 -1.2421 -0.3124 1.1141 2.4676

#

# Coefficients:

# Estimate Std. Error z value Pr(>|z|)

# (Intercept) -0.3306 0.1331 -2.483 0.013010 *

# price_bin$2000+ 0.4814 0.1984 2.427 0.015229 *

# price_bin$501 - $2000 0.6121 0.1788 3.423 0.000620 ***

# price_binFree -2.6652 0.7366 -3.618 0.000297 ***

# price_binUnknown -0.6139 0.4649 -1.321 0.186667

# ---

# Signif. codes: 0 ‘***’ 0.001 ‘**’ 0.01 ‘*’ 0.05 ‘.’ 0.1 ‘ ’ 1

#

# (Dispersion parameter for binomial family taken to be 1)

#

# Null deviance: 1067.4 on 770 degrees of freedom

# Residual deviance: 1008.8 on 766 degrees of freedom

# AIC: 1018.8

#

# Number of Fisher Scoring iterations: 5

BID_price_ci <- confint(modelBID_price_ra)

round(exp(cbind(modelBID_price_ra$coef, BID_price_ci)), digits = 2)

# 2.5 % 97.5 %

# (Intercept) 0.72 0.55 0.93

# price_bin$2000+ 1.62 1.10 2.39

# price_bin$501 - $2000 1.84 1.30 2.62

# price_binFree 0.07 0.01 0.23

# price_binUnknown 0.54 0.20 1.30

modelprice_ra_RR<-odds_to_rr(modelBID_price_ra)

format(round(modelprice_ra_RR,2),nsmall=2)

# RR lower.ci upper.ci

# (Intercept) 0.83 0.70 0.96

# price_bin$2000+ 1.25 1.05 1.44

# price_bin$501 - $2000 1.31 1.14 1.48

# price_binFree 0.13 0.02 0.37

# price_binUnknown 0.69 0.33 1.13

##################prior to sale - microchip regulation - is it significant/predictor??################

#with the dataset dogadsra with the below modifications.

#first create new bin with states reuire prior to sale, and not prior to sale. territories

#are excluded as per previously.

dogadsra <-

dogadsra %>%

mutate(

m_prior_bin = case_when(

state == "SA" ~ "prior",

state == "NSW" ~ "prior",

state == "QLD" ~ "prior",

state == "VIC" ~ "prior",

state == "WA" ~ "not prior",

state == "TAS" ~ "not prior",

is.na(state) ~ "unclear",

)

)

table(dogadsra$m_prior_bin)

#model 13 with the added microchip prior bin but with state removed

#because its the same factor essentially.

model_ra_microchip14 <- glm(microchip ~ vaccination + state_registered + offered_by +

price_bin + breeder_org_affiliation + m_prior_bin, family = binomial,

data = dogadsra)

summary(model_ra_microchip14)

# Call:

# glm(formula = microchip ~ vaccination + state_registered + offered_by +

# price_bin + breeder_org_affiliation + m_prior_bin, family = binomial,

# data = dogadsra)

#

# Deviance Residuals:

# Min 1Q Median 3Q Max

# -2.7888 -0.3907 0.3161 0.4685 2.2850

#

# Coefficients:

# Estimate Std. Error z value Pr(>|z|)

# (Intercept) -2.1201 0.3547 -5.977 2.27e-09 ***

# vaccinationyes 3.4098 0.1804 18.905 < 2e-16 ***

# state_registeredyes 0.2989 0.2448 1.221 0.222087

# offered_byOwner -0.4141 0.2393 -1.731 0.083514 .

# price_bin$2000+ 1.2108 0.3251 3.725 0.000195 ***

# price_bin$501 - $2000 0.6133 0.2129 2.881 0.003964 **

# price_binFree -0.3220 0.3319 -0.970 0.331942

# price_binUnknown 1.0142 0.3972 2.553 0.010670 *

# breeder_org_affiliationyes 0.4033 0.2919 1.381 0.167162

# m_prior_binprior 0.6654 0.2354 2.827 0.004700 **

# ---

# Signif. codes: 0 ‘***’ 0.001 ‘**’ 0.01 ‘*’ 0.05 ‘.’ 0.1 ‘ ’ 1

#

# (Dispersion parameter for binomial family taken to be 1)

#

# Null deviance: 1733.03 on 1498 degrees of freedom

# Residual deviance: 978.16 on 1489 degrees of freedom

# (163 observations deleted due to missingness)

# AIC: 998.16

#

# Number of Fisher Scoring iterations: 5

#significant but cant include this becuase its the same as "states". Will do univariate analysis instead, like desexing.

#will chat to lora about this.

#univariate analysis - na;s not included for this univariate.

#delete all na's

dogadsra_prior <- dogadsra

#select na ads and delete rows

dogadsra_prior<- dogadsra_prior[!(dogadsra_prior$state == "na"), ]

dogadsra_prior

#change price_bin to categorical

dogadsra_prior$m_prior_bin <- factor(dogadsra_prior$m_prior_bin)

#check if worked - yes

dogadsra_prior

chip_prior <- table(dogadsra_prior$m_prior_bin, dogadsra_prior$microchip)

chip_prior

# 0 1

# not prior 81 112

# prior 316 990

# 0 1

# 0 81 112

# 1 316 990

chisq.test(chip_prior)

# Pearson's Chi-squared test with Yates' continuity correction

#

# data: chip_prior

# X-squared = 26.375, df = 1, p-value = 2.812e-07

#m_prior needs to be 1 and 0

#change m_prior from yes and no to 1 and 0

dogadsra_prior$m_prior_bin [ dogadsra_prior$m_prior_bin == "prior"] <- "1"

dogadsra_prior$m_prior_bin [ dogadsra_prior$m_prior_bin == "not prior"] <- "0"

#worked

model_chip_prior_ra <- glm(m_prior_bin ~ microchip, family = binomial, data = dogadsra_prior)

summary(model_chip_prior_ra)

# Call:

# glm(formula = m_prior_bin ~ microchip, family = binomial, data = dogadsra_prior)

#

# Deviance Residuals:

# Min 1Q Median 3Q Max

# -2.1384 0.4630 0.4630 0.4630 0.6756

#

# Coefficients:

# Estimate Std. Error z value Pr(>|z|)

# (Intercept) 1.3613 0.1245 10.931 < 2e-16 ***

# microchip1 0.8179 0.1595 5.127 2.94e-07 ***

# ---

# Signif. codes: 0 ‘***’ 0.001 ‘**’ 0.01 ‘*’ 0.05 ‘.’ 0.1 ‘ ’ 1

#

# (Dispersion parameter for binomial family taken to be 1)

#

# Null deviance: 1151.3 on 1498 degrees of freedom

# Residual deviance: 1126.1 on 1497 degrees of freedom

# AIC: 1130.1

#

# Number of Fisher Scoring iterations: 4

prior_ra_ci <- confint(model_chip_prior_ra)

round(exp(cbind(model_chip_prior_ra$coef, prior_ra_ci)), digits = 2)

# 2.5 % 97.5 %

# (Intercept) 3.90 3.07 5.01

# microchip1 2.27 1.65 3.09

modelprior_ra_RR<-odds_to_rr(model_chip_prior_ra)

format(round(modelprior_ra_RR,2),nsmall=2)

# RR lower.ci upper.ci

# (Intercept) 1.11 1.10 1.11

# microchip1 1.08 1.05 1.10

table(dogads3$state_registered)

##############NEW NOV REGRESSION ANALYSIS - MICROCHIP###########################

#new dataset for microchippinng regression (dogads36_11)

dogads3chip <- dogads3

dogads3chip

#1735 rows

#we don't want to include ACT oR NT as these dont have enough data. we also need to combine

#the age bins 3-7 and up for the same reason.

#also want to move all 'unclears' to 'no'

#remove NT

#select just nt

dogads3chip<- dogads3chip[!(dogads3chip$state == "NT"), ]

dogads3chip

#this has removed 20 nt rows. new row number #1715

#remove act

dogads3chip<- dogads3chip[!(dogads3chip$state == "ACT"), ]

dogads3chip

#removed 7 act ads. Now 1708 rows.

table(dogads3chip$state)

#correct

#just check how many ads in total are in each group/bin

count(dogads3chip$age_bin)

dogads3chip

#this tells us that there are 18 ads in 7+ and 3-7 has 95.

#so combined they should have 113 as in the 3+ group

#combine age bins 3-7 and 7 up

dogads3chip <-

dogads3chip %>%

mutate(

age_bin = case_when(

age < 8*7 ~ "< 8 weeks old",

age < 6 * 4 * 7 ~ "8 weeks - 6 months",

age < 365 ~ "6 months - 1 year",

age < 3 * 365 ~ "1 - 3 years",

age < 7 * 365 ~ "3 + years",

is.na(age) ~ "Unknown",

TRUE ~ "3 + years"

)

)

table(dogads3chip$age_bin)

#correct. there is now a new bin of 3+ with 113 ads

##now we need to save this particular dataframe.

#we wil then use excel to remove the unclears in micorchip, vacc and vaccination to all No's. and desex and chip to 1 an 0.

write.csv(dogads3chip, file = "dogads3chip.csv", row.names = TRUE)

#and then import that dataframe back into r.

#load dataset

dogads3chip <- read_csv("C:/Users/Sofia Costa/OneDrive/Adelaide University Hons/Honours Program/R/dogads3chip.csv")

dogads3chip

#change ANKC, to "breeder org, yes or no" - as numbers and % for ANKC and

#other are almost identical.

#Remove all shelter and rescue ads from the dataframe as this is likely confounding

#factors plus the count is too small.

#Change victoria to a yes - under the Stte requiring Breeder ID column.

#as they do require, even if they dont need to post number in ad.

#change ankc column so that none is reference

#bring back the na's in for state BID required so all totals equal the same

#change the price bins so count isnt so small

#then re-run the regression analysis.

#change ANKC, to "breeder org, yes or no" - as numbers and % for ANKC and

#other are almost identical.

#so ankc and Other = "yes"

#none = "no"

#check how many we will be changing

table(dogads3chip$ankc_registered)

#now we change the value ankc to "yes"

dogads3chip$ankc_registered [dogads3chip$ankc_registered == "ankc"] <- "yes"

#check if the change worked.

table(dogads3chip$ankc_registered)

#change value other to "yes"

dogads3chip$ankc_registered [dogads3chip$ankc_registered == "other"] <- "yes"

#check if the change worked.

table(dogads3chip$ankc_registered)

#correct.

#change column name to "breeder organisation affiliation)

dogads3chip <- dogads3chip %>%

rename(

"breeder_org_affiliation" = ankc_registered

)

dogads3chip

#Remove all shelter and rescue ads from the dataframe as this is likely confounding

#factors plus the count is too small.

#remove all shelter/rescue ads

#see how many we need to remove

table(dogads3chip$offered_by)

#we need to remove 46

#select shelter/rescue and delete rows

dogads3chip<- dogads3chip[!(dogads3chip$offered_by == "Shelter/Rescue"), ]

dogads3chip

#worked. Our total number of rows is now 1662

table(dogads3chip$offered_by)

############create the price bins

table(dogads3chip$price_bin)

dogads3chip <-

dogads3chip %>%

mutate(

price_bin = case_when(

price == 0 ~ "Free",

price < 501 ~ "$1 - $500",

price < 2001 ~ "$501 - $2000",

price > 2000 ~ "$2000+",

is.na(price) ~ "Unknown",

)

)

table(dogads3chip$price_bin)

dogads3chip

#make all variables factors

#change state to categorical

dogads3chip$state<- factor(dogads3chip$state)

#check if worked - yes

dogads3chip

#change desexed to categorical

dogads3chip$desexed<- factor(dogads3chip$desexed)

#check if worked - yes

dogads3chip

#change microchip to categorical

dogads3chip$microchip<- factor(dogads3chip$microchip)

#check if worked - yes

dogads3chip

#change vaccination to categorical

dogads3chip$vaccination<- factor(dogads3chip$vaccination)

#check if worked - yes

dogads3chip

#change pure)cross to categorical

dogads3chip$pure_cross<- factor(dogads3chip$pure_cross)

#check if worked - yes

dogads3chip

#change offered by to categorical

dogads3chip$offered_by<- factor(dogads3chip$offered_by)

#check if worked - yes

dogads3chip

#change age_bin to categorical

dogads3chip$age_bin <- factor(dogads3chip$age_bin)

#check if worked - yes

dogads3chip

#change id_bin to categorical

dogads3chip$ID_bin <- factor(dogads3chip$ID_bin)

#check if worked - yes

dogads3chip

#change breeder org affil to categorical

dogads3chip$breeder_org_affiliation <- factor(dogads3chip$breeder_org_affiliation)

#check if worked - yes

dogads3chip

#change ankc column so that none is reference (now, the breeder_org)affiliation)

dogads3chip$breeder_org_affiliation <- relevel(dogads3chip$breeder_org_affiliation, ref= "none")

table(dogads3chip$breeder_org_affiliation)

#change STATE column so that VIC is reference

dogads3chip$state <- relevel(dogads3chip$state, ref= "VIC")

table(dogads3chip$state)

#change unclear in breeder org affilition to "no" in excel.

#Data is now ready for univariate analysis and regression

write.csv(dogads3chip, file = "dogads3chip.csv", row.names = TRUE)

#load dataset

dogads3chip <- read_csv("C:/Users/Sofia Costa/OneDrive/Adelaide University Hons/Honours Program/R/dogads3chip.csv")

dogads3chip

#######nov univariate analysis MICROCHIP###################

#make a contigency table with the two factors you want

##microchip and pure/cross

chi.microchip.pure_ra <- table(dogads3chip$microchip, dogads3chip$pure_cross)

chi.microchip.pure_ra

# cross designer pure unknown

# 0 194 11 270 13

# 1 254 111 800 9

# >

chisq.test(chi.microchip.pure_ra)

# Pearson's Chi-squared test

#

# data: chi.microchip.pure_ra

# X-squared = 84.499, df = 3, p-value < 2.2e-16

modelpure_ra <- glm(microchip ~ pure_cross, family = binomial, data = dogads3chip)

summary(modelpure_ra)

# Call:

# glm(formula = microchip ~ pure_cross, family = binomial, data = dogadsra)

#

# Deviance Residuals:

# Min 1Q Median 3Q Max

# -2.1937 -1.2938 0.7626 0.7626 1.3370

#

# Coefficients:

# Estimate Std. Error z value Pr(>|z|)

# (Intercept) 0.26948 0.09535 2.826 0.00471 **

# pure_crossdesigner 2.04216 0.33010 6.186 6.15e-10 ***

# pure_crosspure 0.81671 0.11851 6.891 5.53e-12 ***

# pure_crossunknown -0.63720 0.44399 -1.435 0.15124

# ---

# Signif. codes: 0 ‘***’ 0.001 ‘**’ 0.01 ‘*’ 0.05 ‘.’ 0.1 ‘ ’ 1

#

# (Dispersion parameter for binomial family taken to be 1)

#

# Null deviance: 2012.2 on 1661 degrees of freedom

# Residual deviance: 1925.5 on 1658 degrees of freedom

# AIC: 1933.5

#

# Number of Fisher Scoring iterations: 4

pure_ra_ci <- confint(modelpure_ra)

round(exp(cbind(modelpure_ra$coef, pure_ra_ci)), digits = 2)

# 2.5 % 97.5 %

# (Intercept) 1.31 1.09 1.58

# pure_crossdesigner 7.71 4.21 15.55

# pure_crosspure 2.26 1.79 2.86

# pure_crossunknown 0.53 0.21 1.25

# >

modelpure_ra_RR<-odds_to_rr(modelpure_ra)

format(round(modelpure_ra_RR,2),nsmall=2)

# RR lower.ci upper.ci

# (Intercept) 1.07 1.02 1.12

# pure_crossdesigner 1.34 1.29 1.38

# pure_crosspure 1.20 1.15 1.24

# pure_crossunknown 0.79 0.48 1.06

####################microchip and state

chi.microchip.statera <- table(dogads3chip$microchip, dogads3chip$state)

chi.microchip.statera

# VIC na NSW QLD SA TAS WA

# 0 13 91 158 115 30 29 52

# 1 163 72 410 350 67 29 83

chisq.test(chi.microchip.statera)

# Pearson's Chi-squared test

#

# data: chi.microchip.statera

# X-squared = 118.97, df = 6, p-value < 2.2e-16

modelstatera <- glm(microchip ~ state, family = binomial, data = dogads3chip)

summary(modelstatera)

# Call:

# glm(formula = microchip ~ state, family = binomial, data = dogadsra)

#

# Deviance Residuals:

# Min 1Q Median 3Q Max

# -2.2828 -1.0797 0.7538 0.8074 1.2784

#

# Coefficients:

# Estimate Std. Error z value Pr(>|z|)

# (Intercept) 2.5288 0.2882 8.775 < 2e-16 ***

# statena -2.7630 0.3285 -8.410 < 2e-16 ***

# stateNSW -1.5752 0.3030 -5.198 2.01e-07 ***

# stateQLD -1.4158 0.3076 -4.603 4.17e-06 ***

# stateSA -1.7253 0.3624 -4.761 1.93e-06 ***

# stateTAS -2.5288 0.3899 -6.486 8.83e-11 ***

# stateWA -2.0612 0.3381 -6.096 1.09e-09 ***

# ---

# Signif. codes: 0 ‘***’ 0.001 ‘**’ 0.01 ‘*’ 0.05 ‘.’ 0.1 ‘ ’ 1

#

# (Dispersion parameter for binomial family taken to be 1)

#

# Null deviance: 2012.2 on 1661 degrees of freedom

# Residual deviance: 1888.7 on 1655 degrees of freedom

# AIC: 1902.7

#

# Number of Fisher Scoring iterations: 5

stateraci <- confint(modelstatera)

round(exp(cbind(modelstatera$coef, stateraci)), digits = 2)

# 2.5 % 97.5 %

# (Intercept) 12.54 7.43 23.20

# statena 0.06 0.03 0.12

# stateNSW 0.21 0.11 0.36

# stateQLD 0.24 0.13 0.43

# stateSA 0.18 0.08 0.36

# stateTAS 0.08 0.04 0.17

# stateWA 0.13 0.06 0.24

modelstate_ra_RR<-odds_to_rr(modelstatera)

format(round(modelstate_ra_RR,2),nsmall=2)

# RR lower.ci upper.ci

# (Intercept) 1.37 1.34 1.39

# statena 0.19 0.10 0.31

# stateNSW 0.47 0.29 0.66

# stateQLD 0.52 0.33 0.72

# stateSA 0.42 0.24 0.65

# stateTAS 0.23 0.11 0.41

# stateWA 0.33 0.19 0.52

############################microchip and offered by

chi.microchip.offeredby_ra <- table(dogads3chip$microchip, dogads3chip$offered_by)

chi.microchip.offeredby_ra

# Breeder Owner

# 0 75 413

# 1 632 542

chisq.test(chi.microchip.offeredby_ra)

# Pearson's Chi-squared test with Yates' continuity correction

#

# data: chi.microchip.offeredby_ra

# X-squared = 207.08, df = 1, p-value < 2.2e-16

#or and rr

modeloffered_ra <- glm(microchip ~ offered_by, family = binomial, data = dogads3chip)

summary(modeloffered_ra)

# Call:

# glm(formula = microchip ~ offered_by, family = binomial, data = dogadsra)

#

# Deviance Residuals:

# Min 1Q Median 3Q Max

# -2.1183 -1.2948 0.4736 1.0644 1.0644

#

# Coefficients:

# Estimate Std. Error z value Pr(>|z|)

# (Intercept) 2.1314 0.1221 17.45 <2e-16 ***

# offered_byOwner -1.8596 0.1385 -13.43 <2e-16 ***

# ---

# Signif. codes: 0 ‘***’ 0.001 ‘**’ 0.01 ‘*’ 0.05 ‘.’ 0.1 ‘ ’ 1

#

# (Dispersion parameter for binomial family taken to be 1)

#

# Null deviance: 2012.2 on 1661 degrees of freedom

# Residual deviance: 1784.7 on 1660 degrees of freedom

# AIC: 1788.7

#

# Number of Fisher Scoring iterations: 4

offfered_ra_ci <- confint(modeloffered_ra)

round(exp(cbind(modeloffered_ra$coef, offfered_ra_ci)), digits = 2)

#

# 2.5 % 97.5 %

# (Intercept) 8.43 6.68 10.79

# offered_byOwner 0.16 0.12 0.20

modeloffered_ra_RR<-odds_to_rr(modeloffered_ra)

format(round(modeloffered_ra_RR,2),nsmall=2)

# RR lower.ci upper.ci

# (Intercept) 1.35 1.33 1.36

# offered_byOwner 0.39 0.31 0.46

>

########################microchip and vaccination

chi.microchip.vx_ra <- table(dogads3chip$microchip, dogads3chip$vaccination)

chi.microchip.vx_ra

# no yes

# 0 354 134

# 1 74 1100

chisq.test(chi.microchip.vx_ra)

# Pearson's Chi-squared test with Yates' continuity correction

#

# data: chi.microchip.vx_ra

# X-squared = 787.53, df = 1, p-value < 2.2e-16

modelvx_ra <- glm(microchip ~ vaccination, family = binomial, data = dogads3chip)

summary(modelvx_ra)

# Call:

# glm(formula = microchip ~ vaccination, family = binomial, data = dogadsra)

#

# Deviance Residuals:

# Min 1Q Median 3Q Max

# -2.1072 -0.6162 0.4795 0.4795 1.8735

#

# Coefficients:

# Estimate Std. Error z value Pr(>|z|)

# (Intercept) -1.5652 0.1278 -12.24 <2e-16 ***

# vaccinationyes 3.6705 0.1572 23.35 <2e-16 ***

# ---

# Signif. codes: 0 ‘***’ 0.001 ‘**’ 0.01 ‘*’ 0.05 ‘.’ 0.1 ‘ ’ 1

#

# (Dispersion parameter for binomial family taken to be 1)

#

# Null deviance: 2012.2 on 1661 degrees of freedom

# Residual deviance: 1242.0 on 1660 degrees of freedom

# AIC: 1246

#

# Number of Fisher Scoring iterations: 4

vx_ra_ci <- confint(modelvx_ra)

round(exp(cbind(modelvx_ra$coef, vx_ra_ci)), digits = 2)

# 2.5 % 97.5 %

# (Intercept) 0.21 0.16 0.27

# vaccinationyes 39.27 29.02 53.77

modelvx_ra_RR<-odds_to_rr(modelvx_ra)

format(round(modelvx_ra_RR,2),nsmall=2)

# RR lower.ci upper.ci

# (Intercept) 0.47 0.40 0.55

# vaccinationyes 1.40 1.40 1.40

################microchip and breeder org affiliation

chi.microchip.ankc_ra <- table(dogads3chip$microchip, dogads3chip$breeder_org_affiliation)

chi.microchip.ankc_ra

# none yes

# 0 450 38

# 1 727 447

chisq.test(chi.microchip.ankc_ra)

# Pearson's Chi-squared test with Yates' continuity correction

#

# data: chi.microchip.ankc_ra

# X-squared = 151.56, df = 1, p-value < 2.2e-16

#

modelaffiliation_ra <- glm(microchip ~ breeder_org_affiliation, family = binomial, data = dogads3chip)

summary(modelaffiliation_ra)

# # Call:

# glm(formula = microchip ~ breeder_org_affiliation, family = binomial,

# data = dogads3chip)

#

# Deviance Residuals:

# Min 1Q Median 3Q Max

# -2.2568 -1.3867 0.4040 0.9816 0.9816

#

# Coefficients:

# Estimate Std. Error z value Pr(>|z|)

# (Intercept) 0.47968 0.05998 7.997 1.27e-15 ***

# breeder_org_affiliationyes 1.98529 0.17931 11.072 < 2e-16 ***

# ---

# Signif. codes: 0 ‘***’ 0.001 ‘**’ 0.01 ‘*’ 0.05 ‘.’ 0.1 ‘ ’ 1

#

# (Dispersion parameter for binomial family taken to be 1)

#

# Null deviance: 2012.2 on 1661 degrees of freedom

# Residual deviance: 1832.3 on 1660 degrees of freedom

# AIC: 1836.3

#

# Number of Fisher Scoring iterations: 5

affiliation_ra_ci <- confint(modelaffiliation_ra)

round(exp(cbind(modelaffiliation_ra$coef, affiliation_ra_ci)), digits = 2)

# 2.5 % 97.5 %

# (Intercept) 1.62 1.44 1.82

# breeder_org_affiliationyes 7.28 5.19 10.50

modelaffiliation_ra_RR<-odds_to_rr(modelaffiliation_ra)

format(round(modelaffiliation_ra_RR,2),nsmall=2)

# RR lower.ci upper.ci

# (Intercept) 1.12 1.10 1.15

# breeder_org_affiliationyes 1.34 1.31 1.36

##microchip and age

chi.microchip.age_ra <- table(dogads3chip$microchip, dogads3chip$age_bin)

chi.microchip.age_ra

# < 8 weeks old 1 - 3 years 3 + years 6 months - 1 year 8 weeks - 6 months Unknown

# 0 131 91 49 62 140 15

# 1 459 103 55 85 433 39

chisq.test(chi.microchip.age_ra)

# Pearson's Chi-squared test

#

# data: chi.microchip.age_ra

# X-squared = 77.592, df = 5, p-value = 2.675e-15

modelage_ra <- glm(microchip ~ age_bin, family = binomial, data = dogads3chip)

summary(modelage_ra)

# Call:

# glm(formula = microchip ~ age_bin, family = binomial, data = dogadsra)

#

# Deviance Residuals:

# Min 1Q Median 3Q Max

# -1.7349 -1.2305 0.7086 0.7485 1.1288

#

# Coefficients:

# Estimate Std. Error z value Pr(>|z|)

# (Intercept) 1.25385 0.09906 12.658 < 2e-16 ***

# age_bin1 - 3 years -1.12998 0.17467 -6.469 9.85e-11 ***

# age_bin3 + years -1.13834 0.22001 -5.174 2.29e-07 ***

# age_bin6 months - 1 year -0.93834 0.19418 -4.832 1.35e-06 ***

# age_bin8 weeks - 6 months -0.12476 0.13880 -0.899 0.369

# age_binUnknown -0.29834 0.31956 -0.934 0.351

# ---

# Signif. codes: 0 ‘***’ 0.001 ‘**’ 0.01 ‘*’ 0.05 ‘.’ 0.1 ‘ ’ 1

#

# (Dispersion parameter for binomial family taken to be 1)

#

# Null deviance: 2012.2 on 1661 degrees of freedom

# Residual deviance: 1938.0 on 1656 degrees of freedom

# AIC: 1950

#

# Number of Fisher Scoring iterations: 4

age_ra_ci <- confint(modelage_ra)

round(exp(cbind(modelage_ra $coef, age_ra_ci)), digits = 2)

# 2.5 % 97.5 %

# (Intercept) 3.50 2.90 4.27

# age_bin1 - 3 years 0.32 0.23 0.45

# age_bin3 + years 0.32 0.21 0.49

# age_bin6 months - 1 year 0.39 0.27 0.57

# age_bin8 weeks - 6 months 0.88 0.67 1.16

# age_binUnknown 0.74 0.40 1.43

modelage_ra_RR<-odds_to_rr(modelage_ra)

format(round(modelage_ra_RR,2),nsmall=2)

#

# RR lower.ci upper.ci

# (Intercept) 1.27 1.24 1.29

# age_bin1 - 3 years 0.62 0.50 0.74

# age_bin3 + years 0.62 0.47 0.77

# age_bin6 months - 1 year 0.69 0.55 0.82

# age_bin8 weeks - 6 months 0.96 0.87 1.04

# age_binUnknown 0.91 0.70 1.10

###########################chi sq test for price

chi.microchip.price_ra<- table(dogads3chip$microchip, dogads3chip$price_bin)

chi.microchip.price_ra

# $1 - $500 $2000+ $501 - $2000 Free Unknown

# 0 247 32 122 60 27

# 1 311 324 455 36 48

chisq.test(chi.microchip.price_ra)

# Pearson's Chi-squared test

#

# data: chi.microchip.price_ra

# X-squared = 202.21, df = 4, p-value < 2.2e-16

#

modelprice_ra <- glm(microchip ~ price_bin, family = binomial, data = dogads3chip)

summary(modelprice_ra)

# Call:

# glm(formula = microchip ~ price_bin, family = binomial, data = dogadsra)

#

# Deviance Residuals:

# Min 1Q Median 3Q Max

# -2.1951 -1.2767 0.6893 0.6893 1.4006

#

# Coefficients:

# Estimate Std. Error z value Pr(>|z|)

# (Intercept) 0.23040 0.08523 2.703 0.00686 **

# price_bin$2000+ 2.08460 0.20392 10.222 < 2e-16 ***

# price_bin$501 - $2000 1.08587 0.13289 8.171 3.05e-16 ***

# price_binFree -0.74123 0.22739 -3.260 0.00112 **

# price_binUnknown 0.34496 0.25521 1.352 0.17649

# ---

# Signif. codes: 0 ‘***’ 0.001 ‘**’ 0.01 ‘*’ 0.05 ‘.’ 0.1 ‘ ’ 1

#

# (Dispersion parameter for binomial family taken to be 1)

#

# Null deviance: 2012.2 on 1661 degrees of freedom

# Residual deviance: 1801.7 on 1657 degrees of freedom

# AIC: 1811.7

#

# Number of Fisher Scoring iterations: 4

price_ra_ci <- confint(modelprice_ra )

round(exp(cbind(modelprice_ra $coef, price_ra_ci)), digits = 2)

# 2.5 % 97.5 %

# (Intercept) 1.26 1.07 1.49

# price_bin$2000+ 8.04 5.47 12.19

# price_bin$501 - $2000 2.96 2.29 3.85

# price_binFree 0.48 0.30 0.74

# price_binUnknown 1.41 0.86 2.35

modelprice_ra_RR<-odds_to_rr(modelprice_ra )

format(round(modelprice_ra_RR,2),nsmall=2)

#

# RR lower.ci upper.ci

# (Intercept) 1.06 1.02 1.11

# price_bin$2000+ 1.35 1.32 1.37

# price_bin$501 - $2000 1.24 1.20 1.28

# price_binFree 0.76 0.60 0.91

# price_binUnknown 1.09 0.96 1.20

#####################REGRESSION MODEL - MIRCOCHIP ###################

#WITH ALL VARIABLES

model_ra_microchip <- glm(microchip ~ state + vaccination +

pure_cross + offered_by + breeder_org_affiliation +

age_bin + price_bin, family = binomial,

data = dogads3chip)

summary(model_ra_microchip)

# Call:

# glm(formula = microchip ~ state + vaccination + pure_cross +

# offered_by + breeder_org_affiliation + age_bin + price_bin,

# family = binomial, data = dogads3chip)

#

# Deviance Residuals:

# Min 1Q Median 3Q Max

# -3.1417 -0.3546 0.2878 0.5147 2.5206

#

# Coefficients:

# Estimate Std. Error z value Pr(>|z|)

# (Intercept) 0.81382 0.45535 1.787 0.07390 .

# statena -3.06533 0.42850 -7.154 8.45e-13 ***

# stateNSW -2.23407 0.38945 -5.736 9.67e-09 ***

# stateQLD -2.10995 0.39686 -5.317 1.06e-07 ***

# stateSA -2.87249 0.48287 -5.949 2.70e-09 ***

# stateTAS -3.03472 0.52845 -5.743 9.32e-09 ***

# stateWA -2.61600 0.45491 -5.751 8.89e-09 ***

# vaccinationyes 3.61391 0.18770 19.254 < 2e-16 ***

# pure_crossdesigner 0.70339 0.43537 1.616 0.10618

# pure_crosspure 0.20469 0.18744 1.092 0.27483

# pure_crossunknown 0.07058 0.70245 0.100 0.91997

# offered_byOwner -0.62030 0.22556 -2.750 0.00596 **

# breeder_org_affiliationyes 0.47657 0.28330 1.682 0.09254 .

# age_bin1 - 3 years -0.26234 0.26318 -0.997 0.31886

# age_bin3 + years 0.66146 0.35374 1.870 0.06150 .

# age_bin6 months - 1 year -0.23654 0.29039 -0.815 0.41533

# age_bin8 weeks - 6 months -0.24683 0.20149 -1.225 0.22058

# age_binUnknown -0.07430 0.47973 -0.155 0.87692

# price_bin$2000+ 0.70157 0.32645 2.149 0.03163 *

# price_bin$501 - $2000 0.19458 0.20915 0.930 0.35220

# price_binFree -0.93504 0.35401 -2.641 0.00826 **

# price_binUnknown 0.53099 0.37289 1.424 0.15445

# ---

# Signif. codes: 0 ‘***’ 0.001 ‘**’ 0.01 ‘*’ 0.05 ‘.’ 0.1 ‘ ’ 1

#

# (Dispersion parameter for binomial family taken to be 1)

#

# Null deviance: 2012.2 on 1661 degrees of freedom

# Residual deviance: 1065.1 on 1640 degrees of freedom

# AIC: 1109.1

#

# Number of Fisher Scoring iterations: 6

microchip_ra_dci <- confint(model_ra_microchip)

round(exp(cbind(model_ra_microchip$coef, microchip_ra_dci)), digits = 2)

# 2.5 % 97.5 %

# 2.5 % 97.5 %

# (Intercept) 2.26 0.94 5.64

# statena 0.05 0.02 0.11

# stateNSW 0.11 0.05 0.22

# stateQLD 0.12 0.05 0.26

# stateSA 0.06 0.02 0.14

# stateTAS 0.05 0.02 0.13

# stateWA 0.07 0.03 0.17

# vaccinationyes 37.11 25.93 54.17

# pure_crossdesigner 2.02 0.89 4.97

# pure_crosspure 1.23 0.85 1.77

# pure_crossunknown 1.07 0.28 4.29

# offered_byOwner 0.54 0.34 0.83

# breeder_org_affiliationyes 1.61 0.93 2.83

# age_bin1 - 3 years 0.77 0.46 1.29

# age_bin3 + years 1.94 0.97 3.88

# age_bin6 months - 1 year 0.79 0.45 1.40

# age_bin8 weeks - 6 months 0.78 0.53 1.16

# age_binUnknown 0.93 0.37 2.45

# price_bin$2000+ 2.02 1.07 3.87

# price_bin$501 - $2000 1.21 0.81 1.83

# price_binFree 0.39 0.20 0.79

# price_binUnknown 1.70 0.83 3.57

#model 2: model1 without state

model_ra_microchip2 <- glm(microchip ~ vaccination +

pure_cross + offered_by + breeder_org_affiliation +

age_bin + price_bin, family = binomial,

data = dogads3chip)

summary(model_ra_microchip2)

# Call:

# glm(formula = microchip ~ vaccination + pure_cross + offered_by +

# breeder_org_affiliation + age_bin + price_bin, family = binomial,

# data = dogads3chip)

#

# Deviance Residuals:

# Min 1Q Median 3Q Max

# -2.8948 -0.4604 0.3177 0.5377 2.4471

#

# Coefficients:

# Estimate Std. Error z value Pr(>|z|)

# (Intercept) -1.40319 0.28138 -4.987 6.14e-07 ***

# vaccinationyes 3.45300 0.17232 20.039 < 2e-16 ***

# pure_crossdesigner 0.86377 0.42788 2.019 0.04352 *

# pure_crosspure 0.23173 0.17711 1.308 0.19073

# pure_crossunknown -0.17975 0.68974 -0.261 0.79439

# offered_byOwner -0.73580 0.21524 -3.419 0.00063 ***

# breeder_org_affiliationyes 0.36450 0.27375 1.332 0.18302

# age_bin1 - 3 years -0.05202 0.25386 -0.205 0.83763

# age_bin3 + years 0.76530 0.33359 2.294 0.02178 *

# age_bin6 months - 1 year -0.17362 0.28403 -0.611 0.54101

# age_bin8 weeks - 6 months -0.17659 0.19338 -0.913 0.36115

# age_binUnknown 0.31233 0.45918 0.680 0.49639

# price_bin$2000+ 0.89675 0.31659 2.832 0.00462 **

# price_bin$501 - $2000 0.31524 0.19992 1.577 0.11483

# price_binFree -0.63028 0.33335 -1.891 0.05866 .

# price_binUnknown 0.63019 0.35673 1.767 0.07730 .

# ---

# Signif. codes: 0 ‘***’ 0.001 ‘**’ 0.01 ‘*’ 0.05 ‘.’ 0.1 ‘ ’ 1

#

# (Dispersion parameter for binomial family taken to be 1)

#

# Null deviance: 2012.2 on 1661 degrees of freedom

# Residual deviance: 1140.4 on 1646 degrees of freedom

# AIC: 1172.4

#

# Number of Fisher Scoring iterations: 5

microchip_ra_dci2 <- confint(model_ra_microchip2)

round(exp(cbind(model_ra_microchip2$coef, microchip_ra_dci2)), digits = 2)

# 2.5 % 97.5 %

# (Intercept) 0.25 0.14 0.42

# vaccinationyes 31.60 22.70 44.63

# pure_crossdesigner 2.37 1.06 5.73

# pure_crosspure 1.26 0.89 1.78

# pure_crossunknown 0.84 0.22 3.24

# offered_byOwner 0.48 0.31 0.73

# breeder_org_affiliationyes 1.44 0.85 2.48

# age_bin1 - 3 years 0.95 0.58 1.57

# age_bin3 + years 2.15 1.12 4.14

# age_bin6 months - 1 year 0.84 0.48 1.48

# age_bin8 weeks - 6 months 0.84 0.57 1.22

# age_binUnknown 1.37 0.57 3.45

# price_bin$2000+ 2.45 1.33 4.60

# price_bin$501 - $2000 1.37 0.93 2.03

# price_binFree 0.53 0.28 1.02

# price_binUnknown 1.88 0.94 3.80

library(lmtest)

lrtest(model_ra_microchip, model_ra_microchip2)

# Likelihood ratio test

#

# Model 1: microchip ~ state + vaccination + pure_cross + offered_by + breeder_org_affiliation +

# age_bin + price_bin

# Model 2: microchip ~ vaccination + pure_cross + offered_by + breeder_org_affiliation +

# age_bin + price_bin

# #Df LogLik Df Chisq Pr(>Chisq)

# 1 22 -532.53

# 2 16 -570.22 -6 75.379 3.207e-14 ***

# ---

# Signif. codes: 0 ‘***’ 0.001 ‘**’ 0.01 ‘*’ 0.05 ‘.’ 0.1 ‘ ’ 1

#state is sig

#model2 : model1 without vx

model_ra_microchip3 <- glm(microchip ~ state +

pure_cross + offered_by + breeder_org_affiliation +

age_bin + price_bin, family = binomial,

data = dogads3chip)

summary(model_ra_microchip3)

# Call:

# glm(formula = microchip ~ state + pure_cross + offered_by + breeder_org_affiliation +

# age_bin + price_bin, family = binomial, data = dogads3chip)

#

# Deviance Residuals:

# Min 1Q Median 3Q Max

# -2.6899 -0.9239 0.4084 0.7845 1.9247

#

# Coefficients:

# Estimate Std. Error z value Pr(>|z|)

# (Intercept) 2.87818 0.37683 7.638 2.21e-14 ***

# statena -2.64198 0.35429 -7.457 8.85e-14 ***

# stateNSW -1.63902 0.32608 -5.026 5.00e-07 ***

# stateQLD -1.63645 0.33211 -4.927 8.33e-07 ***

# stateSA -2.14926 0.39770 -5.404 6.51e-08 ***

# stateTAS -2.38237 0.42420 -5.616 1.95e-08 ***

# stateWA -2.07849 0.36879 -5.636 1.74e-08 ***

# pure_crossdesigner 0.97253 0.36844 2.640 0.00830 **

# pure_crosspure 0.31956 0.14122 2.263 0.02365 *

# pure_crossunknown -0.09416 0.51261 -0.184 0.85426

# offered_byOwner -0.91519 0.18027 -5.077 3.84e-07 ***

# breeder_org_affiliationyes 0.65373 0.23326 2.803 0.00507 **

# age_bin1 - 3 years -0.59846 0.20013 -2.990 0.00279 **

# age_bin3 + years -0.37948 0.26179 -1.450 0.14718

# age_bin6 months - 1 year -0.39420 0.21979 -1.793 0.07289 .

# age_bin8 weeks - 6 months -0.14930 0.15591 -0.958 0.33828

# age_binUnknown -0.22723 0.35975 -0.632 0.52762

# price_bin$2000+ 0.72267 0.25832 2.798 0.00515 **

# price_bin$501 - $2000 0.37708 0.15926 2.368 0.01790 *

# price_binFree -0.72373 0.26305 -2.751 0.00594 **

# price_binUnknown 0.22269 0.27496 0.810 0.41799

# ---

# Signif. codes: 0 ‘***’ 0.001 ‘**’ 0.01 ‘*’ 0.05 ‘.’ 0.1 ‘ ’ 1

#

# (Dispersion parameter for binomial family taken to be 1)

#

# Null deviance: 2012.2 on 1661 degrees of freedom

# Residual deviance: 1608.6 on 1641 degrees of freedom

# AIC: 1650.6

#

# Number of Fisher Scoring iterations: 5

microchip_ra_dci3 <- confint(model_ra_microchip3)

round(exp(cbind(model_ra_microchip3$coef, microchip_ra_dci3)), digits = 2)

#

# 2.5 % 97.5 %

# (Intercept) 17.78 8.74 38.52

# statena 0.07 0.03 0.14

# stateNSW 0.19 0.10 0.36

# stateQLD 0.19 0.10 0.36

# stateSA 0.12 0.05 0.25

# stateTAS 0.09 0.04 0.21

# stateWA 0.13 0.06 0.25

# pure_crossdesigner 2.64 1.33 5.70

# pure_crosspure 1.38 1.04 1.82

# pure_crossunknown 0.91 0.32 2.45

# offered_byOwner 0.40 0.28 0.57

# breeder_org_affiliationyes 1.92 1.22 3.06

# age_bin1 - 3 years 0.55 0.37 0.81

# age_bin3 + years 0.68 0.41 1.15

# age_bin6 months - 1 year 0.67 0.44 1.04

# age_bin8 weeks - 6 months 0.86 0.63 1.17

# age_binUnknown 0.80 0.40 1.65

# price_bin$2000+ 2.06 1.25 3.45

# price_bin$501 - $2000 1.46 1.07 1.99

# price_binFree 0.48 0.29 0.81

# price_binUnknown 1.25 0.73 2.16

lrtest(model_ra_microchip, model_ra_microchip3)

# Likelihood ratio test

#

# Model 1: microchip ~ state + vaccination + pure_cross + offered_by + breeder_org_affiliation +

# age_bin + price_bin

# Model 2: microchip ~ state + pure_cross + offered_by + breeder_org_affiliation +

# age_bin + price_bin

# #Df LogLik Df Chisq Pr(>Chisq)

# 1 22 -532.53

# 2 21 -804.31 -1 543.55 < 2.2e-16 ***

# ---

# Signif. codes: 0 ‘***’ 0.001 ‘**’ 0.01 ‘*’ 0.05 ‘.’ 0.1 ‘ ’ 1

#vx is SIGNIFICANT

#moedl 4: model 1 minus pure_cross

model_ra_microchip4 <- glm(microchip ~ state + vaccination +

offered_by + breeder_org_affiliation +

age_bin + price_bin, family = binomial,

data = dogads3chip)

summary(model_ra_microchip4)

# Call:

# glm(formula = microchip ~ state + vaccination + offered_by +

# breeder_org_affiliation + age_bin + price_bin, family = binomial,

# data = dogads3chip)

#

# Deviance Residuals:

# Min 1Q Median 3Q Max

# -3.1771 -0.3456 0.2990 0.5148 2.4842

#

# Coefficients:

# Estimate Std. Error z value Pr(>|z|)

# (Intercept) 0.94381 0.44407 2.125 0.03355 *

# statena -3.11369 0.42874 -7.262 3.80e-13 ***

# stateNSW -2.26077 0.38960 -5.803 6.52e-09 ***

# stateQLD -2.16436 0.39555 -5.472 4.45e-08 ***

# stateSA -2.90549 0.48225 -6.025 1.69e-09 ***

# stateTAS -3.05227 0.52808 -5.780 7.47e-09 ***

# stateWA -2.68739 0.45302 -5.932 2.99e-09 ***

# vaccinationyes 3.62761 0.18751 19.346 < 2e-16 ***

# offered_byOwner -0.61809 0.22519 -2.745 0.00606 **

# breeder_org_affiliationyes 0.48663 0.28187 1.726 0.08426 .

# age_bin1 - 3 years -0.25079 0.25992 -0.965 0.33460

# age_bin3 + years 0.66539 0.35263 1.887 0.05917 .

# age_bin6 months - 1 year -0.24161 0.28973 -0.834 0.40433

# age_bin8 weeks - 6 months -0.23805 0.20076 -1.186 0.23572

# age_binUnknown -0.08047 0.47702 -0.169 0.86604

# price_bin$2000+ 0.85132 0.31397 2.711 0.00670 **

# price_bin$501 - $2000 0.28812 0.20012 1.440 0.14993

# price_binFree -0.91508 0.35140 -2.604 0.00921 **

# price_binUnknown 0.60429 0.36869 1.639 0.10121

# ---

# Signif. codes: 0 ‘***’ 0.001 ‘**’ 0.01 ‘*’ 0.05 ‘.’ 0.1 ‘ ’ 1

#

# (Dispersion parameter for binomial family taken to be 1)

#

# Null deviance: 2012.2 on 1661 degrees of freedom

# Residual deviance: 1068.2 on 1643 degrees of freedom

# AIC: 1106.2

#

# Number of Fisher Scoring iterations: 6

microchip_ra_dci4 <- confint(model_ra_microchip4)

round(exp(cbind(model_ra_microchip4$coef, microchip_ra_dci4)), digits = 2)

#

# 2.5 % 97.5 %

# (Intercept) 2.57 1.10 6.29

# statena 0.04 0.02 0.10

# stateNSW 0.10 0.05 0.22

# stateQLD 0.11 0.05 0.24

# stateSA 0.05 0.02 0.14

# stateTAS 0.05 0.02 0.13

# stateWA 0.07 0.03 0.16

# vaccinationyes 37.62 26.29 54.89

# offered_byOwner 0.54 0.35 0.84

# breeder_org_affiliationyes 1.63 0.94 2.85

# age_bin1 - 3 years 0.78 0.47 1.30

# age_bin3 + years 1.95 0.98 3.89

# age_bin6 months - 1 year 0.79 0.45 1.39

# age_bin8 weeks - 6 months 0.79 0.53 1.17

# age_binUnknown 0.92 0.37 2.43

# price_bin$2000+ 2.34 1.28 4.39

# price_bin$501 - $2000 1.33 0.90 1.98

# price_binFree 0.40 0.20 0.80

# price_binUnknown 1.83 0.90 3.81

lrtest(model_ra_microchip, model_ra_microchip4)

# Likelihood ratio test

#

# Model 1: microchip ~ state + vaccination + pure_cross + offered_by + breeder_org_affiliation +

# age_bin + price_bin

# Model 2: microchip ~ state + vaccination + offered_by + breeder_org_affiliation +

# age_bin + price_bin

# #Df LogLik Df Chisq Pr(>Chisq)

# 1 22 -532.53

# 2 19 -534.08 -3 3.0913 0.3778

#PURE_cROSS NONSIG

#moedl 5: model 4 minus offered by

model_ra_microchip5 <- glm(microchip ~ state + vaccination +

breeder_org_affiliation +

age_bin + price_bin, family = binomial,

data = dogads3chip)

summary(model_ra_microchip5)

# Call:

# glm(formula = microchip ~ state + vaccination + breeder_org_affiliation +

# age_bin + price_bin, family = binomial, data = dogads3chip)

#

# Deviance Residuals:

# Min 1Q Median 3Q Max

# -3.3309 -0.3555 0.3064 0.5159 2.5130

#

# Coefficients:

# Estimate Std. Error z value Pr(>|z|)

# (Intercept) 0.4460 0.4052 1.101 0.271035

# statena -3.2157 0.4287 -7.501 6.34e-14 ***

# stateNSW -2.3143 0.3900 -5.935 2.94e-09 ***

# stateQLD -2.1204 0.3955 -5.362 8.25e-08 ***

# stateSA -2.8201 0.4770 -5.912 3.37e-09 ***

# stateTAS -3.1197 0.5291 -5.896 3.73e-09 ***

# stateWA -2.7619 0.4526 -6.102 1.05e-09 ***

# vaccinationyes 3.6656 0.1870 19.606 < 2e-16 ***

# breeder_org_affiliationyes 0.7501 0.2619 2.864 0.004186 **

# age_bin1 - 3 years -0.3444 0.2573 -1.339 0.180697

# age_bin3 + years 0.5718 0.3531 1.620 0.105290

# age_bin6 months - 1 year -0.3748 0.2865 -1.308 0.190813

# age_bin8 weeks - 6 months -0.2444 0.1997 -1.224 0.221124

# age_binUnknown -0.1314 0.4674 -0.281 0.778540

# price_bin$2000+ 1.0261 0.3065 3.348 0.000813 ***

# price_bin$501 - $2000 0.3967 0.1960 2.024 0.042962 *

# price_binFree -0.9265 0.3519 -2.633 0.008469 **

# price_binUnknown 0.6194 0.3720 1.665 0.095877 .

# ---

# Signif. codes: 0 ‘***’ 0.001 ‘**’ 0.01 ‘*’ 0.05 ‘.’ 0.1 ‘ ’ 1

#

# (Dispersion parameter for binomial family taken to be 1)

#

# Null deviance: 2012.2 on 1661 degrees of freedom

# Residual deviance: 1075.8 on 1644 degrees of freedom

# AIC: 1111.8

#

# Number of Fisher Scoring iterations: 6

microchip_ra_dci5 <- confint(model_ra_microchip5)

round(exp(cbind(model_ra_microchip5$coef, microchip_ra_dci5)), digits = 2)

# 2.5 % 97.5 %

# (Intercept) 1.56 0.72 3.55

# statena 0.04 0.02 0.09

# stateNSW 0.10 0.04 0.21

# stateQLD 0.12 0.05 0.25

# stateSA 0.06 0.02 0.15

# stateTAS 0.04 0.02 0.12

# stateWA 0.06 0.03 0.15

# vaccinationyes 39.08 27.34 56.96

# breeder_org_affiliationyes 2.12 1.28 3.57

# age_bin1 - 3 years 0.71 0.43 1.18

# age_bin3 + years 1.77 0.89 3.54

# age_bin6 months - 1 year 0.69 0.39 1.21

# age_bin8 weeks - 6 months 0.78 0.53 1.16

# age_binUnknown 0.88 0.36 2.26

# price_bin$2000+ 2.79 1.55 5.15

# price_bin$501 - $2000 1.49 1.01 2.19

# price_binFree 0.40 0.20 0.79

# price_binUnknown 1.86 0.91 3.89

lrtest(model_ra_microchip4, model_ra_microchip5)

# Likelihood ratio test

#

# Model 1: microchip ~ state + vaccination + offered_by + breeder_org_affiliation +

# age_bin + price_bin

# Model 2: microchip ~ state + vaccination + breeder_org_affiliation + age_bin +

# price_bin

# #Df LogLik Df Chisq Pr(>Chisq)

# 1 19 -534.08

# 2 18 -537.92 -1 7.6941 0.00554 **

# ---

# Signif. codes: 0 ‘***’ 0.001 ‘**’ 0.01 ‘*’ 0.05 ‘.’ 0.1 ‘ ’

#offered by is SIG

#model 6: model 4 minus breeder org

model_ra_microchip6 <- glm(microchip ~ state + vaccination +

offered_by +

age_bin + price_bin, family = binomial,

data = dogads3chip)

summary(model_ra_microchip6)

# #Call:

# glm(formula = microchip ~ state + vaccination + offered_by +

# age_bin + price_bin, family = binomial, data = dogads3chip)

#

# Deviance Residuals:

# Min 1Q Median 3Q Max

# -3.1069 -0.3419 0.3270 0.5258 2.4989

#

# Coefficients:

# Estimate Std. Error z value Pr(>|z|)

# (Intercept) 1.03205 0.44286 2.330 0.019784 *

# statena -3.08017 0.42877 -7.184 6.78e-13 ***

# stateNSW -2.20976 0.38827 -5.691 1.26e-08 ***

# stateQLD -2.14703 0.39554 -5.428 5.70e-08 ***

# stateSA -2.91266 0.48298 -6.031 1.63e-09 ***

# stateTAS -3.00309 0.52653 -5.704 1.17e-08 ***

# stateWA -2.64300 0.45145 -5.854 4.79e-09 ***

# vaccinationyes 3.64532 0.18741 19.451 < 2e-16 ***

# offered_byOwner -0.76234 0.21136 -3.607 0.000310 ***

# age_bin1 - 3 years -0.26661 0.26001 -1.025 0.305173

# age_bin3 + years 0.67877 0.35360 1.920 0.054908 .

# age_bin6 months - 1 year -0.27306 0.28969 -0.943 0.345884

# age_bin8 weeks - 6 months -0.23091 0.20017 -1.154 0.248686

# age_binUnknown -0.09331 0.47932 -0.195 0.845648

# price_bin$2000+ 1.05752 0.29297 3.610 0.000307 ***

# price_bin$501 - $2000 0.37180 0.19543 1.902 0.057109 .

# price_binFree -0.90493 0.35250 -2.567 0.010254 *

# price_binUnknown 0.61206 0.36839 1.661 0.096626 .

# ---

# Signif. codes: 0 ‘***’ 0.001 ‘**’ 0.01 ‘*’ 0.05 ‘.’ 0.1 ‘ ’ 1

#

# (Dispersion parameter for binomial family taken to be 1)

#

# Null deviance: 2012.2 on 1661 degrees of freedom

# Residual deviance: 1071.2 on 1644 degrees of freedom

# AIC: 1107.2

#

# Number of Fisher Scoring iterations: 6

microchip_ra_dci6 <- confint(model_ra_microchip6)

round(exp(cbind(model_ra_microchip6$coef, microchip_ra_dci6)), digits = 2)

# 2.5 % 97.5 %

# (Intercept) 2.81 1.20 6.86

# statena 0.05 0.02 0.10

# stateNSW 0.11 0.05 0.23

# stateQLD 0.12 0.05 0.25

# stateSA 0.05 0.02 0.14

# stateTAS 0.05 0.02 0.14

# stateWA 0.07 0.03 0.17

# vaccinationyes 38.30 26.77 55.86

# offered_byOwner 0.47 0.31 0.70

# age_bin1 - 3 years 0.77 0.46 1.28

# age_bin3 + years 1.97 0.99 3.95

# age_bin6 months - 1 year 0.76 0.43 1.35

# age_bin8 weeks - 6 months 0.79 0.54 1.17

# age_binUnknown 0.91 0.37 2.41

# price_bin$2000+ 2.88 1.64 5.18

# price_bin$501 - $2000 1.45 0.99 2.13

# price_binFree 0.40 0.20 0.81

# price_binUnknown 1.84 0.91 3.84

lrtest(model_ra_microchip4, model_ra_microchip6)

# Likelihood ratio test

# Model 1: microchip ~ state + vaccination + offered_by + breeder_org_affiliation +

# age_bin + price_bin

# Model 2: microchip ~ state + vaccination + offered_by + age_bin + price_bin

# #Df LogLik Df Chisq Pr(>Chisq)

# 1 19 -534.08

# 2 18 -535.59 -1 3.0335 0.08156 .

# ---

# Signif. codes: 0 ‘***’ 0.001 ‘**’ 0.01 ‘*’ 0.05 ‘.’ 0.1 ‘ ’ 1

#model 7: model 6 minus age

model_ra_microchip7 <- glm(microchip ~ state + vaccination +

offered_by +

price_bin, family = binomial,

data = dogads3chip)

summary(model_ra_microchip7)

# Call:

# glm(formula = microchip ~ state + vaccination + offered_by +

# price_bin, family = binomial, data = dogads3chip)

#

# Deviance Residuals:

# Min 1Q Median 3Q Max

# -3.1228 -0.3658 0.3449 0.5223 2.4280

#

# Coefficients:

# Estimate Std. Error z value Pr(>|z|)

# (Intercept) 0.9450 0.4223 2.238 0.025244 *

# statena -3.0629 0.4231 -7.238 4.54e-13 ***

# stateNSW -2.1694 0.3828 -5.667 1.45e-08 ***

# stateQLD -2.0763 0.3903 -5.320 1.04e-07 ***

# stateSA -2.8406 0.4781 -5.942 2.82e-09 ***

# stateTAS -2.9701 0.5207 -5.704 1.17e-08 ***

# stateWA -2.5803 0.4455 -5.792 6.97e-09 ***

# vaccinationyes 3.5689 0.1802 19.800 < 2e-16 ***

# offered_byOwner -0.7758 0.2045 -3.793 0.000149 ***

# price_bin$2000+ 1.0311 0.2891 3.567 0.000362 ***

# price_bin$501 - $2000 0.3544 0.1941 1.826 0.067858 .

# price_binFree -0.6705 0.3302 -2.031 0.042273 *

# price_binUnknown 0.6573 0.3657 1.797 0.072296 .

# ---

# Signif. codes: 0 ‘***’ 0.001 ‘**’ 0.01 ‘*’ 0.05 ‘.’ 0.1 ‘ ’ 1

#

# (Dispersion parameter for binomial family taken to be 1)

#

# Null deviance: 2012.2 on 1661 degrees of freedom

# Residual deviance: 1079.9 on 1649 degrees of freedom

# AIC: 1105.9

#

# Number of Fisher Scoring iterations: 6

microchip_ra_dci7 <- confint(model_ra_microchip7)

round(exp(cbind(model_ra_microchip7$coef, microchip_ra_dci7)), digits = 2)

# 2.5 % 97.5 %

# (Intercept) 2.57 1.15 6.05

# statena 0.05 0.02 0.10

# stateNSW 0.11 0.05 0.24

# stateQLD 0.13 0.06 0.26

# stateSA 0.06 0.02 0.15

# stateTAS 0.05 0.02 0.14

# stateWA 0.08 0.03 0.18

# vaccinationyes 35.48 25.12 50.97

# offered_byOwner 0.46 0.31 0.69

# price_bin$2000+ 2.80 1.61 5.00

# price_bin$501 - $2000 1.43 0.97 2.09

# price_binFree 0.51 0.27 0.98

# price_binUnknown 1.93 0.95 3.99

lrtest(model_ra_microchip7, model_ra_microchip6)

# Likelihood ratio test

#

# Model 1: microchip ~ state + vaccination + offered_by + price_bin

# Model 2: microchip ~ state + vaccination + offered_by + age_bin + price_bin

# #Df LogLik Df Chisq Pr(>Chisq)

# 1 13 -539.93

# 2 18 -535.59 5 8.6817 0.1225

#not sig

#model 8: model 7 minus age

model_ra_microchip8 <- glm(microchip ~ state + vaccination +

offered_by, family = binomial,

data = dogads3chip)

summary(model_ra_microchip8)

# Call:

# glm(formula = microchip ~ state + vaccination + offered_by, family = binomial,

# data = dogads3chip)

#

# Deviance Residuals:

# Min 1Q Median 3Q Max

# -3.2080 -0.3561 0.3197 0.5562 2.3713

#

# Coefficients:

# Estimate Std. Error z value Pr(>|z|)

# (Intercept) 1.5304 0.3700 4.136 3.54e-05 ***

# statena -3.1201 0.4167 -7.487 7.02e-14 ***

# stateNSW -2.2720 0.3762 -6.039 1.55e-09 ***

# stateQLD -2.1918 0.3824 -5.732 9.93e-09 ***

# stateSA -2.9153 0.4740 -6.150 7.75e-10 ***

# stateTAS -3.0966 0.5071 -6.107 1.02e-09 ***

# stateWA -2.6337 0.4382 -6.011 1.85e-09 ***

# vaccinationyes 3.6094 0.1775 20.331 < 2e-16 ***

# offered_byOwner -1.1599 0.1797 -6.455 1.08e-10 ***

# ---

# Signif. codes: 0 ‘***’ 0.001 ‘**’ 0.01 ‘*’ 0.05 ‘.’ 0.1 ‘ ’ 1

#

# (Dispersion parameter for binomial family taken to be 1)

#

# Null deviance: 2012.2 on 1661 degrees of freedom

# Residual deviance: 1103.1 on 1653 degrees of freedom

# AIC: 1121.1

#

# Number of Fisher Scoring iterations: 5

microchip_ra_dci8 <- confint(model_ra_microchip8)

round(exp(cbind(model_ra_microchip8$coef, microchip_ra_dci8)), digits = 2)

# 2.5 % 97.5 %

# (Intercept) 4.62 2.29 9.85

# statena 0.04 0.02 0.10

# stateNSW 0.10 0.05 0.21

# stateQLD 0.11 0.05 0.23

# stateSA 0.05 0.02 0.14

# stateTAS 0.05 0.02 0.12

# stateWA 0.07 0.03 0.17

# vaccinationyes 36.94 26.29 52.78

# offered_byOwner 0.31 0.22 0.44

lrtest(model_ra_microchip7, model_ra_microchip8)

# Likelihood ratio test

#

# Model 1: microchip ~ state + vaccination + offered_by + price_bin

# Model 2: microchip ~ state + vaccination + offered_by

# #Df LogLik Df Chisq Pr(>Chisq)

# 1 13 -539.93

# 2 9 -551.54 -4 23.219 0.0001145 ***

# ---

# Signif. codes: 0 ‘***’ 0.001 ‘**’ 0.01 ‘*’ 0.05 ‘.’ 0.1 ‘ ’ 1

# MODEL 7 IS FINAL MODEL

model_ra_microchip7_RR<-odds_to_rr(model_ra_microchip7)

format(round(model_ra_microchip7_RR,2),nsmall=2)

# RR lower.ci upper.ci

# (Intercept) 1.22 1.04 1.32

# statena 0.14 0.06 0.28

# stateNSW 0.31 0.16 0.51

# stateQLD 0.33 0.17 0.55

# stateSA 0.17 0.07 0.37

# stateTAS 0.16 0.06 0.36

# stateWA 0.22 0.10 0.42

# vaccinationyes 1.40 1.39 1.40

# offered_byOwner 0.74 0.60 0.88

# price_bin$2000+ 1.23 1.12 1.31

# price_bin$501 - $2000 1.10 0.99 1.18

# price_binFree 0.78 0.55 0.99

# price_binUnknown 1.16 0.99 1.28

#save the data set used for this as rds.

write_rds(dogads3chip,"C:/Users/Sofia Costa/OneDrive/Adelaide University Hons/Honours Program/R/dogads3chip.rds")

#####################NEW NOV REGRESSION ANALYIS - DESEXING##################

#same data as microchip. EXCEPT only odgs over 6 months of age.

#no territories.

dogads3desex <- dogads3chip

dogads3desex

#remove puppies uder 8 weeks

dogads3desex <- dogads3desex[!(dogads3desex$age_bin == "< 8 weeks old"), ]

dogads3desex

#emove puppies under 6 months

dogads3desex <- dogads3desex[!(dogads3desex$age_bin == "8 weeks - 6 months"), ]

dogads3desex

#total 499 ads to work with

agegroups <- count(dogads3desex$age_bin)

agegroups

#confirmed worked.

#save the data set used for this as rds.

write_rds(dogads3desex,"C:/Users/Sofia Costa/OneDrive/Adelaide University Hons/Honours Program/R/dogads3desex.rds")

write_csv(dogads3desex,"C:/Users/Sofia Costa/OneDrive/Adelaide University Hons/Honours Program/R/dogads3desex.csv")

######################NOV UNIVARIATE ANALYSIS - DESEXING##############

#############desexing and microchip

chi.desex.microchip_ra<- table(dogads3desex$desexed, dogads3desex$microchip)

chi.desex.microchip_ra

# 0 1

# 0 192 196

# 1 25 86

chisq.test(chi.desex.microchip_ra)

# Pearson's Chi-squared test with Yates' continuity correction

#

# data: chi.desex.microchip_ra

# X-squared = 24.445, df = 1, p-value = 7.647e-07

modelchiprad <- glm(desexed ~ microchip, family = binomial, data = dogads3desex)

summary(modelchiprad)

# Call:

# glm(formula = desexed ~ microchip, family = binomial, data = dogads3desex)

#

# Deviance Residuals:

# Min 1Q Median 3Q Max

# -0.8530 -0.8530 -0.4948 -0.4948 2.0789

#

# Coefficients:

# Estimate Std. Error z value Pr(>|z|)

# (Intercept) -2.0386 0.2126 -9.588 < 2e-16 ***

# microchip1 1.2149 0.2489 4.881 1.05e-06 ***

# ---

# Signif. codes: 0 ‘***’ 0.001 ‘**’ 0.01 ‘*’ 0.05 ‘.’ 0.1 ‘ ’ 1

#

# (Dispersion parameter for binomial family taken to be 1)

#

# Null deviance: 528.93 on 498 degrees of freedom

# Residual deviance: 501.92 on 497 degrees of freedom

# AIC: 505.92

#

# Number of Fisher Scoring iterations: 4

chip_rad_ci <- confint(modelchiprad)

round(exp(cbind(modelchiprad$coef, chip_rad_ci)), digits = 2)

# 2.5 % 97.5 %

# (Intercept) 0.13 0.08 0.19

# microchip1 3.37 2.10 5.58

modelchip_rad_RR<-odds_to_rr(modelchiprad)

format(round(modelchip_rad_RR,2),nsmall=2)

# RR lower.ci upper.ci

# (Intercept) 0.16 0.11 0.24

# microchip1 2.21 1.69 2.76

###########desexing and pure/cross

chi.desex.pure_rad<- table(dogads3desex$desexed, dogads3desex$pure_cross)

chi.desex.pure_rad

# cross designer pure unknown

# 0 106 7 267 8

# 1 38 4 64 5

chisq.test(chi.desex.pure_rad)

# Pearson's Chi-squared test

#

# data: chi.desex.pure_rad

# X-squared = 6.2941, df = 3, p-value = 0.09815

#

# Warning message:

# In chisq.test(chi.desex.pure_rad) :

# Chi-squared approximation may be incorrect

#using fishers exact test because coutns too small

fisher.test(chi.desex.pure_rad)

modelpure_rad <- glm(desexed ~ pure_cross, family = binomial, data = dogads3desex)

summary(modelpure_rad)

# Call:

# glm(formula = desexed ~ pure_cross, family = binomial, data = dogads3desex)

#

# Deviance Residuals:

# Min 1Q Median 3Q Max

# -0.9854 -0.6555 -0.6555 -0.6555 1.8129

#

# Coefficients:

# Estimate Std. Error z value Pr(>|z|)

# (Intercept) -1.0259 0.1891 -5.426 5.78e-08 ***

# pure_crossdesigner 0.4662 0.6547 0.712 0.4764

# pure_crosspure -0.4025 0.2348 -1.714 0.0864 .

# pure_crossunknown 0.5558 0.6006 0.925 0.3547

# ---

# Signif. codes: 0 ‘***’ 0.001 ‘**’ 0.01 ‘*’ 0.05 ‘.’ 0.1 ‘ ’ 1

#

# (Dispersion parameter for binomial family taken to be 1)

#

# Null deviance: 528.93 on 498 degrees of freedom

# Residual deviance: 523.02 on 495 degrees of freedom

# AIC: 531.02

#

# Number of Fisher Scoring iterations: 4

pure_rad_ci <- confint(modelpure_rad)

round(exp(cbind(modelpure_rad$coef, pure_rad_ci)), digits = 2)

# 2.5 % 97.5 %

# (Intercept) 0.36 0.24 0.51

# pure_crossdesigner 1.59 0.40 5.59

# pure_crosspure 0.67 0.42 1.07

# pure_crossunknown 1.74 0.50 5.56

modelpure_rad_RR<-odds_to_rr(modelpure_rad)

format(round(modelpure_rad_RR,2),nsmall=2)

# RR lower.ci upper.ci

# (Intercept) 0.42 0.29 0.58

# pure_crossdesigner 1.41 0.46 2.77

# pure_crosspure 0.72 0.49 1.05

# pure_crossunknown 1.50 0.56 2.76

################desexing and state

chistate_rad<- table(dogads3desex$desexed,dogads3desex$state)

chistate_rad

# VIC na NSW QLD SA TAS WA

# 0 47 51 121 103 17 16 33

# 1 15 8 44 24 6 6 8

# >

chisq.test(chistate_rad)

# Pearson's Chi-squared test

#

# data: chistate_rad

# X-squared = 6.0922, df = 6, p-value = 0.4129

#

# Warning message:

# In chisq.test(chistate_rad) : Chi-squared approximation may be incorrect

#sNOT ENOUGH COUNT - AMALGAMATE STATES INTO 'DOES REQUIRE DESEX' AND 'DOES NOT REQUIRE DESEX'

#make new column called 'requiring desexing'

#create bins

dogads3desex <-

dogads3desex %>%

mutate(

ID_desex_required = case_when(

state == "SA" ~ "required",

state == "NSW" ~ "not required",

state == "QLD" ~ "not required",

state == "VIC" ~ "not required",

state == "WA" ~ "not required",

state == "TAS" ~ "not required",

is.na(state) ~ "unclear",

)

)

table(dogads3desex$ID_desex_required)

##fix states requirign desex to include na's as - unclear

dogads3desex$ID_desex_required [is.na (dogads3desex$ID_desex_required)] <- "unclear"

table(dogads3desex$ID_desex_required)

chistate_rad2<- table(dogads3desex$desexed,dogads3desex$ID_desex_required)

chistate_rad2

# not required required unclear

# 0 320 17 51

# 1 97 6 8

chisq.test(chistate_rad2)

# Pearson's Chi-squared test

#

# data: chistate_rad2

# X-squared = 3.0187, df = 2, p-value = 0.2211

modelchi_state <- glm(desexed ~ ID_desex_required, family = binomial, data = dogads3desex)

summary(modelchi_state)

# Call:

# glm(formula = desexed ~ ID_desex_required, family = binomial,

# data = dogads3desex)

#

# Deviance Residuals:

# Min 1Q Median 3Q Max

# -0.7775 -0.7277 -0.7277 -0.5398 1.9990

#

# Coefficients:

# Estimate Std. Error z value Pr(>|z|)

# (Intercept) -1.1936 0.1159 -10.298 <2e-16 ***

# ID_desex_requiredrequired 0.1522 0.4888 0.311 0.7556

# ID_desex_requiredunclear -0.6588 0.3975 -1.657 0.0975 .

# ---

# Signif. codes: 0 ‘***’ 0.001 ‘**’ 0.01 ‘*’ 0.05 ‘.’ 0.1 ‘ ’ 1

#

# (Dispersion parameter for binomial family taken to be 1)

#

# Null deviance: 528.93 on 498 degrees of freedom

# Residual deviance: 525.61 on 496 degrees of freedom

# AIC: 531.61

#

# Number of Fisher Scoring iterations: 4

state_ra_ci <- confint(modelchi_state)

round(exp(cbind(modelchi_state$coef, state_ra_ci)), digits = 2)

# 2.5 % 97.5 %

# (Intercept) 0.30 0.24 0.38

# ID_desex_requiredrequired 1.16 0.41 2.89

# ID_desex_requiredunclear 0.52 0.22 1.07

modelrequire_ra_RR<-odds_to_rr(modelchi_state)

format(round(modelrequire_ra_RR,2),nsmall=2)

# RR lower.ci upper.ci

# (Intercept) 0.36 0.29 0.44

# ID_desex_requiredrequired 1.12 0.47 2.03

# ID_desex_requiredunclear 0.58 0.27 1.05

##########desexing and vx

chi.vx_rad <- table(dogads3desex$desexed, dogads3desex$vaccination)

chi.vx_rad

# no yes

# 0 179 209

# 1 33 78

chisq.test(chi.vx_rad)

# Pearson's Chi-squared test with Yates' continuity correction

#

# data: chi.vx_rad

# X-squared = 8.8455, df = 1, p-value = 0.002938

modelvx_rad <- glm(desexed ~ vaccination, family = binomial, data = dogads3desex)

summary(modelvx_rad)

# Call:

# glm(formula = desexed ~ vaccination, family = binomial, data = dogads3desex)

#

# Deviance Residuals:

# Min 1Q Median 3Q Max

# -0.7964 -0.7964 -0.5817 -0.5817 1.9288

#

# Coefficients:

# Estimate Std. Error z value Pr(>|z|)

# (Intercept) -1.6909 0.1894 -8.925 < 2e-16 ***

# vaccinationyes 0.7053 0.2313 3.049 0.00229 **

# ---

# Signif. codes: 0 ‘***’ 0.001 ‘**’ 0.01 ‘*’ 0.05 ‘.’ 0.1 ‘ ’ 1

#

# (Dispersion parameter for binomial family taken to be 1)

#

# Null deviance: 528.93 on 498 degrees of freedom

# Residual deviance: 519.14 on 497 degrees of freedom

# AIC: 523.14

#

# Number of Fisher Scoring iterations: 4

vx_rad_ci <- confint(modelvx_rad)

round(exp(cbind(modelvx_rad$coef, vx_rad_ci)), digits = 2)

# 2.5 % 97.5 %

# (Intercept) 0.18 0.13 0.26

# vaccinationyes 2.02 1.30 3.22

modelvx_rad_RR<-odds_to_rr(modelvx_rad)

format(round(modelvx_rad_RR,2),nsmall=2)

# RR lower.ci upper.ci

# (Intercept) 0.23 0.16 0.31

# vaccinationyes 1.65 1.22 2.15

########desexing and org registered

chi.org_rad <- table(dogads3desex$desexed, dogads3desex$breeder_org_affiliation)

chi.org_rad

# none yes

# 0 343 45

# 1 106 5

chisq.test(chi.org_rad)

# Pearson's Chi-squared test with Yates' continuity correction

#

# data: chi.org_rad

# X-squared = 4.0621, df = 1, p-value = 0.04386

modelorg_rad <- glm(desexed ~ breeder_org_affiliation, family = binomial, data = dogads3desex)

summary(modelorg_rad)

# Call:

# glm(formula = desexed ~ breeder_org_affiliation, family = binomial,

# data = dogads3desex)

#

# Deviance Residuals:

# Min 1Q Median 3Q Max

# -0.7339 -0.7339 -0.7339 -0.4590 2.1460

#

# Coefficients:

# Estimate Std. Error z value Pr(>|z|)

# (Intercept) -1.1743 0.1111 -10.567 <2e-16 ***

# breeder_org_affiliationyes -1.0229 0.4843 -2.112 0.0347 *

# ---

# Signif. codes: 0 ‘***’ 0.001 ‘**’ 0.01 ‘*’ 0.05 ‘.’ 0.1 ‘ ’ 1

#

# (Dispersion parameter for binomial family taken to be 1)

#

# Null deviance: 528.93 on 498 degrees of freedom

# Residual deviance: 523.28 on 497 degrees of freedom

# AIC: 527.28

#

# Number of Fisher Scoring iterations: 4

org_rad_ci <- confint(modelorg_rad)

round(exp(cbind(modelorg_rad$coef, org_rad_ci)), digits = 2)

# 2.5 % 97.5 %

# (Intercept) 0.31 0.25 0.38

# breeder_org_affiliationyes 0.36 0.12 0.85

modelorg_rad_RR<-odds_to_rr(modelorg_rad)

format(round(modelorg_rad_RR,2),nsmall=2)

# RR lower.ci upper.ci

# (Intercept) 0.37 0.30 0.44

# breeder_org_affiliationyes 0.42 0.15 0.88

#######desexing and price bin

chi.price_rad<- table(dogads3desex$desexed, dogads3desex$price_bin)

chi.price_rad

# $1 - $500 $2000+ $501 - $2000 Free Unknown

# 0 137 47 118 58 28

# 1 52 6 25 24 4

chisq.test(chi.price_rad)

# Pearson's Chi-squared test

#

# data: chi.price_rad

# X-squared = 12.66, df = 4, p-value = 0.01306

modelprice_rad <- glm(desexed ~ price_bin, family = binomial, data = dogads3desex)

summary(modelprice_rad)

# Call:

# glm(formula = desexed ~ price_bin, family = binomial, data = dogads3desex)

#

# Deviance Residuals:

# Min 1Q Median 3Q Max

# -0.8322 -0.8022 -0.6199 -0.4902 2.0874

#

# Coefficients:

# Estimate Std. Error z value Pr(>|z|)

# (Intercept) -0.96874 0.16288 -5.948 2.72e-09 ***

# price_bin$2000+ -1.08965 0.46310 -2.353 0.0186 *

# price_bin$501 - $2000 -0.58307 0.27387 -2.129 0.0333 *

# price_binFree 0.08635 0.29230 0.295 0.7677

# price_binUnknown -0.97717 0.55879 -1.749 0.0803 .

# ---

# Signif. codes: 0 ‘***’ 0.001 ‘**’ 0.01 ‘*’ 0.05 ‘.’ 0.1 ‘ ’ 1

#

# (Dispersion parameter for binomial family taken to be 1)

#

# Null deviance: 528.93 on 498 degrees of freedom

# Residual deviance: 515.62 on 494 degrees of freedom

# AIC: 525.62

#

# Number of Fisher Scoring iterations: 4

price_rad_ci <- confint(modelprice_rad)

round(exp(cbind(modelprice_rad$coef, price_rad_ci)), digits = 2)

# 2.5 % 97.5 %

# (Intercept) 0.38 0.27 0.52

# price_bin$2000+ 0.34 0.12 0.78

# price_bin$501 - $2000 0.56 0.32 0.95

# price_binFree 1.09 0.61 1.92

# price_binUnknown 0.38 0.11 1.02

modelprice_rad_RR<-odds_to_rr(modelprice_rad)

format(round(modelprice_rad_RR,2),nsmall=2)

# RR lower.ci upper.ci

# (Intercept) 0.44 0.33 0.58

# price_bin$2000+ 0.39 0.15 0.82

# price_bin$501 - $2000 0.62 0.38 0.96

# price_binFree 1.07 0.67 1.59

# price_binUnknown 0.44 0.13 1.01

####desexing and age bin

chi.age_rad <- table(dogads3desex$desexed, dogads3desex$age_bin)

chi.age_rad

#ages prior to 6 months old are coming up as factors with 0, need to remove factors

#compeltley.

dogads3desex <- dogads3desex[ dogads3desex$age_bin != "< 8 weeks old", , drop=FALSE]; dogads3desex$age_bin <- factor(dogads3desex$age_bin); summary(dogads3desex)

dogads3desex

table(dogadsra$offered_by)

#s< 8 weeks old has been removed.

chi.age_rad <- table(dogads3desex$desexed, dogads3desex$age_bin)

chi.age_rad

# 1 - 3 years 3 + years 6 months - 1 year Unknown

# 0 146 66 129 47

# 1 48 38 18 7

chisq.test(chi.age_rad)

# Pearson's Chi-squared test

#

# data: chi.age_rad

# X-squared = 24.173, df = 3, p-value = 2.299e-05

modelage_rad <- glm(desexed ~ age_bin, family = binomial, data = dogads3desex)

summary(modelage_rad)

# Call:

# glm(formula = desexed ~ age_bin, family = binomial, data = dogads3desex)

#

# Deviance Residuals:

# Min 1Q Median 3Q Max

# -0.9537 -0.7540 -0.5270 -0.5111 2.0494

#

# Coefficients:

# Estimate Std. Error z value Pr(>|z|)

# (Intercept) -1.1124 0.1664 -6.686 2.3e-11 ***

# age_bin3 + years 0.5603 0.2630 2.131 0.03310 *

# age_bin6 months - 1 year -0.8570 0.3016 -2.841 0.00449 **

# age_binUnknown -0.7918 0.4380 -1.808 0.07061 .

# ---

# Signif. codes: 0 ‘***’ 0.001 ‘**’ 0.01 ‘*’ 0.05 ‘.’ 0.1 ‘ ’ 1

#

# (Dispersion parameter for binomial family taken to be 1)

#

# Null deviance: 528.93 on 498 degrees of freedom

# Residual deviance: 504.58 on 495 degrees of freedom

# AIC: 512.58

#

# Number of Fisher Scoring iterations: 4

age_radci <- confint(modelage_rad)

round(exp(cbind(modelage_rad$coef, age_radci)), digits = 2)

# 2.5 % 97.5 %

# (Intercept) 0.33 0.23 0.45

# age_bin3 + years 1.75 1.04 2.93

# age_bin6 months - 1 year 0.42 0.23 0.75

# age_binUnknown 0.45 0.18 1.01

modelage_rad_RR<-odds_to_rr(modelage_rad)

format(round(modelage_rad_RR,2),nsmall=2)

# RR lower.ci upper.ci

# (Intercept) 0.39 0.28 0.51

# age_bin3 + years 1.50 1.03 2.05

# age_bin6 months - 1 year 0.49 0.28 0.80

# age_binUnknown 0.52 0.22 1.01

>

######desexing and offered by

chi_offered_rad<- table(dogads3desex$desexed, dogads3desex$offered_by)

chi_offered_rad

# Breeder Owner

# 0 70 318

# 1 9 102

chisq.test(chi_offered_rad)

#

# Pearson's Chi-squared test with Yates' continuity correction

#

# data: chi_offered_rad

# X-squared = 5.667, df = 1, p-value = 0.01729

modeloffered_rad <- glm(desexed ~ offered_by, family = binomial, data = dogads3desex)

summary(modeloffered_rad)

# Call:

# glm(formula = desexed ~ offered_by, family = binomial, data = dogads3desex)

#

# Deviance Residuals:

# Min 1Q Median 3Q Max

# -0.7459 -0.7459 -0.7459 -0.4918 2.0843

#

# Coefficients:

# Estimate Std. Error z value Pr(>|z|)

# (Intercept) -2.0513 0.3541 -5.793 6.92e-09 ***

# offered_byOwner 0.9142 0.3719 2.458 0.014 *

# ---

# Signif. codes: 0 ‘***’ 0.001 ‘**’ 0.01 ‘*’ 0.05 ‘.’ 0.1 ‘ ’ 1

#

# (Dispersion parameter for binomial family taken to be 1)

#

# Null deviance: 528.93 on 498 degrees of freedom

# Residual deviance: 521.69 on 497 degrees of freedom

# AIC: 525.69

#

# Number of Fisher Scoring iterations: 4

offered_radci <- confint(modeloffered_rad)

round(exp(cbind(modeloffered_rad$coef, offered_radci)), digits = 2)

# 2.5 % 97.5 %

# (Intercept) 0.13 0.06 0.24

# offered_byOwner 2.49 1.26 5.52

modeloffered_rad_RR<-odds_to_rr(modeloffered_rad)

format(round(modeloffered_rad_RR,2),nsmall=2)

# RR lower.ci upper.ci

# (Intercept) 0.16 0.08 0.29

# offered_byOwner 1.87 1.19 2.75

##############NEW NOV REGRESSION MODEL - DESEXING###################

#everything with a sig of 0.25 and below is put into model. Id desex required (state regulated) not sig.

#WITH ALL VARIABLES - inc. if

model_ra_desex <- glm(desexed ~ microchip + vaccination +

pure_cross + offered_by + breeder_org_affiliation +

age_bin + price_bin, family = binomial,

data = dogads3desex)

summary(model_ra_desex)

# Call:

# glm(formula = desexed ~ microchip + vaccination + pure_cross +

# offered_by + breeder_org_affiliation + age_bin + price_bin,

# family = binomial, data = dogads3desex)

#

# Deviance Residuals:

# Min 1Q Median 3Q Max

# -1.5119 -0.6712 -0.4488 -0.2189 2.6557

#

# Coefficients:

# Estimate Std. Error z value Pr(>|z|)

# (Intercept) -2.6484 0.6085 -4.352 1.35e-05 ***

# microchip1 1.5527 0.3380 4.593 4.37e-06 ***

# vaccinationyes 0.2577 0.3202 0.805 0.42096

# pure_crossdesigner 1.5557 0.7407 2.100 0.03571 *

# pure_crosspure -0.3356 0.2793 -1.202 0.22946

# pure_crossunknown 0.6365 0.6751 0.943 0.34579

# offered_byOwner 0.8657 0.4896 1.768 0.07703 .

# breeder_org_affiliationyes -0.3285 0.6346 -0.518 0.60468

# age_bin3 + years 0.6265 0.2999 2.089 0.03674 *

# age_bin6 months - 1 year -1.0646 0.3337 -3.190 0.00142 **

# age_binUnknown -0.8653 0.4678 -1.850 0.06432 .

# price_bin$2000+ -0.9296 0.5501 -1.690 0.09101 .

# price_bin$501 - $2000 -0.2059 0.3244 -0.635 0.52555

# price_binFree 0.1049 0.3419 0.307 0.75904

# price_binUnknown -1.0498 0.5974 -1.757 0.07889 .

# ---

# Signif. codes: 0 ‘***’ 0.001 ‘**’ 0.01 ‘*’ 0.05 ‘.’ 0.1 ‘ ’ 1

#

# (Dispersion parameter for binomial family taken to be 1)

#

# Null deviance: 528.93 on 498 degrees of freedom

# Residual deviance: 441.77 on 484 degrees of freedom

# AIC: 471.77

#

# Number of Fisher Scoring iterations: 5

desexraci <- confint(model_ra_desex)

round(exp(cbind(model_ra_desex$coef, desexraci)), digits = 2)

#

# 2.5 % 97.5 %

# (Intercept) 0.07 0.02 0.22

# microchip1 4.72 2.47 9.33

# vaccinationyes 1.29 0.69 2.43

# pure_crossdesigner 4.74 1.04 20.03

# pure_crosspure 0.71 0.41 1.24

# pure_crossunknown 1.89 0.48 7.07

# offered_byOwner 2.38 0.95 6.58

# breeder_org_affiliationyes 0.72 0.19 2.41

# age_bin3 + years 1.87 1.04 3.38

# age_bin6 months - 1 year 0.34 0.18 0.65

# age_binUnknown 0.42 0.16 1.00

# price_bin$2000+ 0.39 0.12 1.10

# price_bin$501 - $2000 0.81 0.43 1.53

# price_binFree 1.11 0.56 2.16

# price_binUnknown 0.35 0.09 1.03

# model2 - model1 minus microchip

model_ra_desex2 <- glm(desexed ~ vaccination +

pure_cross + offered_by + breeder_org_affiliation +

age_bin + price_bin, family = binomial,

data = dogads3desex)

summary(model_ra_desex2)

# Call:

# glm(formula = desexed ~ vaccination + pure_cross + offered_by +

# breeder_org_affiliation + age_bin + price_bin, family = binomial,

# data = dogads3desex)

#

# Deviance Residuals:

# Min 1Q Median 3Q Max

# -1.3929 -0.7178 -0.5151 -0.2825 2.6073

#

# Coefficients:

# Estimate Std. Error z value Pr(>|z|)

# (Intercept) -2.1752 0.5839 -3.725 0.000195 ***

# vaccinationyes 1.1530 0.2556 4.512 6.43e-06 ***

# pure_crossdesigner 1.2459 0.7505 1.660 0.096892 .

# pure_crosspure -0.2635 0.2676 -0.985 0.324783

# pure_crossunknown 0.6093 0.6638 0.918 0.358651

# offered_byOwner 0.7773 0.4816 1.614 0.106565

# breeder_org_affiliationyes -0.3345 0.6320 -0.529 0.596579

# age_bin3 + years 0.7384 0.2901 2.545 0.010930 *

# age_bin6 months - 1 year -0.9794 0.3228 -3.034 0.002416 **

# age_binUnknown -0.6886 0.4581 -1.503 0.132817

# price_bin$2000+ -0.7655 0.5466 -1.401 0.161351

# price_bin$501 - $2000 -0.2916 0.3142 -0.928 0.353407

# price_binFree -0.1139 0.3263 -0.349 0.727007

# price_binUnknown -0.9863 0.5849 -1.686 0.091740 .

# ---

# Signif. codes: 0 ‘***’ 0.001 ‘**’ 0.01 ‘*’ 0.05 ‘.’ 0.1 ‘ ’ 1

#

# (Dispersion parameter for binomial family taken to be 1)

#

# Null deviance: 528.93 on 498 degrees of freedom

# Residual deviance: 464.96 on 485 degrees of freedom

# AIC: 492.96

#

# Number of Fisher Scoring iterations: 5

desexraci2 <- confint(model_ra_desex2)

round(exp(cbind(model_ra_desex2$coef, desexraci2)), digits = 2)

# 2.5 % 97.5 %

# (Intercept) 0.11 0.03 0.34

# vaccinationyes 3.17 1.94 5.29

# pure_crossdesigner 3.48 0.75 14.95

# pure_crosspure 0.77 0.46 1.30

# pure_crossunknown 1.84 0.48 6.70

# offered_byOwner 2.18 0.89 5.94

# breeder_org_affiliationyes 0.72 0.19 2.38

# age_bin3 + years 2.09 1.19 3.71

# age_bin6 months - 1 year 0.38 0.19 0.70

# age_binUnknown 0.50 0.19 1.17

# price_bin$2000+ 0.47 0.15 1.29

# price_bin$501 - $2000 0.75 0.40 1.37

# price_binFree 0.89 0.47 1.68

# price_binUnknown 0.37 0.10 1.07

lrtest(model_ra_desex, model_ra_desex2)

# Likelihood ratio test

#

# Model 1: desexed ~ microchip + vaccination + pure_cross + offered_by +

# breeder_org_affiliation + age_bin + price_bin

# Model 2: desexed ~ vaccination + pure_cross + offered_by + breeder_org_affiliation +

# age_bin + price_bin

# #Df LogLik Df Chisq Pr(>Chisq)

# 1 15 -220.88

# 2 14 -232.48 -1 23.186 1.47e-06 ***

# ---

# Signif. codes: 0 ‘***’ 0.001 ‘**’ 0.01 ‘*’ 0.05 ‘.’ 0.1 ‘ ’ 1

#microchipping is significant.

#model 3: model1 minus vaccination

model_ra_desex3 <- glm(desexed ~ microchip +

pure_cross + offered_by + breeder_org_affiliation +

age_bin + price_bin, family = binomial,

data = dogads3desex)

summary(model_ra_desex3)

# Call:

# glm(formula = desexed ~ microchip + pure_cross + offered_by +

# breeder_org_affiliation + age_bin + price_bin, family = binomial,

# data = dogads3desex)

#

# Deviance Residuals:

# Min 1Q Median 3Q Max

# -1.4816 -0.6699 -0.4468 -0.2099 2.6678

#

# Coefficients:

# Estimate Std. Error z value Pr(>|z|)

# (Intercept) -2.5676 0.5992 -4.285 1.83e-05 ***

# microchip1 1.7100 0.2782 6.146 7.97e-10 ***

# pure_crossdesigner 1.6012 0.7359 2.176 0.02956 *

# pure_crosspure -0.3364 0.2795 -1.204 0.22865

# pure_crossunknown 0.6308 0.6745 0.935 0.34963

# offered_byOwner 0.8474 0.4887 1.734 0.08288 .

# breeder_org_affiliationyes -0.3216 0.6331 -0.508 0.61148

# age_bin3 + years 0.5886 0.2957 1.991 0.04650 *

# age_bin6 months - 1 year -1.0680 0.3340 -3.198 0.00138 **

# age_binUnknown -0.8519 0.4665 -1.826 0.06784 .

# price_bin$2000+ -0.9460 0.5487 -1.724 0.08472 .

# price_bin$501 - $2000 -0.1975 0.3239 -0.610 0.54198

# price_binFree 0.1132 0.3410 0.332 0.73990

# price_binUnknown -1.0649 0.5975 -1.782 0.07468 .

# ---

# Signif. codes: 0 ‘***’ 0.001 ‘**’ 0.01 ‘*’ 0.05 ‘.’ 0.1 ‘ ’ 1

#

# (Dispersion parameter for binomial family taken to be 1)

#

# Null deviance: 528.93 on 498 degrees of freedom

# Residual deviance: 442.42 on 485 degrees of freedom

# AIC: 470.42

#

# Number of Fisher Scoring iterations: 5

desexraci3 <- confint(model_ra_desex3)

round(exp(cbind(model_ra_desex3$coef, desexraci3)), digits = 2)

# 2.5 % 97.5 %

# (Intercept) 0.08 0.02 0.24

# microchip1 5.53 3.26 9.73

# pure_crossdesigner 4.96 1.09 20.75

# pure_crosspure 0.71 0.41 1.24

# pure_crossunknown 1.88 0.48 7.02

# offered_byOwner 2.33 0.93 6.44

# breeder_org_affiliationyes 0.72 0.19 2.42

# age_bin3 + years 1.80 1.01 3.22

# age_bin6 months - 1 year 0.34 0.17 0.65

# age_binUnknown 0.43 0.16 1.02

# price_bin$2000+ 0.39 0.12 1.08

# price_bin$501 - $2000 0.82 0.43 1.54

# price_binFree 1.12 0.57 2.18

# price_binUnknown 0.34 0.09 1.01

lrtest(model_ra_desex, model_ra_desex3)

# Likelihood ratio test

#

# Model 1: desexed ~ microchip + vaccination + pure_cross + offered_by +

# breeder_org_affiliation + age_bin + price_bin

# Model 2: desexed ~ microchip + pure_cross + offered_by + breeder_org_affiliation +

# age_bin + price_bin

# #Df LogLik Df Chisq Pr(>Chisq)

# 1 15 -220.88

# 2 14 -221.21 -1 0.6484 0.4207

#vaccinatin is not sig.

#model 4: model3 minus pure_cross

model_ra_desex4 <- glm(desexed ~ microchip +

offered_by + breeder_org_affiliation +

age_bin + price_bin, family = binomial,

data = dogads3desex)

summary(model_ra_desex4)

# Call:

# glm(formula = desexed ~ microchip + offered_by + breeder_org_affiliation +

# age_bin + price_bin, family = binomial, data = dogads3desex)

#

# Deviance Residuals:

# Min 1Q Median 3Q Max

# -1.4014 -0.7098 -0.4833 -0.2547 2.5588

#

# Coefficients:

# Estimate Std. Error z value Pr(>|z|)

# (Intercept) -2.5716 0.5518 -4.661 3.15e-06 ***

# microchip1 1.6114 0.2704 5.960 2.52e-09 ***

# offered_byOwner 0.7526 0.4768 1.579 0.11444

# breeder_org_affiliationyes -0.4241 0.6229 -0.681 0.49597

# age_bin3 + years 0.5691 0.2926 1.945 0.05180 .

# age_bin6 months - 1 year -0.9424 0.3191 -2.953 0.00314 **

# age_binUnknown -0.8701 0.4630 -1.879 0.06020 .

# price_bin$2000+ -0.9084 0.5278 -1.721 0.08520 .

# price_bin$501 - $2000 -0.2692 0.3085 -0.873 0.38281

# price_binFree 0.1513 0.3353 0.451 0.65179

# price_binUnknown -1.0318 0.5901 -1.748 0.08038 .

# ---

# Signif. codes: 0 ‘***’ 0.001 ‘**’ 0.01 ‘*’ 0.05 ‘.’ 0.1 ‘ ’ 1

#

# (Dispersion parameter for binomial family taken to be 1)

#

# Null deviance: 528.93 on 498 degrees of freedom

# Residual deviance: 451.29 on 488 degrees of freedom

# AIC: 473.29

#

# Number of Fisher Scoring iterations: 5

desexraci4 <- confint(model_ra_desex4)

round(exp(cbind(model_ra_desex4$coef, desexraci4)), digits = 2)

# 2.5 % 97.5 %

# (Intercept) 0.08 0.02 0.22

# microchip1 5.01 2.99 8.67

# offered_byOwner 2.12 0.87 5.72

# breeder_org_affiliationyes 0.65 0.18 2.14

# age_bin3 + years 1.77 0.99 3.14

# age_bin6 months - 1 year 0.39 0.20 0.72

# age_binUnknown 0.42 0.16 0.99

# price_bin$2000+ 0.40 0.13 1.08

# price_bin$501 - $2000 0.76 0.41 1.39

# price_binFree 1.16 0.60 2.24

# price_binUnknown 0.36 0.10 1.03

lrtest(model_ra_desex4, model_ra_desex3)

# Likelihood ratio test

#

# Model 1: desexed ~ microchip + offered_by + breeder_org_affiliation +

# age_bin + price_bin

# Model 2: desexed ~ microchip + pure_cross + offered_by + breeder_org_affiliation +

# age_bin + price_bin

# #Df LogLik Df Chisq Pr(>Chisq)

# 1 11 -225.65

# 2 14 -221.21 3 8.8773 0.03097 *

# ---

# Signif. codes: 0 ‘***’ 0.001 ‘**’ 0.01 ‘*’ 0.05 ‘.’ 0.1 ‘ ’ 1

#pure cross is significant

#model 5 : model 3 minus offered by

model_ra_desex5 <- glm(desexed ~ microchip +

pure_cross + breeder_org_affiliation +

age_bin + price_bin, family = binomial,

data = dogads3desex)

summary(model_ra_desex5)

# Call:

# glm(formula = desexed ~ microchip + pure_cross + breeder_org_affiliation +

# age_bin + price_bin, family = binomial, data = dogads3desex)

#

# Deviance Residuals:

# Min 1Q Median 3Q Max

# -1.4767 -0.6699 -0.4431 -0.2422 2.6254

#

# Coefficients:

# Estimate Std. Error z value Pr(>|z|)

# (Intercept) -1.7060 0.3316 -5.145 2.67e-07 ***

# microchip1 1.6685 0.2770 6.024 1.70e-09 ***

# pure_crossdesigner 1.4477 0.7303 1.982 0.04744 *

# pure_crosspure -0.3654 0.2787 -1.311 0.18989

# pure_crossunknown 0.5506 0.6651 0.828 0.40778

# breeder_org_affiliationyes -0.8484 0.5548 -1.529 0.12621

# age_bin3 + years 0.5895 0.2945 2.002 0.04531 *

# age_bin6 months - 1 year -1.0266 0.3315 -3.097 0.00196 **

# age_binUnknown -0.8940 0.4633 -1.930 0.05367 .

# price_bin$2000+ -1.0204 0.5436 -1.877 0.06050 .

# price_bin$501 - $2000 -0.3160 0.3183 -0.993 0.32090

# price_binFree 0.1287 0.3390 0.380 0.70427

# price_binUnknown -1.0557 0.5951 -1.774 0.07608 .

# ---

# Signif. codes: 0 ‘***’ 0.001 ‘**’ 0.01 ‘*’ 0.05 ‘.’ 0.1 ‘ ’ 1

#

# (Dispersion parameter for binomial family taken to be 1)

#

# Null deviance: 528.93 on 498 degrees of freedom

# Residual deviance: 445.68 on 486 degrees of freedom

# AIC: 471.68

#

# Number of Fisher Scoring iterations: 5

desexraci5 <- confint(model_ra_desex5)

round(exp(cbind(model_ra_desex5$coef, desexraci5)), digits = 2)

# 2.5 % 97.5 %

# (Intercept) 0.18 0.09 0.34

# microchip1 5.30 3.13 9.31

# pure_crossdesigner 4.25 0.95 17.60

# pure_crosspure 0.69 0.40 1.20

# pure_crossunknown 1.73 0.45 6.35

# breeder_org_affiliationyes 0.43 0.13 1.19

# age_bin3 + years 1.80 1.01 3.22

# age_bin6 months - 1 year 0.36 0.18 0.67

# age_binUnknown 0.41 0.15 0.97

# price_bin$2000+ 0.36 0.11 0.99

# price_bin$501 - $2000 0.73 0.39 1.35

# price_binFree 1.14 0.58 2.20

# price_binUnknown 0.35 0.09 1.02

lrtest(model_ra_desex5, model_ra_desex3)

# Likelihood ratio test

#

# Model 1: desexed ~ microchip + pure_cross + breeder_org_affiliation +

# age_bin + price_bin

# Model 2: desexed ~ microchip + pure_cross + offered_by + breeder_org_affiliation +

# age_bin + price_bin

# #Df LogLik Df Chisq Pr(>Chisq)

# 1 13 -222.84

# 2 14 -221.21 1 3.2667 0.0707 .

# ---

# Signif. codes: 0 ‘***’ 0.001 ‘**’ 0.01 ‘*’ 0.05 ‘.’ 0.1 ‘ ’ 1

#offered by not sig

#model 6 : model 5 minus breeder org affiliation

model_ra_desex6 <- glm(desexed ~ microchip +

pure_cross +

age_bin + price_bin, family = binomial,

data = dogads3desex)

summary(model_ra_desex6)

# Call:

# glm(formula = desexed ~ microchip + pure_cross + age_bin + price_bin,

# family = binomial, data = dogads3desex)

#

# Deviance Residuals:

# Min 1Q Median 3Q Max

# -1.4741 -0.6763 -0.4878 -0.2429 2.6599

#

# Coefficients:

# Estimate Std. Error z value Pr(>|z|)

# (Intercept) -1.6805 0.3309 -5.078 3.8e-07 ***

# microchip1 1.6453 0.2766 5.949 2.7e-09 ***

# pure_crossdesigner 1.4466 0.7258 1.993 0.04623 *

# pure_crosspure -0.3884 0.2790 -1.392 0.16385

# pure_crossunknown 0.5319 0.6621 0.803 0.42173

# age_bin3 + years 0.5762 0.2930 1.967 0.04922 *

# age_bin6 months - 1 year -1.0035 0.3303 -3.038 0.00238 **

# age_binUnknown -0.9359 0.4616 -2.027 0.04263 *

# price_bin$2000+ -1.3749 0.5054 -2.720 0.00652 **

# price_bin$501 - $2000 -0.4358 0.3116 -1.399 0.16195

# price_binFree 0.1338 0.3382 0.396 0.69234

# price_binUnknown -1.0452 0.5946 -1.758 0.07879 .

# ---

# Signif. codes: 0 ‘***’ 0.001 ‘**’ 0.01 ‘*’ 0.05 ‘.’ 0.1 ‘ ’ 1

#

# (Dispersion parameter for binomial family taken to be 1)

#

# Null deviance: 528.93 on 498 degrees of freedom

# Residual deviance: 448.28 on 487 degrees of freedom

# AIC: 472.28

#

# Number of Fisher Scoring iterations: 5

desexraci6 <- confint(model_ra_desex6)

round(exp(cbind(model_ra_desex6$coef, desexraci6)), digits = 2)

# 2.5 % 97.5 %

# (Intercept) 0.19 0.10 0.35

# microchip1 5.18 3.06 9.09

# pure_crossdesigner 4.25 0.95 17.40

# pure_crosspure 0.68 0.39 1.17

# pure_crossunknown 1.70 0.44 6.19

# age_bin3 + years 1.78 1.00 3.17

# age_bin6 months - 1 year 0.37 0.19 0.69

# age_binUnknown 0.39 0.15 0.92

# price_bin$2000+ 0.25 0.09 0.64

# price_bin$501 - $2000 0.65 0.35 1.18

# price_binFree 1.14 0.58 2.21

# price_binUnknown 0.35 0.09 1.03

lrtest(model_ra_desex5, model_ra_desex6)

# Likelihood ratio test

#

# Model 1: desexed ~ microchip + pure_cross + breeder_org_affiliation +

# age_bin + price_bin

# Model 2: desexed ~ microchip + pure_cross + age_bin + price_bin

# #Df LogLik Df Chisq Pr(>Chisq)

# 1 13 -222.84

# 2 12 -224.14 -1 2.5939 0.1073

#breeder org not sig.

#model 7 : model 6 minus age

model_ra_desex7 <- glm(desexed ~ microchip +

pure_cross +

price_bin, family = binomial,

data = dogads3desex)

summary(model_ra_desex7)

# Call:

# glm(formula = desexed ~ microchip + pure_cross + price_bin, family = binomial,

# data = dogads3desex)

#

# Deviance Residuals:

# Min 1Q Median 3Q Max

# -1.3710 -0.7199 -0.4904 -0.2898 2.3666

#

# Coefficients:

# Estimate Std. Error z value Pr(>|z|)

# (Intercept) -1.8265 0.2988 -6.112 9.83e-10 ***

# microchip1 1.5196 0.2669 5.695 1.24e-08 ***

# pure_crossdesigner 1.1408 0.7265 1.570 0.11638

# pure_crosspure -0.2709 0.2660 -1.019 0.30839

# pure_crossunknown 0.7511 0.6436 1.167 0.24318

# price_bin$2000+ -1.4796 0.4978 -2.972 0.00295 **

# price_bin$501 - $2000 -0.6402 0.2987 -2.143 0.03212 *

# price_binFree 0.3309 0.3173 1.043 0.29699

# price_binUnknown -1.0520 0.5732 -1.835 0.06646 .

# ---

# Signif. codes: 0 ‘***’ 0.001 ‘**’ 0.01 ‘*’ 0.05 ‘.’ 0.1 ‘ ’ 1

#

# (Dispersion parameter for binomial family taken to be 1)

#

# Null deviance: 528.93 on 498 degrees of freedom

# Residual deviance: 473.43 on 490 degrees of freedom

# AIC: 491.43

#

# Number of Fisher Scoring iterations: 5

desexraci7 <- confint(model_ra_desex7)

round(exp(cbind(model_ra_desex7$coef, desexraci7)), digits = 2)

# 2.5 % 97.5 %

# (Intercept) 0.16 0.09 0.28

# microchip1 4.57 2.75 7.86

# pure_crossdesigner 3.13 0.70 12.72

# pure_crosspure 0.76 0.45 1.29

# pure_crossunknown 2.12 0.57 7.41

# price_bin$2000+ 0.23 0.08 0.57

# price_bin$501 - $2000 0.53 0.29 0.94

# price_binFree 1.39 0.74 2.59

# price_binUnknown 0.35 0.10 0.98

lrtest(model_ra_desex7, model_ra_desex6)

# Likelihood ratio test

#

# Model 1: desexed ~ microchip + pure_cross + price_bin

# Model 2: desexed ~ microchip + pure_cross + age_bin + price_bin

# #Df LogLik Df Chisq Pr(>Chisq)

# 1 9 -236.72

# 2 12 -224.14 3 25.154 1.434e-05 ***

# ---

# Signif. codes: 0 ‘***’ 0.001 ‘**’ 0.01 ‘*’ 0.05 ‘.’ 0.1 ‘ ’ 1

#age is sig

#model 8: Model 6 minus price

model_ra_desex8 <- glm(desexed ~ microchip +

pure_cross +

age_bin, family = binomial,

data = dogads3desex)

summary(model_ra_desex8)

# Call:

# glm(formula = desexed ~ microchip + pure_cross + age_bin, family = binomial,

# data = dogads3desex)

#

# Deviance Residuals:

# Min 1Q Median 3Q Max

# -1.3594 -0.7094 -0.5413 -0.2625 2.6021

#

# Coefficients:

# Estimate Std. Error z value Pr(>|z|)

# (Intercept) -1.6809 0.3016 -5.574 2.49e-08 ***

# microchip1 1.5045 0.2670 5.636 1.74e-08 ***

# pure_crossdesigner 0.9698 0.6876 1.411 0.158370

# pure_crosspure -0.5952 0.2576 -2.310 0.020865 *

# pure_crossunknown 0.3717 0.6458 0.576 0.564912

# age_bin3 + years 0.5946 0.2800 2.124 0.033700 *

# age_bin6 months - 1 year -1.0750 0.3238 -3.320 0.000901 ***

# age_binUnknown -1.0638 0.4548 -2.339 0.019328 *

# ---

# Signif. codes: 0 ‘***’ 0.001 ‘**’ 0.01 ‘*’ 0.05 ‘.’ 0.1 ‘ ’ 1

#

# (Dispersion parameter for binomial family taken to be 1)

#

# Null deviance: 528.93 on 498 degrees of freedom

# Residual deviance: 461.38 on 491 degrees of freedom

# AIC: 477.38

#

# Number of Fisher Scoring iterations: 5

desexraci8 <- confint(model_ra_desex8)

round(exp(cbind(model_ra_desex8$coef, desexraci8)), digits = 2)

# 2.5 % 97.5 %

# (Intercept) 0.19 0.10 0.33

# microchip1 4.50 2.71 7.74

# pure_crossdesigner 2.64 0.63 9.95

# pure_crosspure 0.55 0.33 0.92

# pure_crossunknown 1.45 0.39 5.08

# age_bin3 + years 1.81 1.05 3.14

# age_bin6 months - 1 year 0.34 0.18 0.63

# age_binUnknown 0.35 0.13 0.80

# >

lrtest(model_ra_desex8, model_ra_desex6)

# Likelihood ratio test

#

# Model 1: desexed ~ microchip + pure_cross + age_bin

# Model 2: desexed ~ microchip + pure_cross + age_bin + price_bin

# #Df LogLik Df Chisq Pr(>Chisq)

# 1 8 -230.69

# 2 12 -224.14 4 13.099 0.0108 *

# ---

# Signif. codes: 0 ‘***’ 0.001 ‘**’ 0.01 ‘*’ 0.05 ‘.’ 0.1 ‘ ’ 1

#price is sig

#model 6 is the final model

model_ra_desex6_RR<-odds_to_rr(model_ra_desex6)

format(round(model_ra_desex6_RR,2),nsmall=2)

# RR lower.ci upper.ci

# (Intercept) 0.23 0.12 0.41

# microchip1 2.68 2.10 3.25

# pure_crossdesigner 2.47 0.96 3.74

# pure_crosspure 0.73 0.45 1.13

# pure_crossunknown 1.47 0.50 2.87

# age_bin3 + years 1.52 1.00 2.14

# age_bin6 months - 1 year 0.43 0.23 0.74

# age_binUnknown 0.45 0.18 0.94

# price_bin$2000+ 0.30 0.11 0.70

# price_bin$501 - $2000 0.70 0.41 1.14

# price_binFree 1.11 0.64 1.74

# price_binUnknown 0.41 0.12 1.02

###########interactions for microchipping###########

######offered by and desexed

modelmicrochip_explore <- glm(formula = microchip ~ offered_by + desexed,

family = binomial, data = dogads3chip)

summary(modelmicrochip_explore)

#

# Call:

# glm(formula = microchip ~ offered_by + desexed, family = binomial,

# data = dogads3chip)

#

# Deviance Residuals:

# Min 1Q Median 3Q Max

# -2.5335 -1.2476 0.4795 1.1088 1.1088

#

# Coefficients:

# Estimate Std. Error z value Pr(>|z|)

# (Intercept) 2.1052 0.1223 17.217 < 2e-16 ***

# offered_byOwner -1.9418 0.1399 -13.884 < 2e-16 ***

# desexed1 1.0629 0.2315 4.591 4.4e-06 ***

# ---

# Signif. codes: 0 ‘***’ 0.001 ‘**’ 0.01 ‘*’ 0.05 ‘.’ 0.1 ‘ ’ 1

#

# (Dispersion parameter for binomial family taken to be 1)

#

# Null deviance: 2012.2 on 1661 degrees of freedom

# Residual deviance: 1760.3 on 1659 degrees of freedom

# AIC: 1766.3

#

# Number of Fisher Scoring iterations: 4

#SIGNIFICANT.

explore_ci <- confint(modelmicrochip_explore)

round(exp(cbind(modelmicrochip_explore$coef, explore_ci)), digits = 2)

# 2.5 % 97.5 %

# (Intercept) 8.21 6.51 10.51

# offered_byOwner 0.14 0.11 0.19

# desexed1 2.89 1.87 4.64

#look at interaction

modelmicrochip_explore <- glm(formula = microchip ~ offered_by + desexed + offered_by*desexed,

family = binomial, data = dogads3chip)

# summary(modelmicrochip_explore)

# Call:

# glm(formula = microchip ~ offered_by + desexed + offered_by *

# desexed, family = binomial, data = dogads3chip)

#

# Deviance Residuals:

# Min 1Q Median 3Q Max

# -2.2649 -1.2456 0.4762 1.1107 1.1107

#

# Coefficients:

# Estimate Std. Error z value Pr(>|z|)

# (Intercept) 2.1197 0.1239 17.11 <2e-16 ***

# offered_byOwner -1.9608 0.1418 -13.83 <2e-16 ***

# desexed1 0.3652 0.7458 0.49 0.624

# offered_byOwner:desexed1 0.7522 0.7837 0.96 0.337

# ---

# Signif. codes: 0 ‘***’ 0.001 ‘**’ 0.01 ‘*’ 0.05 ‘.’ 0.1 ‘ ’ 1

#

# (Dispersion parameter for binomial family taken to be 1)

#

# Null deviance: 2012.2 on 1661 degrees of freedom

# Residual deviance: 1759.5 on 1658 degrees of freedom

# AIC: 1767.5

#

# Number of Fisher Scoring iterations: 4

#NOT SIGNIFICANT.

#########################look at interaction between offered by and age

modelmicrochip_explore3a <- glm(formula = microchip ~ age_bin + offered_by,

family = binomial, data = dogads3chip)

summary(modelmicrochip_explore3a)

explore_ci <- confint(modelmicrochip_explore3a)

round(exp(cbind(modelmicrochip_explore3a$coef, explore_ci)), digits = 2)

modelmicrochip_explore3 <- glm(formula = microchip ~ age_bin + offered_by + offered_by*age_bin,

family = binomial, data = dogads3chip)

summary(modelmicrochip_explore3)

#not significant.

#lets look with the breeder as reference

dogadsra$offered_by <- relevel(dogadsra$offered_by, ref= "Breeder")

table(dogadsra$offered_by)

modelmicrochip_explore4 <- glm(formula = microchip ~ age_bin + offered_by + offered_by*age_bin,

family = binomial, data = dogads3chip)

summary(modelmicrochip_explore4)

# Call:

# glm(formula = microchip ~ age_bin + offered_by + offered_by *

# age_bin, family = binomial, data = dogads3chip)

#

# Deviance Residuals:

# Min 1Q Median 3Q Max

# -2.2116 -1.1570 0.4531 0.9743 1.1980

#

# Coefficients:

# Estimate Std. Error z value Pr(>|z|)

# (Intercept) 2.2246 0.1861 11.956 < 2e-16 ***

# age_bin1 - 3 years -0.9253 0.4967 -1.863 0.0625 .

# age_bin3 + years -1.5315 0.6400 -2.393 0.0167 *

# age_bin6 months - 1 year -0.7777 0.5860 -1.327 0.1845

# age_bin8 weeks - 6 months 0.1304 0.2770 0.471 0.6377

# age_binUnknown -0.6152 0.6593 -0.933 0.3507

# offered_byOwner -1.7260 0.2255 -7.654 1.95e-14 ***

# age_bin1 - 3 years:offered_byOwner 0.3785 0.5358 0.706 0.4799

# age_bin3 + years:offered_byOwner 1.0763 0.6851 1.571 0.1162

# age_bin6 months - 1 year:offered_byOwner 0.4381 0.6258 0.700 0.4838

# age_bin8 weeks - 6 months:offered_byOwner -0.2963 0.3286 -0.902 0.3672

# age_binUnknown:offered_byOwner 0.8097 0.7589 1.067 0.2860

# ---

# Signif. codes: 0 ‘***’ 0.001 ‘**’ 0.01 ‘*’ 0.05 ‘.’ 0.1 ‘ ’ 1

#

# (Dispersion parameter for binomial family taken to be 1)

#

# Null deviance: 2012.2 on 1661 degrees of freedom

# Residual deviance: 1763.3 on 1650 degrees of freedom

# AIC: 1787.3

#

# Number of Fisher Scoring iterations: 4

######################interaction between vaccination and age

#look at interaction

modelmicrochip_explore8 <- glm(formula = microchip ~ age_bin + vaccination + age_bin*vaccination,

family = binomial, data = dogads3chip)

summary(modelmicrochip_explore8)

# Call:

# glm(formula = microchip ~ age_bin + vaccination + age_bin * vaccination,

# family = binomial, data = dogads3chip)

#

# Deviance Residuals:

# Min 1Q Median 3Q Max

# -2.4478 -0.5469 0.4163 0.4616 2.2974

#

# Coefficients:

# Estimate Std. Error z value Pr(>|z|)

# (Intercept) -1.67765 0.26422 -6.349 2.16e-10 ***

# age_bin1 - 3 years 0.12302 0.38803 0.317 0.7512

# age_bin3 + years 0.71257 0.39512 1.803 0.0713 .

# age_bin6 months - 1 year 0.19604 0.43879 0.447 0.6550

# age_bin8 weeks - 6 months -0.14690 0.38371 -0.383 0.7018

# age_binUnknown -0.88730 1.07086 -0.829 0.4073

# vaccinationyes 4.08008 0.31157 13.095 < 2e-16 ***

# age_bin1 - 3 years:vaccinationyes -1.04384 0.48907 -2.134 0.0328 *

# age_bin3 + years:vaccinationyes -1.39734 0.59320 -2.356 0.0185 *

# age_bin6 months - 1 year:vaccinationyes -1.17136 0.53730 -2.180 0.0293 *

# age_bin8 weeks - 6 months:vaccinationyes -0.07019 0.44516 -0.158 0.8747

# age_binUnknown:vaccinationyes 1.42931 1.30394 1.096 0.2730

# ---

# Signif. codes: 0 ‘***’ 0.001 ‘**’ 0.01 ‘*’ 0.05 ‘.’ 0.1 ‘ ’ 1

#

# (Dispersion parameter for binomial family taken to be 1)

#

# Null deviance: 2012.2 on 1661 degrees of freedom

# Residual deviance: 1218.2 on 1650 degrees of freedom

# AIC: 1242.2

#

# Number of Fisher Scoring iterations: 5

explore_ci <- confint(modelmicrochip_explore8)

round(exp(cbind(modelmicrochip_explore8$coef, explore_ci)), digits = 2)

#############interactions for desexing##############

#interactions between seller and age?

modeldesex_explore <- glm(formula = desexed ~ offered_by + age_bin + offered_by*age_bin,

family = binomial, data = dogads3desex)

summary(modeldesex_explore)

# Call:

# glm(formula = desexed ~ offered_by + age_bin + offered_by * age_bin,

# family = binomial, data = dogads3desex)

#

# Deviance Residuals:

# Min 1Q Median 3Q Max

# -0.96057 -0.78481 -0.53839 -0.00036 2.46760

#

# Coefficients:

# Estimate Std. Error z value Pr(>|z|)

# (Intercept) -1.7918 0.5401 -3.318 0.000908 ***

# offered_byOwner 0.7719 0.5680 1.359 0.174115

# age_bin3 + years 1.0986 0.8165 1.346 0.178457

# age_bin6 months - 1 year -1.2040 1.1583 -1.039 0.298606

# age_binUnknown -14.7743 565.5784 -0.026 0.979160

# offered_byOwner:age_bin3 + years -0.6129 0.8627 -0.710 0.477453

# offered_byOwner:age_bin6 months - 1 year 0.3657 1.2002 0.305 0.760623

# offered_byOwner:age_binUnknown 14.3728 565.5786 0.025 0.979726

# ---

# Signif. codes: 0 ‘***’ 0.001 ‘**’ 0.01 ‘*’ 0.05 ‘.’ 0.1 ‘ ’ 1

#

# (Dispersion parameter for binomial family taken to be 1)

#

# Null deviance: 528.93 on 498 degrees of freedom

# Residual deviance: 494.65 on 491 degrees of freedom

# AIC: 510.65

#

# Number of Fisher Scoring iterations: 15

#######fishers test for designer dgs and age###

#to see if sig

test <- dogads3

#remove unclear - too few counts

test <- test[!(test$pure_cross == "unknown"), ]

#amalgamate ages

#combine age bins 3-7 and 7 up

test <-

test %>%

mutate(

age_bin = case_when(

age < 8*7 ~ "< 8 weeks old",

age < 6 * 4 * 7 ~ "8 weeks - 6 months",

age < 365 ~ "6 months - 1 year",

age < 3 * 365 ~ "1 - 3 years",

age < 7 * 365 ~ "3 + years",

is.na(age) ~ "Unknown",

TRUE ~ "3 + years"

)

)

table(test$age_bin)

table(test$pure_cross)

table(test$pure_cross, test$age_bin)

pure_chi <- table(test$pure_cross, test$age_bin)

chisq.test(pure_chi)

#################################12/11 BID MODEL################

#NOTE THIS WAS THE FINAL ONES USED.

#NEED TO CHANGE THE DATASET

#create new one to start with.

dogadsID2 <- dogads3

dogadsID2

#select NSW ads and delete rows

dogadsID2<- dogadsID2[!(dogadsID2$state == "NSW"), ]

dogadsID2

#575 DELETED from dataset

#select act ads and delete rows

dogadsID2<- dogadsID2[!(dogadsID2$state == "ACT"), ]

dogadsID2

#7 DELETED from dataset

#select TAS ads and delete rows

dogadsID2<- dogadsID2[!(dogadsID2$state == "TAS"), ]

dogadsID2

#58 DELETED from dataset

#select WA ads and delete rows

dogadsID2<- dogadsID2[!(dogadsID2$state == "WA"), ]

dogadsID2

#138 DELETED from dataset

#select NT ads and delete rows

dogadsID2<- dogadsID2[!(dogadsID2$state == "NT"), ]

dogadsID2

#20 DELETED from dataset

#select na ads and delete rows

dogadsID2<- dogadsID2[!(dogadsID2$state == "na"), ]

dogadsID2

#164 DELETED from dataset

#total 773 ads to work from

table(dogadsID2$state_registered)

#make ads that are 'unclear' on their state registration a "no"

dogadsID2$state_registered [dogadsID2$state_registered == "unclear"] <- "no"

#done

#773 ads to work from

#confirming dataset correct and ready to go

table(dogadsID2$state_registered, dogadsID2$state)

#now we change the microchip value unclear to "no" as the counts will be too small.

dogadsID2$microchip [dogadsID2$microchip == "unclear"] <- "no"

table(dogadsID2$microchip)

#done.

#now we change the vacc value unclear to "no" as the counts will be too small.

dogadsID2$vaccination [dogadsID2$vaccination == "unclear"] <- "no"

table(dogadsID2$vaccination)

#done. but need to get rid of the factor 'unclear'.

dogadsID2 <- dogadsID2[ dogadsID2$vaccination != "unclear", , drop=FALSE]; dogadsID2$vaccination <- factor(dogadsID2$vaccination)

#done

#change state registered from yes and no to 1 and 0

dogadsID2$state_registered [dogadsID2$state_registered == "yes"] <- "1"

dogadsID2$state_registered [dogadsID2$state_registered == "no"] <- "0"

table(dogadsID2$state_registered, dogadsID2$state)

#change ANKC, to "breeder org, yes or no" - as numbers and % for ANKC and

#other are almost identical.

#so ankc and Other = "yes"

#none = "no"

dogadsID2

#now we change the value ankc to "yes"

dogadsID2$ankc_registered [dogadsID2$ankc_registered == "ankc"] <- "yes"

#check if the change worked.

table(dogadsID2$ankc_registered)

#change value other to "yes"

dogadsID2$ankc_registered [dogadsID2$ankc_registered == "other"] <- "yes"

#check if the change worked.

table(dogadsID2$ankc_registered)

#correct.

#change column name to "breeder organisation affiliation" as now has ALL org groups not just ankc.

dogadsID2 <- dogadsID2 %>%

rename(

"breeder_org_affiliation" = ankc_registered

)

#remove unclear to "none"

dogadsID2$breeder_org_affiliation [dogadsID2$breeder_org_affiliation == "unclear"] <- "none"

table(dogadsID2$breeder_org_affiliation)

#put price into bins.

dogadsID2 <-

dogadsID2 %>%

mutate(

price_bin = case_when(

price == 0 ~ "Free",

price < 501 ~ "$1 - $500",

price < 2001 ~ "$501 - $2000",

price > 2000 ~ "$2000+",

is.na(price) ~ "Unknown",

)

)

table(dogadsID2$price_bin)

#done

#age bins amalgamating so 3+

dogadsID2 <-

dogadsID2 %>%

mutate(

age_bin = case_when(

age < 8*7 ~ "< 8 weeks old",

age < 6 * 4 * 7 ~ "8 weeks - 6 months",

age < 365 ~ "6 months - 1 year",

age < 3 * 365 ~ "1 - 3 years",

age < 7 * 365 ~ "3 + years",

is.na(age) ~ "Unknown",

TRUE ~ "3 + years"

)

)

table(dogadsID2$age_bin)

#done

#unknown age is causing issues due to low counts. Remove.

dogadsID2 <- dogadsID2 %>% filter(!(age_bin == "Unknown"))

dogadsID2

table(dogadsID2$age_bin)

#worked

#remove all shelter dogs due to interactions

#remove all QLD older than 682 days.

dogadsID2 <- dogadsID2 %>% filter(!(offered_by == "Shelter/Rescue"))

dogadsID2

table(dogadsID2$offered_by)

#need to turn all independant variables into categorical variables.

#microchip

#desexed

#pure/cross

#offered by

#vaccinatin

#breeder org affiliation

#change microchip to categorical

dogadsID2$microchip <- factor(dogadsID$microchip)

#check if worked - yes

dogadsID2

#change desexed to categorical

dogadsID2$desexed <- factor(dogadsID$desexed)

#check if worked - yes

dogadsID2

#change pure_cross to categorical

dogadsID2$pure_cross <- factor(dogadsID2$pure_cross)

#check if worked - yes

dogadsID2

#change offered by to categorical

dogadsID2$offered_by <- factor(dogadsID2$offered_by)

#check if worked - yes

dogadsID

#change vaccination to categorical

dogadsID2$vaccination <- factor(dogadsID2$vaccination)

#check if worked - yes

dogadsID2

#change breeder org to categorical

dogadsID2$breeder_org_affiliation <- factor(dogadsID2$breeder_org_affiliation)

#check if worked - yes

dogadsID2

#change state registered org to categorical

dogadsID2$state_registered <- factor(dogadsID2$state_registered)

#check if worked - yes

dogadsID2

#now neeed to remove all SA dogs born prior to July 1st 2018.

#remove all dogs older than 268 days. #expecting to have 84 ads left in SA

dogadsID2 <- dogadsID2 %>% filter(!(state == "SA" & age > 268))

#also removes na ages

table(dogadsID2$state)

#remove qld dogs born prior to May 26 2017

#remove all QLD older than 682 days.

dogadsID2 <- dogadsID2 %>% filter(!(state == "QLD" & age > 682))

dogadsID2

#also removes na ages

table(dogadsID2$state)

# dogadsID2 is now new dataset for BID univariate and regression version 2

#saved

write_rds(dogadsID2,"C:/Users/Sofia Costa/OneDrive/Adelaide University Hons/Honours Program/R/dogadsID2.rds")

#load

dogadsID2 <- read_rds("C:/Users/Sofia Costa/OneDrive/Adelaide University Hons/Honours Program/R/dogadsID2.rds")

dogadsID2

table(dogadsID2$state)

######################UNIVARIATE ANALYSIS - BID

########################microchipping

#note we changed unclear to "no" in microchipping as counts are too small.

BID_chip <- table(dogadsID2$microchip, dogadsID2$state_registered)

BID_chip

# # 0 1

# no 81 40

# yes 194 333

chisq.test(BID_chip)

# Pearson's Chi-squared test with Yates' continuity correction

#

# data: BID_chip

# X-squared = 35.347, df = 1, p-value = 2.759e-09

modelBID_chip_ra <- glm(state_registered ~ microchip, family = binomial, data = dogadsID2)

summary(modelBID_chip_ra)

#

# Call:

# glm(formula = state_registered ~ microchip, family = binomial,

# data = dogadsID2)

#

# Deviance Residuals:

# Min 1Q Median 3Q Max

# -1.4138 -1.4138 0.9582 0.9582 1.4879

#

# Coefficients:

# Estimate Std. Error z value Pr(>|z|)

# (Intercept) -0.7056 0.1933 -3.651 0.000261 ***

# microchipyes 1.2459 0.2133 5.840 5.21e-09 ***

# ---

# Signif. codes: 0 ‘***’ 0.001 ‘**’ 0.01 ‘*’ 0.05 ‘.’ 0.1 ‘ ’ 1

#

# (Dispersion parameter for binomial family taken to be 1)

#

# Null deviance: 883.44 on 647 degrees of freedom

# Residual deviance: 847.05 on 646 degrees of freedom

# AIC: 851.05

#

# Number of Fisher Scoring iterations: 4

BID_ra_ci <- confint(modelBID_chip_ra)

round(exp(cbind(modelBID_chip_ra$coef, BID_ra_ci)), digits = 2)

# # 2.5 % 97.5 %

# (Intercept) 0.49 0.33 0.72

# microchipyes 3.48 2.30 5.3

modelpure_ra_RR<-odds_to_rr(modelBID_chip_ra)

format(round(modelpure_ra_RR,2),nsmall=2)

# # RR lower.ci upper.ci

# (Intercept) 0.70 0.54 0.86

# microchipyes 1.43 1.32 1.53

##########vaccinated

BID_vac <- table(dogadsID2$vaccination, dogadsID2$state_registered)

BID_vac

# # 0 1

# no 83 38

# yes 192 335

chisq.test(BID_vac)

#

#

# Pearson's Chi-squared test with Yates' continuity correction

#

# data: BID_vac

# X-squared = 40.364, df = 1, p-value = 2.108e-10

modelBID_vac_ra <- glm(state_registered ~ vaccination, family = binomial, data = dogadsID2)

summary(modelBID_vac_ra)

# #Call:

# glm(formula = state_registered ~ vaccination, family = binomial,

# data = dogadsID2)

#

# Deviance Residuals:

# Min 1Q Median 3Q Max

# -1.4211 -1.4211 0.9519 0.9519 1.5220

#

# Coefficients:

# Estimate Std. Error z value Pr(>|z|)

# (Intercept) -0.7813 0.1959 -3.989 6.64e-05 ***

# vaccinationyes 1.3379 0.2158 6.200 5.63e-10 ***

# ---

# Signif. codes: 0 ‘***’ 0.001 ‘**’ 0.01 ‘*’ 0.05 ‘.’ 0.1 ‘ ’ 1

#

# (Dispersion parameter for binomial family taken to be 1)

#

# Null deviance: 883.44 on 647 degrees of freedom

# Residual deviance: 841.88 on 646 degrees of freedom

# AIC: 845.88

#

# Number of Fisher Scoring iterations: 4

#

BID_vac_ci <- confint(modelBID_vac_ra)

round(exp(cbind(modelBID_vac_ra$coef, BID_vac_ci)), digits = 2)

#

# 2.5 % 97.5 %

# (Intercept) 0.46 0.31 0.67

# vaccinationyes 3.81 2.51 5.87

modelvac_ra_RR<-odds_to_rr(modelBID_vac_ra)

format(round(modelvac_ra_RR,2),nsmall=2)

# RR lower.ci upper.ci

# (Intercept) 0.67 0.51 0.83

# vaccinationyes 1.46 1.34 1.54

#########pure _cross

BID_pure <- table(dogadsID2$pure_cross, dogadsID2$state_registered)

BID_pure

# # 0 1

# cross 80 82

# designer 19 46

# pure 166 244

# unknown 10 1

>

fisher.test(BID_pure)

# Fisher's Exact Test for Count Data

#

# data: BID_pure

# p-value = 0.0002027

# alternative hypothesis: two.sided

modelBID_pure_ra <- glm(state_registered ~ pure_cross, family = binomial, data = dogadsID2)

summary(modelBID_pure_ra)

# Call:

# glm(formula = state_registered ~ pure_cross, family = binomial,

# data = dogadsID2)

#

# Deviance Residuals:

# Min 1Q Median 3Q Max

# -1.568 -1.345 1.019 1.019 2.190

#

# Coefficients:

# Estimate Std. Error z value Pr(>|z|)

# (Intercept) 0.02469 0.15715 0.157 0.87514

# pure_crossdesigner 0.85951 0.31475 2.731 0.00632 **

# pure_crosspure 0.36049 0.18659 1.932 0.05337 .

# pure_crossunknown -2.32728 1.06030 -2.195 0.02817 *

# ---

# Signif. codes: 0 ‘***’ 0.001 ‘**’ 0.01 ‘*’ 0.05 ‘.’ 0.1 ‘ ’ 1

#

# (Dispersion parameter for binomial family taken to be 1)

#

# Null deviance: 883.44 on 647 degrees of freedom

# Residual deviance: 863.25 on 644 degrees of freedom

# AIC: 871.25

#

# Number of Fisher Scoring iterations: 4

BID_pure_ci <- confint(modelBID_pure_ra)

round(exp(cbind(modelBID_pure_ra$coef, BID_pure_ci)), digits = 2)

#

# 2.5 % 97.5 %

# (Intercept) 1.03 0.75 1.40

# pure_crossdesigner 2.36 1.29 4.45

# pure_crosspure 1.43 0.99 2.07

# pure_crossunknown 0.10 0.01 0.5

modelpure_ra_RR<-odds_to_rr(modelBID_pure_ra)

format(round(modelpure_ra_RR,2),nsmall=2)

# # # RR lower.ci upper.ci

# (Intercept) 1.01 0.88 1.14

# pure_crossdesigner 1.32 1.11 1.49

# pure_crosspure 1.15 1.00 1.28

# pure_crossunknown 0.20 0.01 0.72

########seller (offered by)

BID_seller <- table(dogadsID2$offered_by, dogadsID2$state_registered)

BID_seller

# # 0 1

# Breeder 93 289

# Owner 182 84

chisq.test(BID_seller)

# # Pearson's Chi-squared test with Yates' continuity correction

#

# data: BID_seller

# X-squared = 122.9, df = 1, p-value < 2.2e-16

modelBID_seller_ra <- glm(state_registered ~ offered_by, family = binomial, data = dogadsID2)

summary(modelBID_seller_ra)

#

#

# Call:

# glm(formula = state_registered ~ offered_by, family = binomial,

# data = dogadsID2)

#

# Deviance Residuals:

# Min 1Q Median 3Q Max

# -1.6810 -0.8712 0.7470 0.7470 1.5183

#

# Coefficients:

# Estimate Std. Error z value Pr(>|z|)

# (Intercept) 1.1338 0.1192 9.511 <2e-16 ***

# offered_byOwner -1.9070 0.1778 -10.726 <2e-16 ***

# ---

# Signif. codes: 0 ‘***’ 0.001 ‘**’ 0.01 ‘*’ 0.05 ‘.’ 0.1 ‘ ’ 1

#

# (Dispersion parameter for binomial family taken to be 1)

#

# Null deviance: 883.44 on 647 degrees of freedom

# Residual deviance: 755.83 on 646 degrees of freedom

# AIC: 759.83

#

# Number of Fisher Scoring iterations: 4

BID_seller_ci <- confint(modelBID_seller_ra)

round(exp(cbind(modelBID_seller_ra$coef, BID_seller_ci)), digits = 2)

# 2.5 % 97.5 %

# (Intercept) 3.11 2.47 3.95

# offered_byOwner 0.15 0.10 0.21

modelseller_ra_RR<-odds_to_rr(modelBID_seller_ra)

format(round(modelseller_ra_RR,2),nsmall=2)

# RR lower.ci upper.ci

# (Intercept) 1.40 1.34 1.46

# offered_byOwner 0.29 0.22 0.38

#############breeder org affiliation

BID_org <- table(dogadsID2$breeder_org_affiliation, dogadsID2$state_registered)

BID_org

# # # 0 1

# none 217 204

# yes 58 169

chisq.test(BID_org)

#

# Pearson's Chi-squared test with Yates' continuity correction

#

# data: BID_org

# X-squared = 39.734, df = 1, p-value = 2.91e-10

modelBID_org_ra <- glm(state_registered ~ breeder_org_affiliation, family = binomial, data = dogadsID2)

summary(modelBID_org_ra)

#

#

# Call:

# glm(formula = state_registered ~ breeder_org_affiliation, family = binomial,

# data = dogadsID2)

#

# Deviance Residuals:

# Min 1Q Median 3Q Max

# -1.6520 -1.1513 0.7682 1.2038 1.2038

#

# Coefficients:

# Estimate Std. Error z value Pr(>|z|)

# (Intercept) -0.06178 0.09752 -0.633 0.526

# breeder_org_affiliationyes 1.13123 0.18074 6.259 3.88e-10 ***

# ---

# Signif. codes: 0 ‘***’ 0.001 ‘**’ 0.01 ‘*’ 0.05 ‘.’ 0.1 ‘ ’ 1

#

# (Dispersion parameter for binomial family taken to be 1)

#

# Null deviance: 883.44 on 647 degrees of freedom

# Residual deviance: 841.24 on 646 degrees of freedom

# AIC: 845.24

#

# Number of Fisher Scoring iterations: 4

BID_org_ci <- confint(modelBID_org_ra)

round(exp(cbind(modelBID_org_ra$coef, BID_org_ci)), digits = 2)

# 2.5 % 97.5 %

# (Intercept) 0.94 0.78 1.14

# breeder_org_affiliationyes 3.10 2.19 4.44

modelorg_ra_RR<-odds_to_rr(modelBID_org_ra)

format(round(modelorg_ra_RR,2),nsmall=2)

# RR lower.ci upper.ci

# (Intercept) 0.97 0.89 1.05

# breeder_org_affiliationyes 1.40 1.30 1.49

##############age

BID_age <- table(dogadsID2$age_bin, dogadsID2$state_registered)

BID_age

#

# 0 1

# < 8 weeks old 61 213

# 1 - 3 years 56 8

# 3 + years 12 1

# 6 months - 1 year 37 8

# 8 weeks - 6 months 109 143

# >

chisq.test(BID_age)

# #Pearson's Chi-squared test

#

# data: BID_age

# X-squared = 141.32, df = 4, p-value < 2.2e-16

modelBID_age_ra <- glm(state_registered ~ age_bin, family = binomial, data = dogadsID2)

summary(modelBID_age_ra)

#

#

# Call:

# glm(formula = state_registered ~ age_bin, family = binomial,

# data = dogadsID2)

#

# Deviance Residuals:

# Min 1Q Median 3Q Max

# -1.7333 -1.2947 0.7097 0.7097 2.2649

#

# Coefficients:

# Estimate Std. Error z value Pr(>|z|)

# (Intercept) 1.2504 0.1452 8.611 < 2e-16 ***

# age_bin1 - 3 years -3.1963 0.4049 -7.894 2.92e-15 ***

# age_bin3 + years -3.7353 1.0501 -3.557 0.000375 ***

# age_bin6 months - 1 year -2.7819 0.4161 -6.686 2.29e-11 ***

# age_bin8 weeks - 6 months -0.9789 0.1930 -5.072 3.94e-07 ***

# ---

# Signif. codes: 0 ‘***’ 0.001 ‘**’ 0.01 ‘*’ 0.05 ‘.’ 0.1 ‘ ’ 1

#

# (Dispersion parameter for binomial family taken to be 1)

#

# Null deviance: 883.44 on 647 degrees of freedom

# Residual deviance: 732.70 on 643 degrees of freedom

# AIC: 742.7

#

# Number of Fisher Scoring iterations: 4

BID_age_ci <- confint(modelBID_age_ra)

round(exp(cbind(modelBID_age_ra$coef, BID_age_ci)), digits = 2)

#

# 2.5 % 97.5 %

# (Intercept) 3.49 2.65 4.68

# age_bin1 - 3 years 0.04 0.02 0.09

# age_bin3 + years 0.02 0.00 0.12

# age_bin6 months - 1 year 0.06 0.03 0.13

# age_bin8 weeks - 6 months 0.38 0.26 0.55

modelage_ra_RR<-odds_to_rr(modelBID_age_ra)

format(round(modelage_ra_RR,2),nsmall=2)

#

# RR lower.ci upper.ci

# (Intercept) 1.43 1.36 1.50

# age_bin1 - 3 years 0.09 0.04 0.18

# age_bin3 + years 0.05 0.00 0.25

# age_bin6 months - 1 year 0.13 0.06 0.27

# age_bin8 weeks - 6 months 0.59 0.45 0.74

###############price

BID_price <- table(dogadsID2$price_bin, dogadsID2$state_registered)

BID_price

#

# 0 1

# $1 - $500 93 72

# $2000+ 64 113

# $501 - $2000 82 180

# Free 23 2

# Unknown 13 6

chisq.test(BID_price)

# # Pearson's Chi-squared test

#

# data: BID_price

# X-squared = 59.657, df = 4, p-value = 3.425e-12

modelBID_price_ra <- glm(state_registered ~ price_bin, family = binomial, data = dogadsID2)

summary(modelBID_price_ra)

#

# # call:

# glm(formula = state_registered ~ price_bin, family = binomial,

# data = dogadsID2)

#

# Deviance Residuals:

# Min 1Q Median 3Q Max

# -1.5242 -1.0708 0.8665 0.9474 2.2475

#

# Coefficients:

# Estimate Std. Error z value Pr(>|z|)

# (Intercept) -0.2559 0.1570 -1.630 0.103018

# price_bin$2000+ 0.8244 0.2216 3.720 0.000199 ***

# price_bin$501 - $2000 1.0422 0.2059 5.062 4.16e-07 ***

# price_binFree -2.1864 0.7533 -2.902 0.003704 **

# price_binUnknown -0.5173 0.5179 -0.999 0.317922

# ---

# Signif. codes: 0 ‘***’ 0.001 ‘**’ 0.01 ‘*’ 0.05 ‘.’ 0.1 ‘ ’ 1

#

# (Dispersion parameter for binomial family taken to be 1)

#

# Null deviance: 883.44 on 647 degrees of freedom

# Residual deviance: 820.97 on 643 degrees of freedom

# AIC: 830.97

#

# Number of Fisher Scoring iterations: 4

BID_price_ci <- confint(modelBID_price_ra)

round(exp(cbind(modelBID_price_ra$coef, BID_price_ci)), digits = 2)

# 2.5 % 97.5 %

# (Intercept) 0.77 0.57 1.05

# price_bin$2000+ 2.28 1.48 3.53

# price_bin$501 - $2000 2.84 1.90 4.26

# price_binFree 0.11 0.02 0.40

# price_binUnknown 0.60 0.20 1.59

modelprice_ra_RR<-odds_to_rr(modelBID_price_ra)

format(round(modelprice_ra_RR,2),nsmall=2)

# RR lower.ci upper.ci

# (Intercept) 0.89 0.76 1.02

# price_bin$2000+ 1.31 1.16 1.44

# price_bin$501 - $2000 1.38 1.25 1.48

# price_binFree 0.23 0.04 0.61

# price_binUnknown 0.78 0.37 1.19

#

#########################NEW NOV REGRESSION MODEL - BID

#WITH ALL VARIABLES -

model_BID <- glm(state_registered ~ microchip + vaccination +

pure_cross + offered_by + breeder_org_affiliation +

age_bin + price_bin, family = binomial,

data = dogadsID2)

summary(model_BID)

# #Call:

# glm(formula = state_registered ~ microchip + vaccination + pure_cross +

# offered_by + breeder_org_affiliation + age_bin + price_bin,

# family = binomial, data = dogadsID2)

#

# Deviance Residuals:

# Min 1Q Median 3Q Max

# -2.2990 -0.7057 0.4625 0.7827 2.5515

#

# Coefficients:

# Estimate Std. Error z value Pr(>|z|)

# (Intercept) 1.1091 0.3756 2.953 0.00315 **

# microchipyes 0.3704 0.3258 1.137 0.25563

# vaccinationyes 0.4275 0.3213 1.331 0.18333

# pure_crossdesigner 0.2743 0.3968 0.691 0.48939

# pure_crosspure 0.1427 0.2617 0.545 0.58573

# pure_crossunknown -1.7591 1.1906 -1.477 0.13955

# offered_byOwner -1.3715 0.2436 -5.629 1.81e-08 ***

# breeder_org_affiliationyes 0.3873 0.2683 1.444 0.14887

# age_bin1 - 3 years -2.5280 0.4410 -5.732 9.94e-09 ***

# age_bin3 + years -3.5291 1.1345 -3.111 0.00187 **

# age_bin6 months - 1 year -2.1971 0.4500 -4.882 1.05e-06 ***

# age_bin8 weeks - 6 months -1.1556 0.2140 -5.401 6.64e-08 ***

# price_bin$2000+ -0.6985 0.3636 -1.921 0.05473 .

# price_bin$501 - $2000 0.1320 0.2873 0.459 0.64601

# price_binFree -0.5679 0.8506 -0.668 0.50433

# price_binUnknown -0.7497 0.5963 -1.257 0.20862

# ---

# Signif. codes: 0 ‘***’ 0.001 ‘**’ 0.01 ‘*’ 0.05 ‘.’ 0.1 ‘ ’ 1

#

# (Dispersion parameter for binomial family taken to be 1)

#

# Null deviance: 883.44 on 647 degrees of freedom

# Residual deviance: 640.79 on 632 degrees of freedom

# AIC: 672.79

#

# Number of Fisher Scoring iterations: 5

BIN_dci <- confint(model_BID)

round(exp(cbind(model_BID$coef, BIN_dci)), digits = 2)

# 2.5 % 97.5 %

# (Intercept) 3.03 1.46 6.41

# microchipyes 1.45 0.76 2.75

# vaccinationyes 1.53 0.82 2.88

# pure_crossdesigner 1.32 0.61 2.90

# pure_crosspure 1.15 0.69 1.92

# pure_crossunknown 0.17 0.01 1.27

# offered_byOwner 0.25 0.16 0.41

# breeder_org_affiliationyes 1.47 0.87 2.50

# age_bin1 - 3 years 0.08 0.03 0.18

# age_bin3 + years 0.03 0.00 0.19

# age_bin6 months - 1 year 0.11 0.04 0.26

# age_bin8 weeks - 6 months 0.31 0.21 0.48

# price_bin$2000+ 0.50 0.24 1.01

# price_bin$501 - $2000 1.14 0.65 2.00

# price_binFree 0.57 0.08 2.54

# price_binUnknown 0.47 0.14 1.49

#model 2: model 1 minus chip

model_BID_2 <- glm(state_registered ~ vaccination +

pure_cross + offered_by + breeder_org_affiliation +

age_bin + price_bin, family = binomial,

data = dogadsID2)

summary(model_BID_2)

# #Call:

# glm(formula = state_registered ~ vaccination + pure_cross + offered_by +

# breeder_org_affiliation + age_bin + price_bin, family = binomial,

# data = dogadsID2)

#

# Deviance Residuals:

# Min 1Q Median 3Q Max

# -2.2932 -0.7292 0.4660 0.7866 2.5285

#

# Coefficients:

# Estimate Std. Error z value Pr(>|z|)

# (Intercept) 1.2206 0.3623 3.369 0.000755 ***

# vaccinationyes 0.6237 0.2703 2.308 0.021015 *

# pure_crossdesigner 0.2883 0.3968 0.727 0.467491

# pure_crosspure 0.1284 0.2611 0.492 0.622875

# pure_crossunknown -1.8302 1.1850 -1.545 0.122454

# offered_byOwner -1.3945 0.2425 -5.750 8.91e-09 ***

# breeder_org_affiliationyes 0.3889 0.2684 1.449 0.147349

# age_bin1 - 3 years -2.5155 0.4401 -5.716 1.09e-08 ***

# age_bin3 + years -3.4669 1.1395 -3.042 0.002347 **

# age_bin6 months - 1 year -2.2066 0.4522 -4.880 1.06e-06 ***

# age_bin8 weeks - 6 months -1.1510 0.2135 -5.392 6.97e-08 ***

# price_bin$2000+ -0.6359 0.3583 -1.775 0.075957 .

# price_bin$501 - $2000 0.1928 0.2816 0.685 0.493455

# price_binFree -0.5940 0.8493 -0.699 0.484306

# price_binUnknown -0.6824 0.5921 -1.153 0.249111

# ---

# Signif. codes: 0 ‘***’ 0.001 ‘**’ 0.01 ‘*’ 0.05 ‘.’ 0.1 ‘ ’ 1

#

# (Dispersion parameter for binomial family taken to be 1)

#

# Null deviance: 883.44 on 647 degrees of freedom

# Residual deviance: 642.08 on 633 degrees of freedom

# AIC: 672.08

#

# Number of Fisher Scoring iterations: 5

BIN_dci <- confint(model_BID_2)

round(exp(cbind(model_BID_2$coef, BIN_dci)), digits = 2)

# 2.5 % 97.5 %

# (Intercept) 3.39 1.68 6.99

# vaccinationyes 1.87 1.10 3.18

# pure_crossdesigner 1.33 0.62 2.94

# pure_crosspure 1.14 0.68 1.89

# pure_crossunknown 0.16 0.01 1.18

# offered_byOwner 0.25 0.15 0.40

# breeder_org_affiliationyes 1.48 0.87 2.51

# age_bin1 - 3 years 0.08 0.03 0.18

# age_bin3 + years 0.03 0.00 0.21

# age_bin6 months - 1 year 0.11 0.04 0.26

# age_bin8 weeks - 6 months 0.32 0.21 0.48

# price_bin$2000+ 0.53 0.26 1.06

# price_bin$501 - $2000 1.21 0.70 2.10

# price_binFree 0.55 0.08 2.48

# price_binUnknown 0.51 0.15 1.58

lrtest(model_BID, model_BID_2)

Likelihood ratio test

# Likelihood ratio test

#

# Model 1: state_registered ~ microchip + vaccination + pure_cross + offered_by +

# breeder_org_affiliation + age_bin + price_bin

# Model 2: state_registered ~ vaccination + pure_cross + offered_by + breeder_org_affiliation +

# age_bin + price_bin

# #Df LogLik Df Chisq Pr(>Chisq)

# 1 16 -320.40

# 2 15 -321.04 -1 1.2925 0.2556

#MICROCHIP IS NOT SIG

#model 3: model 2 minus vaccination

model_BID_3 <- glm(state_registered ~ pure_cross + offered_by + breeder_org_affiliation +

age_bin + price_bin, family = binomial,

data = dogadsID2)

summary(model_BID_3)

# Call:

# glm(formula = state_registered ~ pure_cross + offered_by + breeder_org_affiliation +

# age_bin + price_bin, family = binomial, data = dogadsID2)

#

# Deviance Residuals:

# Min 1Q Median 3Q Max

# -2.2875 -0.8257 0.4823 0.8064 2.4027

#

# Coefficients:

# Estimate Std. Error z value Pr(>|z|)

# (Intercept) 1.6933 0.3027 5.594 2.21e-08 ***

# pure_crossdesigner 0.3342 0.3936 0.849 0.39584

# pure_crosspure 0.1810 0.2581 0.701 0.48325

# pure_crossunknown -1.8421 1.1690 -1.576 0.11507

# offered_byOwner -1.4580 0.2402 -6.069 1.29e-09 ***

# breeder_org_affiliationyes 0.4475 0.2664 1.680 0.09300 .

# age_bin1 - 3 years -2.5860 0.4399 -5.879 4.13e-09 ***

# age_bin3 + years -3.4703 1.1348 -3.058 0.00223 **

# age_bin6 months - 1 year -2.2243 0.4492 -4.952 7.34e-07 ***

# age_bin8 weeks - 6 months -1.1363 0.2120 -5.361 8.28e-08 ***

# price_bin$2000+ -0.6332 0.3548 -1.785 0.07430 .

# price_bin$501 - $2000 0.2187 0.2786 0.785 0.43255

# price_binFree -0.6593 0.8433 -0.782 0.43431

# price_binUnknown -0.7029 0.5885 -1.194 0.23230

# ---

# Signif. codes: 0 ‘***’ 0.001 ‘**’ 0.01 ‘*’ 0.05 ‘.’ 0.1 ‘ ’ 1

#

# (Dispersion parameter for binomial family taken to be 1)

#

# Null deviance: 883.44 on 647 degrees of freedom

# Residual deviance: 647.44 on 634 degrees of freedom

# AIC: 675.44

#

# Number of Fisher Scoring iterations: 5

BIN_dci <- confint(model_BID_3)

round(exp(cbind(model_BID_3$coef, BIN_dci)), digits = 2)

# 2.5 % 97.5 %

# (Intercept) 5.44 3.05 10.01

# pure_crossdesigner 1.40 0.65 3.06

# pure_crosspure 1.20 0.72 1.99

# pure_crossunknown 0.16 0.01 1.12

# offered_byOwner 0.23 0.14 0.37

# breeder_org_affiliationyes 1.56 0.93 2.65

# age_bin1 - 3 years 0.08 0.03 0.17

# age_bin3 + years 0.03 0.00 0.21

# age_bin6 months - 1 year 0.11 0.04 0.25

# age_bin8 weeks - 6 months 0.32 0.21 0.48

# price_bin$2000+ 0.53 0.26 1.06

# price_bin$501 - $2000 1.24 0.72 2.15

# price_binFree 0.52 0.07 2.29

# price_binUnknown 0.50 0.15 1.54

lrtest(model_BID_3, model_BID_2)

# Likelihood ratio test

#

# Model 1: state_registered ~ pure_cross + offered_by + breeder_org_affiliation +

# age_bin + price_bin

# Model 2: state_registered ~ vaccination + pure_cross + offered_by + breeder_org_affiliation +

# age_bin + price_bin

# #Df LogLik Df Chisq Pr(>Chisq)

# 1 14 -323.72

# 2 15 -321.04 1 5.3585 0.02062 *

# ---

# Signif. codes: 0 ‘***’ 0.001 ‘**’ 0.01 ‘*’ 0.05 ‘.’ 0.1 ‘ ’ 1

#vaccinatin is sig

#model4 - model2 minus pure)cross

model_BID_4 <- glm(state_registered ~ vaccination +

offered_by + breeder_org_affiliation +

age_bin + price_bin, family = binomial,

data = dogadsID2)

summary(model_BID_4)

# Call:

# glm(formula = state_registered ~ vaccination + offered_by + breeder_org_affiliation +

# age_bin + price_bin, family = binomial, data = dogadsID2)

#

# Deviance Residuals:

# Min 1Q Median 3Q Max

# -2.2858 -0.7414 0.4698 0.7868 2.6157

#

# Coefficients:

# Estimate Std. Error z value Pr(>|z|)

# (Intercept) 1.2165 0.3528 3.449 0.000563 ***

# vaccinationyes 0.6514 0.2680 2.431 0.015076 *

# offered_byOwner -1.4037 0.2410 -5.824 5.74e-09 ***

# breeder_org_affiliationyes 0.3878 0.2660 1.458 0.144914

# age_bin1 - 3 years -2.5304 0.4336 -5.836 5.35e-09 ***

# age_bin3 + years -3.4222 1.1412 -2.999 0.002710 **

# age_bin6 months - 1 year -2.1839 0.4503 -4.850 1.24e-06 ***

# age_bin8 weeks - 6 months -1.1346 0.2118 -5.356 8.50e-08 ***

# price_bin$2000+ -0.5258 0.3242 -1.622 0.104777

# price_bin$501 - $2000 0.2806 0.2606 1.077 0.281641

# price_binFree -0.6701 0.8335 -0.804 0.421430

# price_binUnknown -0.5846 0.5888 -0.993 0.320781

# ---

# Signif. codes: 0 ‘***’ 0.001 ‘**’ 0.01 ‘*’ 0.05 ‘.’ 0.1 ‘ ’ 1

#

# (Dispersion parameter for binomial family taken to be 1)

#

# Null deviance: 883.44 on 647 degrees of freedom

# Residual deviance: 646.28 on 636 degrees of freedom

# AIC: 670.28

#

# Number of Fisher Scoring iterations: 5

BIN_dci <- confint(model_BID_4)

round(exp(cbind(model_BID_4$coef, BIN_dci)), digits = 2)

# 2.5 % 97.5 %

# (Intercept) 3.38 1.71 6.82

# vaccinationyes 1.92 1.14 3.26

# offered_byOwner 0.25 0.15 0.39

# breeder_org_affiliationyes 1.47 0.88 2.49

# age_bin1 - 3 years 0.08 0.03 0.18

# age_bin3 + years 0.03 0.00 0.22

# age_bin6 months - 1 year 0.11 0.04 0.26

# age_bin8 weeks - 6 months 0.32 0.21 0.48

# price_bin$2000+ 0.59 0.31 1.11

# price_bin$501 - $2000 1.32 0.79 2.20

# price_binFree 0.51 0.07 2.21

# price_binUnknown 0.56 0.17 1.72

lrtest(model_BID_4, model_BID_2)

# Likelihood ratio test

#

# Model 1: state_registered ~ vaccination + offered_by + breeder_org_affiliation +

# age_bin + price_bin

# Model 2: state_registered ~ vaccination + pure_cross + offered_by + breeder_org_affiliation +

# age_bin + price_bin

# #Df LogLik Df Chisq Pr(>Chisq)

# 1 12 -323.14

# 2 15 -321.04 3 4.1931 0.2414

#pure_CRoss not sig

#model 5: model 4 minus offered by

model_BID_5 <- glm(state_registered ~ vaccination +

breeder_org_affiliation +

age_bin + price_bin, family = binomial,

data = dogadsID2)

summary(model_BID_5)

# #Call:

# glm(formula = state_registered ~ vaccination + breeder_org_affiliation +

# age_bin + price_bin, family = binomial, data = dogadsID2)

#

# Deviance Residuals:

# Min 1Q Median 3Q Max

# -2.2145 -0.8082 0.5791 0.8162 2.6607

#

# Coefficients:

# Estimate Std. Error z value Pr(>|z|)

# (Intercept) 0.12286 0.27817 0.442 0.65873

# vaccinationyes 0.82175 0.25627 3.207 0.00134 **

# breeder_org_affiliationyes 0.83811 0.24755 3.386 0.00071 ***

# age_bin1 - 3 years -2.86075 0.42188 -6.781 1.19e-11 ***

# age_bin3 + years -3.48658 1.11134 -3.137 0.00171 **

# age_bin6 months - 1 year -2.47526 0.43351 -5.710 1.13e-08 ***

# age_bin8 weeks - 6 months -1.09522 0.20405 -5.367 7.99e-08 ***

# price_bin$2000+ -0.08185 0.29994 -0.273 0.78493

# price_bin$501 - $2000 0.57920 0.24264 2.387 0.01699 *

# price_binFree -0.77229 0.84754 -0.911 0.36218

# price_binUnknown -0.57523 0.57169 -1.006 0.31432

# ---

# Signif. codes: 0 ‘***’ 0.001 ‘**’ 0.01 ‘*’ 0.05 ‘.’ 0.1 ‘ ’ 1

#

# (Dispersion parameter for binomial family taken to be 1)

#

# Null deviance: 883.44 on 647 degrees of freedom

# Residual deviance: 681.57 on 637 degrees of freedom

# AIC: 703.57

#

# Number of Fisher Scoring iterations: 5

BIN_dci <- confint(model_BID_5)

round(exp(cbind(model_BID_5$coef, BIN_dci)), digits = 2)

# 2.5 % 97.5 %

# (Intercept) 1.13 0.65 1.95

# vaccinationyes 2.27 1.38 3.78

# breeder_org_affiliationyes 2.31 1.43 3.78

# age_bin1 - 3 years 0.06 0.02 0.12

# age_bin3 + years 0.03 0.00 0.19

# age_bin6 months - 1 year 0.08 0.03 0.19

# age_bin8 weeks - 6 months 0.33 0.22 0.50

# price_bin$2000+ 0.92 0.51 1.66

# price_bin$501 - $2000 1.78 1.11 2.88

# price_binFree 0.46 0.06 2.04

# price_binUnknown 0.56 0.17 1.68

lrtest(model_BID_5, model_BID_4)

# #Likelihood ratio test

#

# Model 1: state_registered ~ vaccination + breeder_org_affiliation + age_bin +

# price_bin

# Model 2: state_registered ~ vaccination + offered_by + breeder_org_affiliation +

# age_bin + price_bin

# #Df LogLik Df Chisq Pr(>Chisq)

# 1 11 -340.79

# 2 12 -323.14 1 35.297 2.831e-09 ***

# ---

# Signif. codes: 0 ‘***’ 0.001 ‘**’ 0.01 ‘*’ 0.05 ‘.’ 0.1 ‘ ’ 1

#seller is sig

#model 6: model 4 -

model_BID_6 <- glm(state_registered ~ vaccination +

offered_by +

age_bin + price_bin, family = binomial,

data = dogadsID2)

summary(model_BID_6)

# #Call:

# glm(formula = state_registered ~ vaccination + offered_by + age_bin +

# price_bin, family = binomial, data = dogadsID2)

#

# Deviance Residuals:

# Min 1Q Median 3Q Max

# -2.1947 -0.7884 0.4342 0.7277 2.6229

#

# Coefficients:

# Estimate Std. Error z value Pr(>|z|)

# (Intercept) 1.2706 0.3531 3.598 0.00032 ***

# vaccinationyes 0.6906 0.2673 2.583 0.00978 **

# offered_byOwner -1.5124 0.2304 -6.563 5.27e-11 ***

# age_bin1 - 3 years -2.5008 0.4313 -5.799 6.69e-09 ***

# age_bin3 + years -3.2906 1.1258 -2.923 0.00347 **

# age_bin6 months - 1 year -2.2060 0.4488 -4.916 8.85e-07 ***

# age_bin8 weeks - 6 months -1.1206 0.2115 -5.299 1.16e-07 ***

# price_bin$2000+ -0.3266 0.2932 -1.114 0.26517

# price_bin$501 - $2000 0.3529 0.2568 1.374 0.16929

# price_binFree -0.6646 0.8339 -0.797 0.42546

# price_binUnknown -0.5760 0.5898 -0.977 0.32879

# ---

# Signif. codes: 0 ‘***’ 0.001 ‘**’ 0.01 ‘*’ 0.05 ‘.’ 0.1 ‘ ’ 1

#

# (Dispersion parameter for binomial family taken to be 1)

#

# Null deviance: 883.44 on 647 degrees of freedom

# Residual deviance: 648.41 on 637 degrees of freedom

# AIC: 670.41

#

# Number of Fisher Scoring iterations: 5

BIN_dci <- confint(model_BID_6)

round(exp(cbind(model_BID_6$coef, BIN_dci)), digits = 2)

# 2.5 % 97.5 %

# (Intercept) 3.56 1.80 7.20

# vaccinationyes 1.99 1.18 3.38

# offered_byOwner 0.22 0.14 0.34

# age_bin1 - 3 years 0.08 0.03 0.18

# age_bin3 + years 0.04 0.00 0.24

# age_bin6 months - 1 year 0.11 0.04 0.25

# age_bin8 weeks - 6 months 0.33 0.21 0.49

# price_bin$2000+ 0.72 0.40 1.27

# price_bin$501 - $2000 1.42 0.86 2.35

# price_binFree 0.51 0.07 2.23

# price_binUnknown 0.56 0.17 1.74

lrtest(model_BID_6, model_BID_4)

# Likelihood ratio test

# Likelihood ratio test

#

# Model 1: state_registered ~ vaccination + offered_by + age_bin + price_bin

# Model 2: state_registered ~ vaccination + offered_by + breeder_org_affiliation +

# age_bin + price_bin

# #Df LogLik Df Chisq Pr(>Chisq)

# 1 11 -324.21

# 2 12 -323.14 1 2.1356 0.1439

#breeder org not sig

#mdel 4 minus age

#Model 7 - model 6 minus age

model_BID_7 <- glm(state_registered ~ vaccination +

offered_by +

price_bin, family = binomial,

data = dogadsID2)

summary(model_BID_7)

# Call:

# glm(formula = state_registered ~ vaccination + offered_by + price_bin,

# family = binomial, data = dogadsID2)

#

# Deviance Residuals:

# Min 1Q Median 3Q Max

# -1.8776 -0.9554 0.6136 0.8292 2.4645

#

# Coefficients:

# Estimate Std. Error z value Pr(>|z|)

# (Intercept) 0.4478 0.3057 1.465 0.14289

# vaccinationyes 0.7384 0.2456 3.007 0.00264 **

# offered_byOwner -1.7339 0.2104 -8.239 < 2e-16 ***

# price_bin$2000+ -0.2953 0.2724 -1.084 0.27835

# price_bin$501 - $2000 0.3881 0.2350 1.651 0.09865 .

# price_binFree -1.7016 0.7789 -2.185 0.02891 *

# price_binUnknown -0.5586 0.5671 -0.985 0.32464

# ---

# Signif. codes: 0 ‘***’ 0.001 ‘**’ 0.01 ‘*’ 0.05 ‘.’ 0.1 ‘ ’ 1

#

# (Dispersion parameter for binomial family taken to be 1)

#

# Null deviance: 883.44 on 647 degrees of freedom

# Residual deviance: 724.86 on 641 degrees of freedom

# AIC: 738.86

#

# Number of Fisher Scoring iterations: 5

BIN_dci <- confint(model_BID_7)

round(exp(cbind(model_BID_7$coef, BIN_dci)), digits = 2)

#

# 2.5 % 97.5 %

# (Intercept) 1.56 0.86 2.86

# vaccinationyes 2.09 1.30 3.40

# offered_byOwner 0.18 0.12 0.27

# price_bin$2000+ 0.74 0.43 1.26

# price_bin$501 - $2000 1.47 0.93 2.33

# price_binFree 0.18 0.03 0.68

# price_binUnknown 0.57 0.18 1.67

lrtest(model_BID_7, model_BID_6)

# Likelihood ratio test

#

# Model 1: state_registered ~ vaccination + offered_by + price_bin

# Model 2: state_registered ~ vaccination + offered_by + age_bin + price_bin

# #Df LogLik Df Chisq Pr(>Chisq)

# 1 7 -362.43

# 2 11 -324.21 4 76.444 9.862e-16 ***

# ---

# Signif. codes: 0 ‘***’ 0.001 ‘**’ 0.01 ‘*’ 0.05 ‘.’ 0.1 ‘ ’ 1

#age is sig

#model 8 : model 6 minus price

model_BID_8 <- glm(state_registered ~ vaccination +

offered_by +

age_bin, family = binomial,

data = dogadsID2)

summary(model_BID_8)

# Call:

# glm(formula = state_registered ~ vaccination + offered_by + age_bin,

# family = binomial, data = dogadsID2)

#

# Deviance Residuals:

# Min 1Q Median 3Q Max

# -2.0465 -0.6838 0.5128 0.8444 2.3867

#

# Coefficients:

# Estimate Std. Error z value Pr(>|z|)

# (Intercept) 1.2292 0.2895 4.246 2.18e-05 ***

# vaccinationyes 0.7333 0.2612 2.807 0.00500 **

# offered_byOwner -1.4486 0.2032 -7.131 9.99e-13 ***

# age_bin1 - 3 years -2.5691 0.4231 -6.072 1.27e-09 ***

# age_bin3 + years -3.3654 1.0866 -3.097 0.00195 **

# age_bin6 months - 1 year -2.2517 0.4408 -5.108 3.25e-07 ***

# age_bin8 weeks - 6 months -1.1146 0.2097 -5.316 1.06e-07 ***

# ---

# Signif. codes: 0 ‘***’ 0.001 ‘**’ 0.01 ‘*’ 0.05 ‘.’ 0.1 ‘ ’ 1

#

# (Dispersion parameter for binomial family taken to be 1)

#

# Null deviance: 883.44 on 647 degrees of freedom

# Residual deviance: 658.83 on 641 degrees of freedom

# AIC: 672.83

#

# Number of Fisher Scoring iterations: 5

BIN_dci <- confint(model_BID_8)

round(exp(cbind(model_BID_8$coef, BIN_dci)), digits = 2)

# 2.5 % 97.5 %

# (Intercept) 3.42 1.95 6.08

# vaccinationyes 2.08 1.25 3.49

# offered_byOwner 0.23 0.16 0.35

# age_bin1 - 3 years 0.08 0.03 0.17

# age_bin3 + years 0.03 0.00 0.20

# age_bin6 months - 1 year 0.11 0.04 0.24

# age_bin8 weeks - 6 months 0.33 0.22 0.49

lrtest(model_BID_8, model_BID_6)

# Likelihood ratio test

#

# Model 1: state_registered ~ vaccination + offered_by + age_bin

# Model 2: state_registered ~ vaccination + offered_by + age_bin + price_bin

# #Df LogLik Df Chisq Pr(>Chisq)

# 1 7 -329.42

# 2 11 -324.21 4 10.418 0.03395 *

# ---

# Signif. codes: 0 ‘***’ 0.001 ‘**’ 0.01 ‘*’ 0.05 ‘.’ 0.1 ‘ ’ 1

#price is sig - FINAL MODEL IS 6!

#RR FOR FINAL MODEL:

modelbid6_ra_RR<-odds_to_rr(model_BID_6)

format(round(modelbid6_ra_RR,2),nsmall=2)

# # RR lower.ci upper.ci

# (Intercept) 1.44 1.23 1.58

# vaccinationyes 1.27 1.07 1.43

# offered_byOwner 0.40 0.28 0.55

# age_bin1 - 3 years 0.17 0.07 0.34

# age_bin3 + years 0.08 0.00 0.43

# age_bin6 months - 1 year 0.23 0.10 0.45

# age_bin8 weeks - 6 months 0.53 0.39 0.69

# price_bin$2000+ 0.86 0.61 1.10

# price_bin$501 - $2000 1.14 0.93 1.32

# price_binFree 0.71 0.16 1.31

# price_binUnknown 0.75 0.32 1.22

######BID RR PLOTS########

## Libs ----

pacman::p_load(tidyverse)

## Set up data ----

plot_data <- tibble(

groups = c("not vaccinated (ref)", "vaccinated",

"offered by breeder (ref)", "offered by owner",

"price: free", "price: $1 - $500 (ref)", "price:$501 - $2000", "price:$2000+",

"age:<8 weeks (ref)", "age:8 weeks - 6 months", "age:6 months-1 year",

"age:1-3 years", "age:3+ years"),

OR = c(1.00, 1.27,

1.00, 0.40,

0.71, 1.00, 1.14, 0.86,

1.00, 0.53, 0.23,

0.17, 0.08),

lwr = c(1.00, 1.07,

1.00, 0.28,

0.16, 1.00, 0.93, 0.61,

1.00, 0.39, 0.10,

0.07, 0.00),

upr = c(1.00, 1.43,

1.00, 0.55,

1.31, 1.00, 1.32, 1.10,

1.00, 0.69, 0.45,

0.34, 0.43)

)

## Set order of groups ----

plot_data <-

plot_data %>%

mutate(groups = fct_rev(fct_inorder(groups)))

## Make plot ----

plot_data %>%

ggplot(aes(OR, groups)) +

geom_vline(aes(xintercept = 1.0), size = 1) +

geom_errorbarh(aes(xmin = lwr, xmax = upr),

size = 0.5, height = 0.2, color = "gray50") +

geom_point(size = 3.5, color = "grey40") +

scale_x_continuous(breaks = seq(0.1, 2.0, 0.2),

labels = seq(0.1, 2.0, 0.2),

limits = c(0.00,2.0)) +

theme_bw() +

labs(

y = "Factors associated with breeder ID number",

x = "Est. Relative Risk (RR)"

) +

geom_hline(yintercept=c(5.5, 9.5, 11.5), linetype="dotted",color = "grey10", size=0.5)

#######microchip RR plot#################

## Libs ----

pacman::p_load(tidyverse)

## Set up data ----

plot_data <- tibble(

groups = c("VIC (ref)", "QLD", "NSW", "TAS", "SA", "WA",

"not vaccinated (ref)", "vaccinated",

"offered by breeder (ref)", "offered by owner",

"price: free", "price: $1 - $500 (ref)", "price:$501 - $2000", "price:$2001+"),

OR = c(1.00, 0.33, 0.31, 0.16, 0.17, 0.22,

1.00, 1.40,

1.00, 0.74,

0.78, 1.00, 1.10, 1.23),

lwr = c(1.00, 0.17, 0.16, 0.06, 0.07, 0.10,

1.00, 1.39,

1.00, 0.60,

0.55, 1.00, 0.99, 1.12),

upr = c(1.00, 0.55, 0.51, 0.36, 0.37, 0.42,

1.00, 1.40,

1.00, 0.88,

0.99, 1.00, 1.18, 1.31)

)

## Set order of groups ----

plot_data <-

plot_data %>%

mutate(groups = fct_rev(fct_inorder(groups)))

## Make plot ----

plot_data %>%

ggplot(aes(OR, groups)) +

geom_vline(aes(xintercept = 1.0), size = 1) +

geom_errorbarh(aes(xmin = lwr, xmax = upr),

size = 0.5, height = 0.2, color = "gray50") +

geom_point(size = 3.5, color = "grey40") +

scale_x_continuous(breaks = seq(0.1, 2.0, 0.2),

labels = seq(0.1, 2.0, 0.2),

limits = c(0.00,1.5)) +

theme_bw() +

labs(

y = "Factors associated with microchip",

x = "Est. Relative Risk (RR)"

) +

geom_hline(yintercept=c(4.5, 6.5, 8.5), linetype="dotted",color = "grey10", size=0.5)

####### desexing RR plot #################

## Libs ----

pacman::p_load(tidyverse)

## Set up data ----

plot_data <- tibble(

groups = c("not microchipped (ref)", "microchipped",

"cross breed (ref)", "pure breed", "designer breed",

"price: free", "price: $1 - $500 (ref)", "price:$501 - $2000", "price: $2001+",

"age:6 months-1 year", "age:1-3 years (ref)", "age:3+ years"),

OR = c(1.00, 2.68,

1.00, 0.73, 2.47,

1.11, 1.00, 0.70, 0.30,

0.43,1.00, 1.52),

lwr = c(1.00, 2.10,

1.00, 0.45, 0.96,

0.64, 1.00, 0.41, 0.11,

0.23, 1.00, 1.00),

upr = c(1.00, 3.25,

1.00, 1.13, 3.74,

1.74, 1.00, 1.14, 0.70,

0.74,1.00, 2.14)

)

## Set order of groups ----

plot_data <-

plot_data %>%

mutate(groups = fct_rev(fct_inorder(groups)))

## Make plot ----

plot_data %>%

ggplot(aes(OR, groups)) +

geom_vline(aes(xintercept = 1.0), size = 1) +

geom_errorbarh(aes(xmin = lwr, xmax = upr),

size = 0.5, height = 0.2, color = "gray50") +

geom_point(size = 3.5, color = "grey40") +

scale_x_continuous(breaks = seq(0.1, 4.0, 0.2),

labels = seq(0.1, 4.0, 0.2),

limits = c(0.1,4.0)) +

theme_bw() +

labs(

y = "Factors associated with desexing",

x = "Est. Relative Risk (RR)"

) +

geom_hline(yintercept=c(3.5, 7.5, 10.5), linetype="dotted",color = "grey10", size=0.5)
